# From PERK to RIPK1: Design, synthesis and evaluation of novel potent and selective necroptosis inhibitors

**DOI:** 10.3389/fchem.2023.1160164

**Published:** 2023-04-07

**Authors:** Camilla Scarpellini, Sophie Valembois, Kenneth Goossens, Mike Vadi, Caroline Lanthier, Greta Klejborowska, Pieter Van Der Veken, Hans De Winter, Mathieu J. M. Bertrand, Koen Augustyns

**Affiliations:** ^1^ Laboratory of Medicinal Chemistry, Department of Pharmaceutical Sciences, Faculty of Pharmaceutical, Biomedical and Veterinary Sciences, University of Antwerp, Antwerp, Belgium; ^2^ Vlaams Instituut voor Biotechnologie (VIB) Center for Inflammation Research, Ghent, Belgium; ^3^ Laboratory Cell Death and Inflammation, Department of Biomedical Molecular Biology, Ghent University, Ghent, Belgium

**Keywords:** RIPK1 inhibitor, necroptosis, regulated cell death, inflammation, type II kinase inhibitor, PERK (PKR-like endoplasmic reticulum kinase)

## Abstract

Receptor-Interacting serine/threonine-Protein Kinase 1 (RIPK1) emerged as an important driver of inflammation and, consequently, inflammatory pathologies. The enzymatic activity of RIPK1 is known to indirectly promote inflammation by triggering cell death, in the form of apoptosis, necroptosis and pyroptosis. Small molecule Receptor-Interacting serine/threonine-Protein Kinase 1 inhibitors have therefore recently entered clinical trials for the treatment of a subset of inflammatory pathologies. We previously identified GSK2656157 (GSK’157), a supposedly specific inhibitor of protein kinase R (PKR)-like ER kinase (PERK), as a much more potent type II Receptor-Interacting serine/threonine-Protein Kinase 1 inhibitor. We now performed further structural optimisation on the GSK’157 scaffold in order to develop a novel class of more selective Receptor-Interacting serine/threonine-Protein Kinase 1 inhibitors. Based on a structure-activity relationship (SAR) reported in the literature, we anticipated that introducing a substituent on the *para-*position of the pyridinyl ring would decrease the interaction with PERK. Herein, we report a series of novel GSK’157 analogues with different *para*-substituents with increased selectivity for Receptor-Interacting serine/threonine-Protein Kinase 1. The optimisation led to UAMC-3861 as the best compound of this series in terms of activity and selectivity for Receptor-Interacting serine/threonine-Protein Kinase 1 over PERK. The most selective compounds were screened *in vitro* for their ability to inhibit RIPK1-dependent apoptosis and necroptosis. With this work, we successfully synthesised a novel series of potent and selective type II Receptor-Interacting serine/threonine-Protein Kinase 1 inhibitors based on the GSK’157 scaffold.

## 1 Introduction

Cell death is increasingly recognised as an important driver of inflammation, and is consequently believed to be at the origin of various inflammatory diseases when improperly regulated (Newton et al., 2021). A subset of innate immune receptors can trigger the dismantlement of the cell, and the resulting cell death can promote the production of pro-inflammatory mediators by activating immune receptors on neighbouring effector cells. Next to apoptosis, which is generally considered immunologically silent, lytic forms of cell death, such as apoptosis-driven secondary necrosis, necroptosis and pyroptosis, release intracellular factors, known as danger-associated molecular patterns (DAMPs), that activate immune receptors and induce inflammatory responses. In addition, the death of epithelial cells can also affect the functionality of bodily barriers, and thereby trigger/exacerbate inflammation through the sensing of pathogen-associated molecular patterns (PAMPs) from microbes that have breached the barriers. Accumulating evidence indicates that genetic targeting of cell death can revert the inflammatory pathology state in various mouse models of acute and chronic inflammatory diseases. Hence, drugs that inhibit cell death are currently under investigation as potential therapies for human inflammatory and autoimmune diseases. Among these drugs, pharmacological inhibitors of the receptor-interacting serine/threonine protein kinase 1 (RIPK1) have entered clinical trials for the treatment of autoimmune diseases (psoriasis, ulcerative colitis and rheumatoid arthritis), as well as some cancers and neurological disorders (Mifflin et al., 2020).

RIPK1 functions as a central signalling node downstream of several immune receptors, where it can paradoxically function as a scaffold to promote cell survival or as an active kinase to trigger cell death (Delanghe et al., 2020). RIPK1 has been best studied in the context of tumour necrosis factor (TNF) signalling. Binding of TNF to TNFR1 induces recruitment of RIPK1 to the cytosolic tail of the receptor, an initial step in the assembly of the receptor signalling complex (Complex I). Within this complex, RIPK1 functions as a ubiquitylated scaffold that contributes to the activation of the MAPK and NF-κB signalling pathways, which collectively promote transcription of pro-inflammatory and pro-survival genes. Kinases from the MAPK and NF-κB pathway, including MK2 and IKKα/β, signal back to RIPK1 to maintain it in an inactive prosurvival state (Dondelinger et al., 2015; 2017; 2019; Jaco et al., 2017; Menon et al., 2017; Lafont et al., 2018; Xu et al., 2018). Consequently, conditions that affect proper activation of these kinases remove the brake on RIPK1, which can then autophosphorylate on Ser166, dissociate from complex I and promote assembly of the cytosolic caspase-8 activating complex (complex II) that triggers apoptosis induction. Caspase-8 activation by RIPK1 was also recently reported to induce cleavage and activation of the pore forming molecule gasdermin D (GSDMD) in macrophages, thereby promoting induction of a specific form of pyroptosis (Orning et al., 2018; Chen et al., 2021). Furthermore, conditions that prevent activation of caspase-8 in Complex II (e.g., by the use of the pan-caspase inhibitor zVAD.fmk) induce further recruitment of RIPK3 and MLKL to complex II, now called the necrosome, leading to RIPK3 activation, phosphorylation of MLKL by RIPK3, and subsequent necroptosis induction (Rodriguez et al., 2016; Mompea et al., 2018; Weber et al., 2018; Klöditz and Fadeel, 2019)^.^ The enzymatic activity of RIPK1 is therefore capable of inducing three distinct forms of cell death, which have all been linked to inflammation (Mifflin et al., 2020). Consequently, the importance of specific targeting RIPK1 to prevent chronic inflammatory processes has emerged.

**FIGURE 1:**
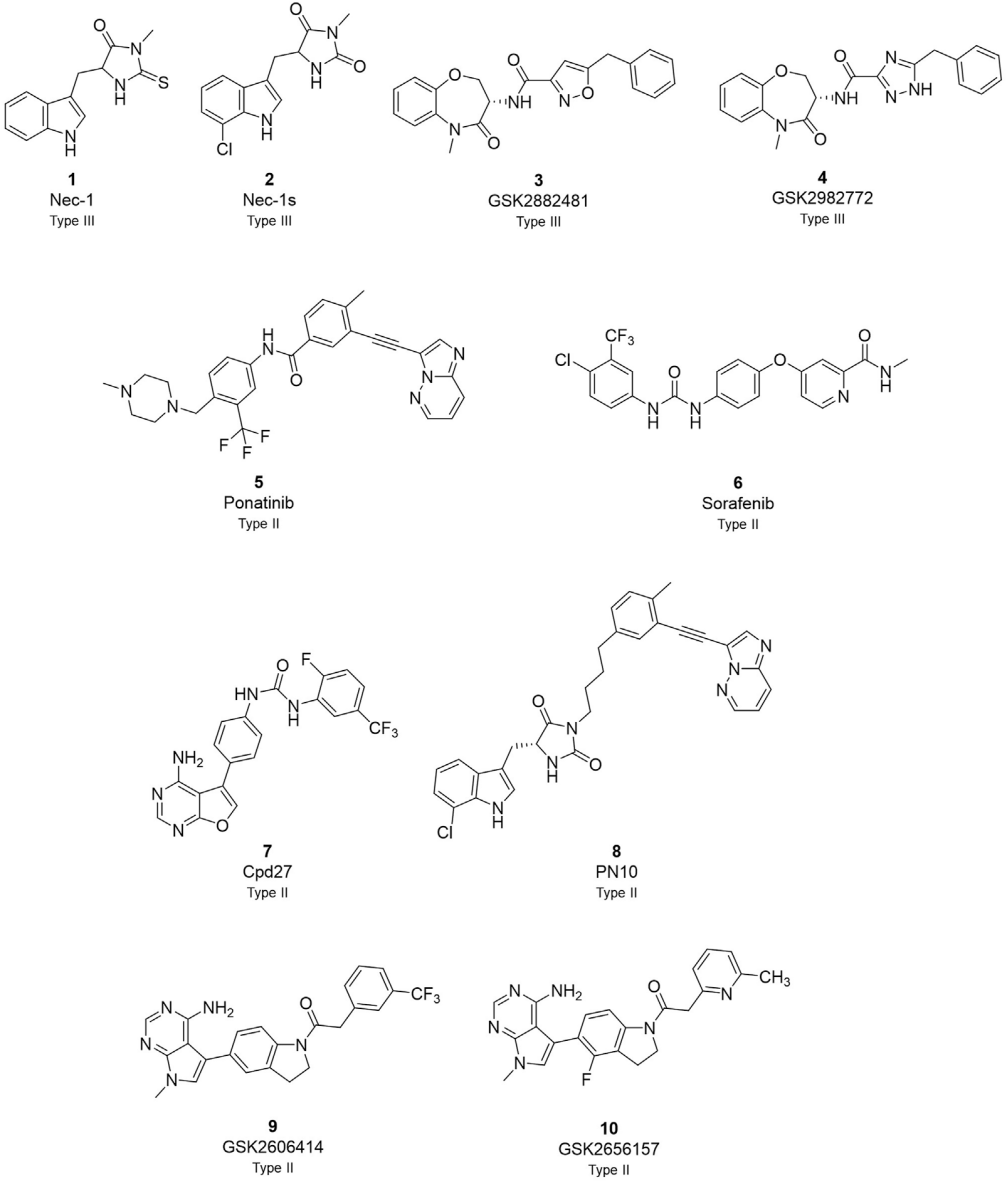
Overview of currently known RIPK1 inhibitors.

The first RIPK1 inhibitor described in the literature was necrostatin-1 (Nec-1) in 2005 (Figure 1) (Degterev et al., 2005). It served as a valuable tool compound to study RIPK1 structure and to demonstrate the implication of RIPK1 kinase-dependent cell death in animal models of human diseases (Xie et al., 2013). Particularly, the co-crystallization of Nec-1s, a stable form of Nec-1, with the kinase domain of RIPK1 demonstrated the typical type III kinases inhibitor mode of interaction, with binding to the specific RIPK1 allosteric pocket formed by the DLG-out conformation and without interacting with the hinge region (Wang et al., 2006; Zheng et al., 2008; Takahashi et al., 2012). Since then, several additional RIPK1 inhibitors have been reported (Martens et al., 2020; Chen et al., 2022; Shi et al., 2022). Among them, a class of compounds featuring a benzoxazepinone core emerged with GSK2882481 (Clark et al., 2009; Harris et al., 2016), but interspecies differences were observed when comparing humans to non-primate RIPK1, probably due to the different amino acids featured by the enzyme affecting protein flexibility (Berger et al., 2015; Harris et al., 2017). This class of compound was further optimised aiming to develop a drug with suitable oral bioavailability. The lead optimisation led to the discovery of GSK2982772 (GSK’772) which is currently in phase IIb clinical trial for the treatment of ulcerative colitis, psoriasis, and rheumatoid arthritis (Weisel et al., 2017).

Among the different RIPK1-inhibitors available in the literature (Figure 1), the type II class has been less investigated in disease models and in clinic (Martens et al., 2020; Chen et al., 2022; Shi et al., 2022). In the type II RIPK1 inhibitors family, tyrosine kinase inhibitor (TKI) ponatinib and its analogues, and sorafenib are reported as dual inhibitors for both RIPK1 and RIPK3 (Fauster et al., 2015; Martens et al., 2017). Compound 27 (cpd27) showed promising pharmacokinetics properties but low kinase selectivity (Harris et al., 2013). PN10 is a hybrid compound which combines the structures of Nec-1 and ponatinib (Najjar et al., 2015). This strategy increased specificity and selectivity of the analogue retaining the interaction with the allosteric pocket of Nec-1 and the interaction with the hinge region of ponatinib as type II kinases inhibitors. Besides, GSK identified a class of dual inhibitors represented by GSK2593067 and GSK2593074 which completely blocked necroptosis in both human and murine cells as type II inhibitors (Zhou et al., 2019).

**FIGURE 2:**
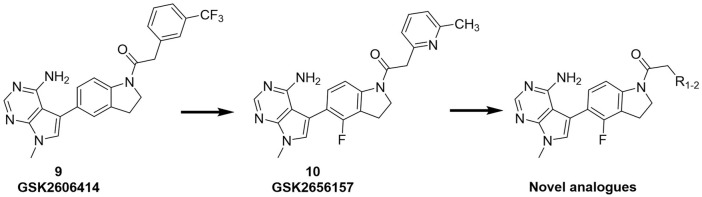
Overview of the novel GSK’157 analogues featuring a substituent on corresponding para-position or the pyridinyl or aryl moiety.

We previously reported that two commonly used Protein kinase R (PKR)-like ER kinase (PERK) inhibitors, GSK2606414 (GSK’414) and GSK2656157 *(*GSK’157*),* were actually potent inhibitors of RIPK1 (Rojas-Rivera et al., 2017). Interestingly, and in comparison to the above mentioned RIPK1 inhibitors, GSK’157 appeared to function as a type II kinases inhibitor of RIPK1 (Axten et al., 2013)**.** A kinome scan perfomed for both, GSK’414 and GSK’157 suggested a good selectivity profile. GSK’414 was screened against a panel of 294 kinases at 10 µM with an inhibition of 20 kinases with more than 85%, while the optimised analogue GSK’157 inhibited 17 out of 300 kinases with more than 80% at the same concentration. The reported structure-activity relationship (SAR) suggested that derivatisation of the corresponding *para*-position of the pyridinyl ring could reduce affinity for PERK (Axten et al., 2012; 2013). Moreover, recent molecular modelling studies confirmed the different nature of the C-alpha helix domain in RIPK1 with the presence of an additional lipophilic pocket compared to PERK (Chintha et al., 2019). We investigated the structural differences between PERK and RIPK1 and we verified the presence of an extra pocket with additional space to place the *para*-substituent. We hypothesised that this *para*-substituent would reduce the affinity for PERK and at the same time, keep or enhance the affinity for RIPK1. Based on these assumptions, we herein report the design, synthesis and biological evaluation of novel GSK’157 analogues with increased selectivity for RIPK1 over PERK (Figure 2)**.**

## 2 Materials and methods

### 2.1 Chemicals and instruments

Unless otherwise stated, laboratory reagent grade solvents were used. Reagents were obtained from various commercial sources and were used without any prior purification. Characterisation of all compounds was done with ^1^H and ^13^C NMR and mass spectrometry. NMR spectra were recorded with a 400 MHz Bruker Avance III Nanobay spectrometer with Ultrashield. All obtained spectra were analysed using MestReNova analytical chemistry software. Chemical shifts are displayed in ppm and coupling constants are shown in hertz (Hz). ES mass spectra were obtained from an Esquire 3000plus Ion Trap Mass Spectrometer from Bruker Daltonics. The UPLC (ultra performance liquid chromatography), used to quantify the purity of the products, was an ACQUITY UPLC H-Class system with a TUV detector Waters coupled to an MS detector Waters Qda. Waters Acquity UPLC BEH C18 1.7 μm, 2.1 mm × 50 mm column was used. The eluent was composed of two different solvents. Solvent C consisted of water with 0.1% formic acid, solvent D was acetonitrile. For most of the experiments, unless stated otherwise, the general method was used. The column was first equilibrated for 0.15 min with a mixture of 95% solvent C and 5% solvent D. After that, solvent D was increased linearly to 100% over 1.75 min before being held constant for 0.25 min, followed by a mixture of 95% solvent C and 5% solvent D for 0.75 min (flow rate 0.7 mL/min). All mass spectra were recorded over a *m/z* range of 100–1000. The wavelength for UV detection was 254 nm. Method II starts with equilibration of column for 0.15 min with a mixture of 95% solvent C and 5% solvent D. After that, solvent D was increased linearly to 100% over 2.50 min before being held constant for 0.75 min, followed by a mixture of 95% solvent C and 5% solvent D for 0.75 min (flow rate 0.7 mL/min). Key target compounds for the activity were analyzedby high resolution mass spectrometry: 10 μL of each sample (concentration = 10^−5^ M) was injected using the CapLC system (Waters, Manchester, United Kingdom) and electrosprayed using a standard electrospray source. Samples were injected with an interval of 5 min. Positive ion mode accurate mass spectra (HRMS) were acquired usinga Q-TOF II instrument (Waters, Manchester, United Kingdom). The MS was calibrated prior to use with a 0.2% H_3_PO_4_ solution. The spectra were lock mass corrected using the knownmassof the nearest H_3_PO_4_ cluster. During the chemical synthesis, flash purification was performed when necessary on a Biotage ISOLERA One flash system equipped with an internal variable dual-wavelength diode array detector (200–400 nm). For the normal phase, purifications SNAP cartridges (10–340 g, flow rate of 10–100 mL/min) were used and reversed-phase purifications were done making use of KP-C18 or Buchi EcoFlex C18 cartridges. Dry sample loading was done by self-packing sample cartridges using Celite^®^ 545. Gradients used varied for each purification. The following section comprises the synthetic procedures and analytical data for all compounds reported in this manuscript. Several synthesis procedures that were used in the preparation of intermediates and final products are summarized here as “General Procedures”. The purities of all final products were found to be >95%. Analyses indicated by the symbols of the elements or functions were within ±0.4% of the theoretical values.

### 2.2 Antibodies and reagents

Antibodies and reagents were purchased from the following companies: anti-PERK (Cell Signaling Technology; no. 3192), anti-RIPK1 (BD Biosciences Laboratories; no. 610459), pPERK (Cell Signaling Technology; no. #3179), pRIPK1 (Cell Signaling Technology; no. #31122 (for S166) or ThermoFischer, custom made (for S166/T169)), Tunicamicyn (Tm; Sigma-Aldrich, St Louis, MO, United States; no. T7765), Tubulin (Abacam, no. ab21058), 9-Epimer-11,12-dihydro-(5Z)-7- Oxozeanol ((5Z)-7-Oxozeaenol or TAK1 inhibitor (TAK1i); AnalytiCon Discovery GmbH, Potsdam, Germany; no. NP-0009245), Carbobenzoxy-valyl-alanyl-aspartyl-[O-methyl]-fluoromethylketone or zVAD.fmk (Bachem, Bubendorf, Switzerland; no. N-1510), GSK2656157 (ApexBio, Boston, MA, United States; no. B2175.). Recombinant human TNF-α is produced and purified to at least 99% homogeneity in the laboratory of VIB in Gent.

### 2.3 Cell culture

Mouse embryonic fibroblasts (MEFs) were isolated from C57Bl6/J E12.5 embryos according to standard protocol and immortalized by transfection of an SV40 large T-expressing construct. Ripk3^+/+^ and Ripk3^−/−^ MEFs have been isolated from littermate embryos of Ripk3 ± pregnant females and have been described previously (Vanlangenakker et al., 2011; Dondelinger et al., 2013). The MEFs cells were cultured in Dulbecco’s modified Eagle’s medium supplemented with 10% fetal calf serum, L-glutamine (200 mM), sodium pyruvate (400 mM), penicillin (100 IU/mL), and streptomycin (0.1 mg/mL) in normoxic conditions (5% CO_2_). HT-29 cells were cultured in McCoys modified medium supplemented with 10% fetal calf serum, L-glutamine (200 mM), sodium pyruvate (400 mM), penicillin (100 IU/mL), and streptomycin (0.1 mg/mL) in normoxic conditions (5% CO_2_).

### 2.4 Cell-death analysis

The cell-death measurements were carried out using a Fluostar Omega fluorescence plate reader (BMG Labtech, Ortenberg, Germany) with temperature and atmosphere-controlled settings, as previously reported (Grootjans et al., 2016). In brief, the cells were seeded in duplicate at 10,000 cells per well in a 96-well adherent plate and incubated overnight at 37°C with 5% CO_2_. The next day, the cells were pretreated for 30 min with 1/10 dilution series of the indicated compounds ranging from 1 μM to 0.01 nM in the presence of 2.5 μM Sytox Green (Invitrogen, Waltham, MA, United States; no. S7020) and 50 µM zVAD.fmk. All the compound stock solutions were prepared in DMSO at 200 µM concentration. After stimulating the cells with human TNF at 20 ng/mL the plate was transferred to the incubator. Sytox Green intensity was measured in function of time at 0, 14 and 24 h with an excitation filter of 485 nm, an emission filter of 520 nm. The cell death was calculated by subtracting the background fluorescence from the induced Sytox Green fluorescence and by dividing the obtained result by the maximal fluorescence (minus the background fluorescence) obtained by permeabilization of the cells by using Triton X-100 at a final concentration of 0.1%. Curves were plotted in GraphPad 9 for the IC_50_ calculation.

### 2.5 KinaseProfiler™ assay (eurofins cerep)

RIPK1 (h) is incubated with 8 mM MOPS pH 7.0, 0.2 mM EDTA, 0.33 mg/mL myelin basic protein, 10 mM Magnesium acetate and [γ-^33^P-ATP] (specific activity and concentration as required). The reaction is initiated by the addition of the Mg/ATP mix. After incubation for 120 min at room temperature, the reaction is stopped by the addition of phosphoric acid to a concentration of 0.5%. An aliquot of the stopped reaction is spotted onto a filter and washed four times for 4 min in 0.425% phosphoric acid and once in methanol prior to drying and scintillation counting.

### 2.6 Immunoblotting

MEFs were seeded in 6-well plates at a density of 300.000 cells/well in 2 mL. The next day, the cells were suspended in 1 mL of medium and pre-treated for 30 min with the indicated compounds at 1/5 dilutions series for PERK ranging from 5 to 0.04 µM and 1 μg/mL of Tm followed by 4 h treatment with 20 ng/mL of hTNF. For RIPK1, the cells were pre-treated at 1/10 dilutions series ranging from 10 to 0.01 nM, 50 µM zVAD.fmk and 1 µM TAKi before stimulation with 20 ng/mL of hTNF for 2.5 h. Then, the cells were washed two times with 1 mL of ice-cold PBS before lysis in 0.2 mL of Laemmli buffer 1x (10% (w/v) SDS, 50% glycerol, 0.5% bromophenol blue, 250 mM Tris–HCl pH 6.8) and 250 mM of dithiothreitol (DTT). The cell lysate was then collected and boiled at 95 °C for 10min and stored at −20°C. Proteins expression was later monitored by immunoblotting.

### 2.7 Molecular docking

Molecular docking was carried out using Glide (Chen et al., 2018). The input structures for PERK and RIPK1 were extracted from the Protein Data Bank (PDB ID: 4M7I and 4NEU, respectively) (Saur et al., 2021). Missing loops were modelled using MODELLER v10.2 (Fiser and Sali, 2003), and the structures were prepared using the Protein Preparation Wizard in Maestro (Madhavi Sastry et al., 2013). Ligands were prepared using the LigPrep module of Maestro, assuming a physiological pH of 7.4. Docking in Glide was performed with the OPLS2005 force field using the extra precision protocol. The binding site was defined based on the inhibitors present in the respective crystal structures. Docking was initially done unconstrained, but due to unrealistic poses being generated for PERK, core constraints were added to the adenylyl mimetic to ensure kinase-inhibitor-like docking poses by constraining its distance to the hinge region. Otherwise, default settings were kept.

## 3 Results

### 3.1 Compounds design

According to the design strategy mentioned above, a series of 17 novel analogues presenting *para*-substituted pyridinyl (**18–29**) and aryl (**30–34**) groups were designed and synthesised. Different halogens together with small alkyls such as methyl and trifluoromethyl were introduced in both pyridinyl (**18–22**) and aryl (**30–34**) analogues. Specifically for the pyridinyl compounds, additional small groups were added with different polarity (**23** with -O-CH_3_, **26** with -OH and the 4-pyridinyl analogue **27**) and these were compared with compounds **24**, **28** and **29** featuring bulkier moieties (5-cyclopropyl, 5-benzyl and 5-phenyl). The molecules were then tested for their selectivity profile against PERK and RIPK1 by Western blot. Afterwards, their cell death inhibitory activity was quantified by fluorometric cell death assay and finally compound UAMC-3861 (**22**) was selected for further biological characterisation (Grootjans et al., 2016).

### 3.2 Chemistry

**FIGURE 3:**
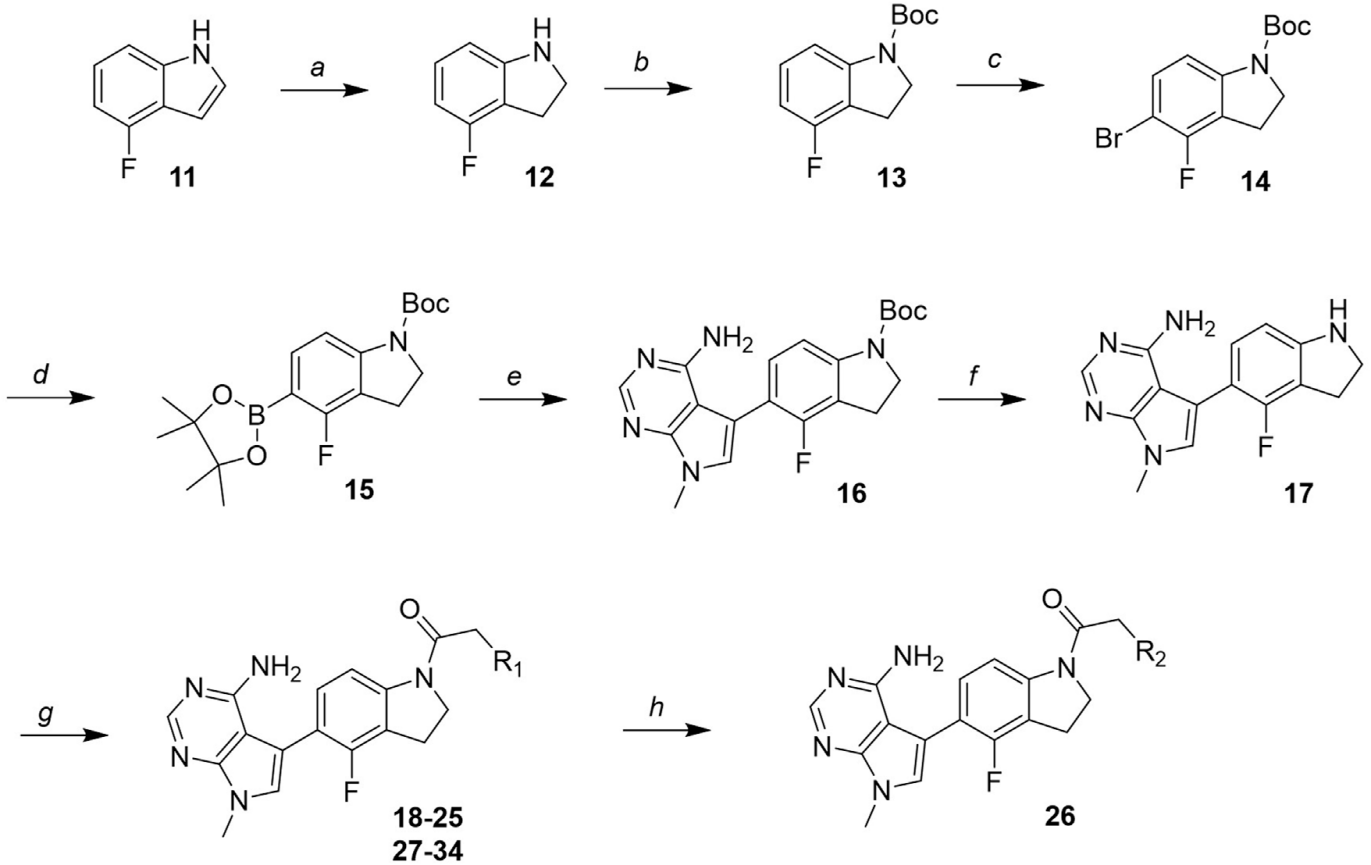
Synthesis of the common intermediate **17** based on GSK’157 synthetic route followed by amide coupling to obtain novel RIPK1 inhibitors **18–34**. Reagents and conditions: (a) 4-fluoro-1*H*-indole, NaBH_3_CN, AcOH, 0°C, 10 min, rt, 1 h; (b) Boc_2_O, DMAP, DCM, rt, 48 h; (c) NBS, DCM, rt, 1 h; (d) bis(pinacolato) diboron, KOAc, 1,4-dioxane, water, 80°C for 16 h (e) (t-Bu)_3_PHBF_4_, Pd_2_ (dba)_3_, 90°C, 2 h; (f) HCl in 1,4-dioxane 4N, 1,4-dioxane, rt, 16 h; (g) HATU, corresponding carboxylic acid, DIPEA, DMF, rt, 16 h; (h) **23,** BBr_3_, DCM, 0°C, 48 h.

**FIGURE 4:**
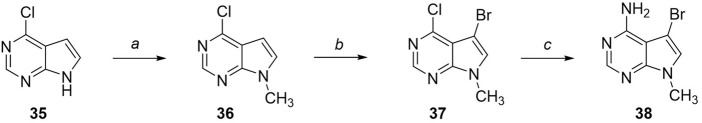
Synthesis of the coupling reagent 5-Bromo-7-methyl-7*H*-pyrrolo [2,3-*d*]pyrimidin-4-amine. Reagents and conditions: (a) 4-chloro-7*H-*pyrrolo [2,3-*d*]pyrimidine, Cs_2_CO_3_, CH_3_I, DMF, 1 h, rt; (b) NBS, DCM, 0°C, 1 h, rt, 3 h; (c) NH_3_ aq 30%, 120°C, 48 h.

The novel library was synthesised following the synthetic route reported for GSK’414 and GSK’157 (Axten et al., 2012; 2013). The common intermediate **17** was synthesised from the commercially available 4-fluoroindole as depicted in Figure 3. After the reduction of the 4-fluoroindole **11** to the corresponding indoline **12**, the secondary amine group was Boc-protected (**13**) before allylic bromination with the *N*-bromosuccinimide (**14**). Finally, the reaction with the bis(pinacolato) diboron led to the corresponding boronic ester intermediate that was then used in a Suzuki−Miyaura coupling reaction with 5-Bromo-7-methyl-7*H*-pyrrolo [2,3-*d*]pyrimidin-4-amine (**38**) synthesised as reported in Figure 4. Briefly, the conditions for the methylation of the 4-chloro-7*H*-pyrrolo [2,3-*d*]pyrimidine (**35**) NH and the following bromination were modified compared to the route of Axten M. J. *et al.* As reported in the experimental section (Axten et al., 2013). After the Suzuki-Miyaura coupling, the intermediate **16** was deprotected under acidic conditions and the free indoline was coupled with different aryl acetic acids to obtain the desired final compounds (**18–25** and **27–34**). The corresponding acetic acids were either commercially available or synthesised as reported in the experimental section. To obtain the hydroxy **26**, the methoxy group of compound **23** was demethylated in presence of borontribromide (BBr_3_). The corresponding R_1-2_ groups are reported in Table 1.

**TABLE 1:** Structures of the novel synthesised analogues and their corresponding IC_50_.

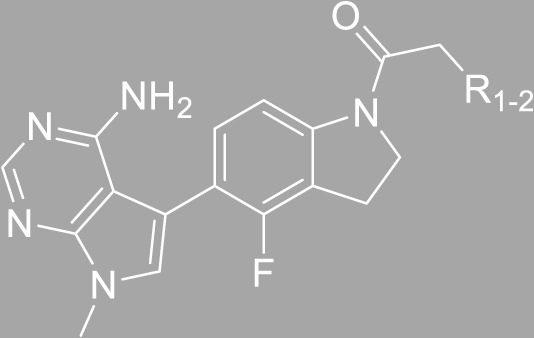					
Name	R_1-2_	cLogP^a^	PERK inhibition (nM)^b^	RIPK1 inhibition (nM)^c^	IC_50_ necroptosis (nM)^d^
**10**	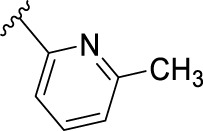	2.57	200–1000	0.1–1	0.42
**GSK’157**					
**18**	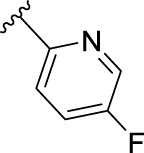	2.58	> 5000	≥ 10	13.5
**UAMC-4006**					
**19**	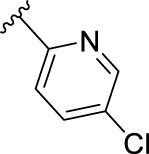	3.04	> 5000	1–10	2.5
**UAMC-4005**					
**20**	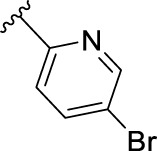	3.21	> 5000	1–10	3.2
**UAMC-4004**					
**21**	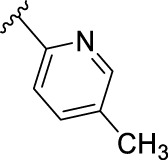	2.95	1000–5000	≥ 10	37.6
**UAMC-3715**					
**22**	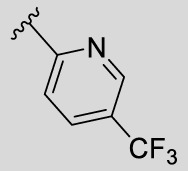	3.32	> 5000	1–10	2.9
**UAMC-3861**					
**23**	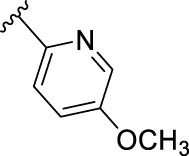	2.28	≥ 5000	≥ 10	39
**UAMC-4111**					
**24**	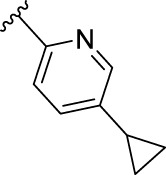	3.22	> 5000	≥ 10	33.5
**UAMC-4235**					
**25**	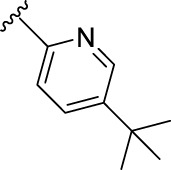	3.98	> 5000	≥ 10	40
**UAMC-3863**					
**26**	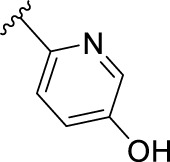	2.14	≥ 5000	≥ 10	2526
**UAMC-4112**					
**27**	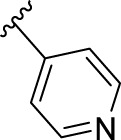	2.05	> 5000	≥ 10	3025
**UAMC-4217**					
**28**	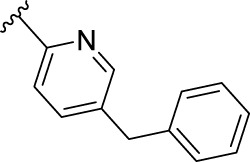	4.53	≥ 5000	≥ 10	62
**UAMC-3862**					
**29**	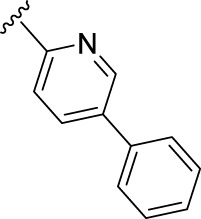	4.09	> 5000	≥ 10	n.a.
**UAMC-4233**					
**30**	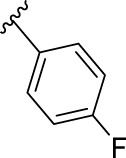	3.41	1000–5000	0.1–1	0.01
**UAMC-4114**					
**31**	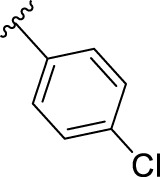	3.88	1000–5000	0.1–1	0.4
**UAMC-4116**					
**32**	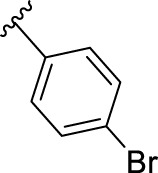	4.04	1000–5000	1–10	0.3
**UAMC-4113**					
**33**	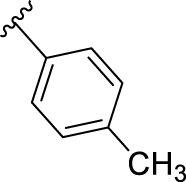	3.78	1000–5000	≥ 10	n.a.
**UAMC-4115**					
**34**	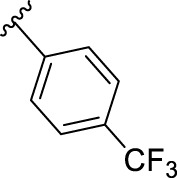	4.15	≥ 5000	1–10	1.1
**UAMC-4003**					

n.a. = not active, IC_50_ >10 µM.

^a^ LogP calculated with Collaborative Drug Discovery Inc. CDD Vault® software.

^b^ IC_50_ estimated from a Western blot expressed in nM. Immortalized MEFs were pretreated for 30 min with increasing concentrations of GSK’157 and the indicated compounds and stimulated with tunicamycin (Tm) (1 μg/ml) for 4 h.

^c^ IC_50_ estimated from a Western blot expressed in nM. Immortalised MEFs were pretreated for 30 min with zVAD.fmk (50 μM), TAK1i (1 μM) and the indicated compounds and then stimulated for 2.5 h with hTNF (20 ng/ml).

^d^ IC_50_ values are calculated from measurements at least in duplicate. RIPK1 kinase-dependent necroptosis was induced in immortalized MEFs by 30 min pretreatment with pan-caspases inhibitor zVAD-fmk (50 μM) and increasing concentrations of the indicated compounds then stimulated by hTNF (20 ng/ml). The TNF-mediated necroptosis was measured at 24 h post-stimulation by Sytox Green positivity. Analogues **30–33** retain activity for PERK at the higher concentration 1000–5000 nM.

### 3.3 Biological characterisation

The compounds were first tested for their abilities to inhibit PERK and RIPK1 enzymatic activities in mouse embryonic fibroblasts (MEFs). To do so, we monitored, by immunoblot, the effects of the compounds on the autophosphorylation of PERK and RIPK1.

**FIGURE 5:**
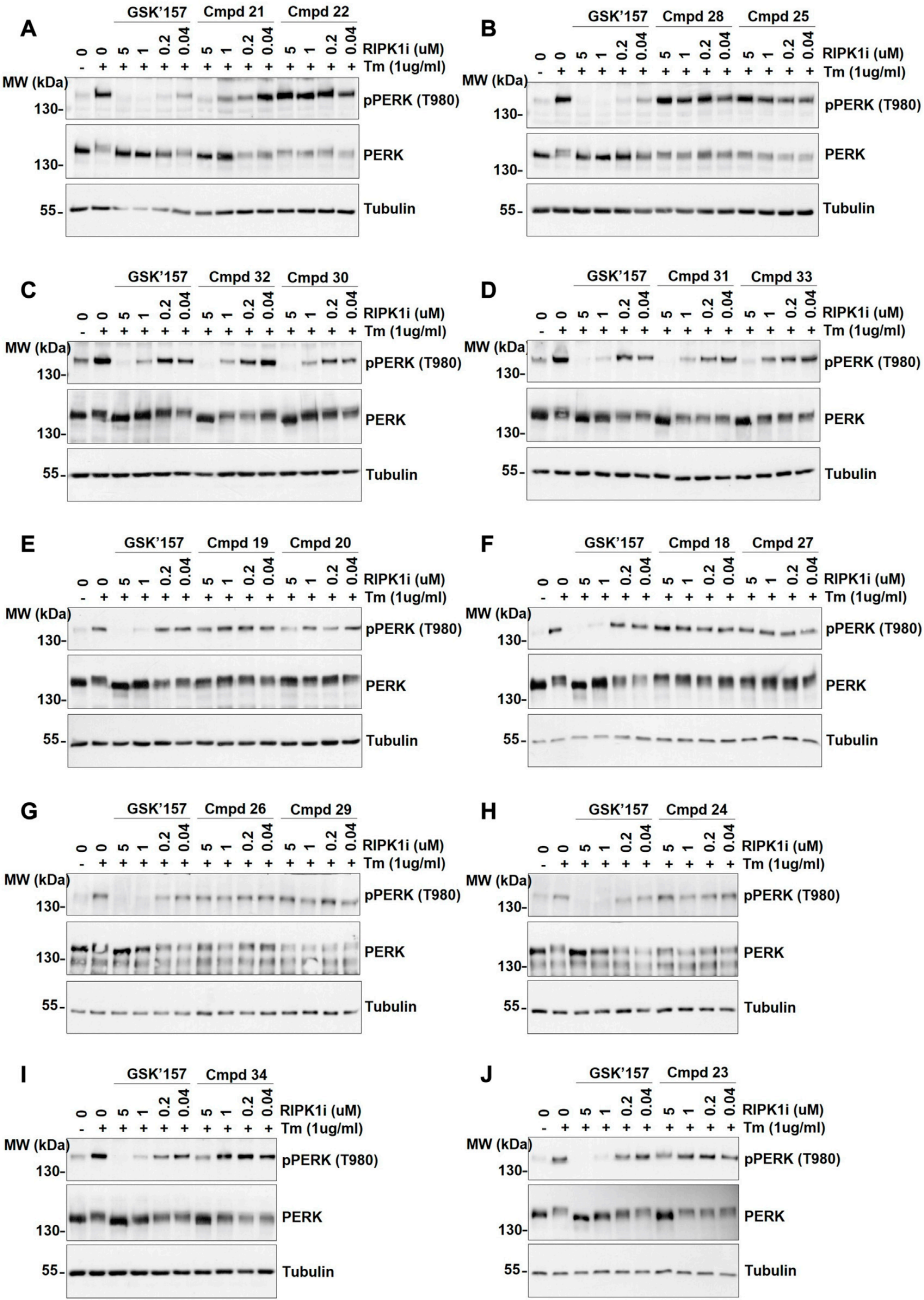
The effect of GSK’157 in comparison with compounds (cmpd) **18–34** on PERK autophosphorylation is reported in figures **(A–J)**. Immortalized MEFs were pretreated for 30 min with increasing concentrations of GSK’157 and the indicated compounds and stimulated with tunicamycin (Tm) (1 μg/mL) for 4 h. The cell lysates were then immunoblotted as indicated.

**FIGURE 6:**
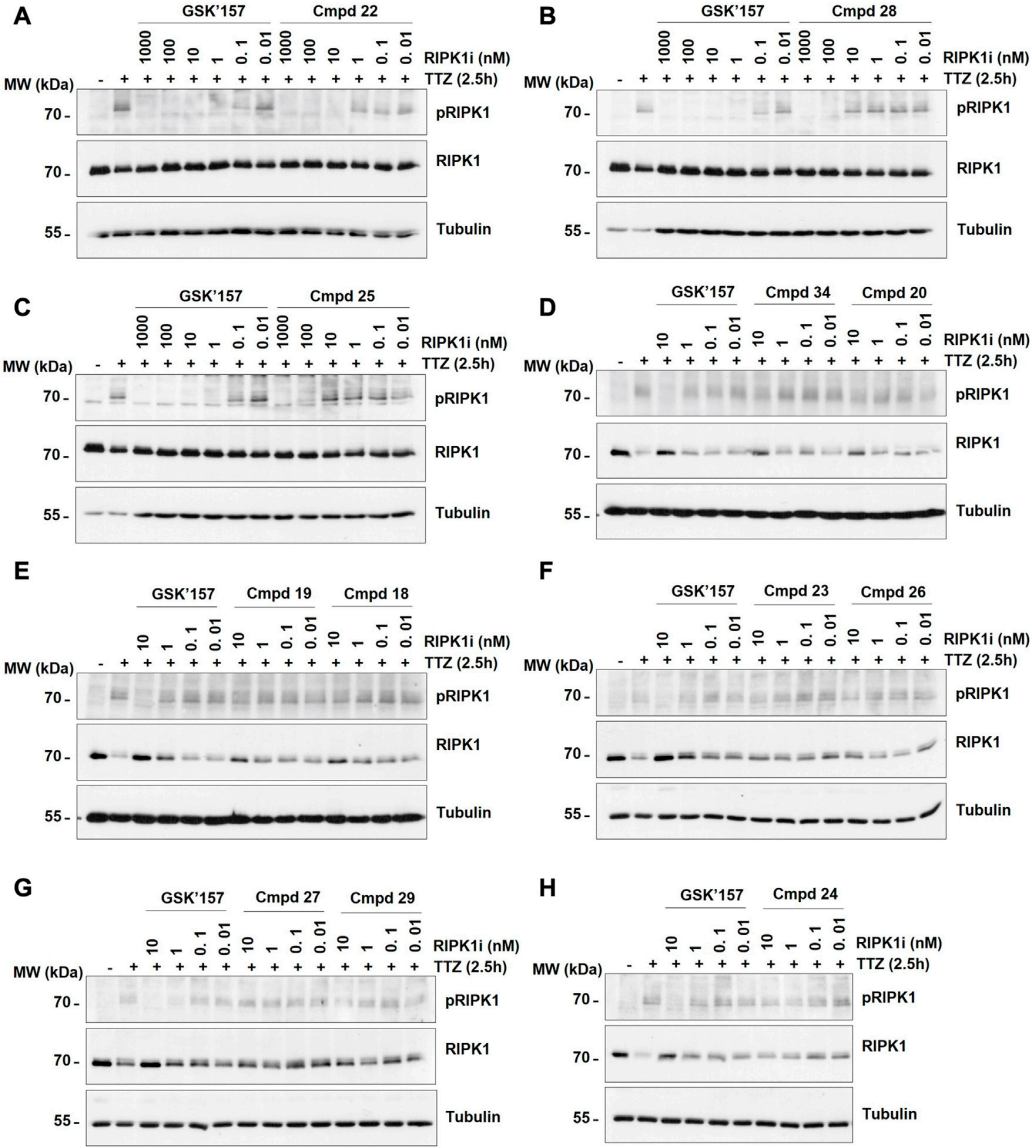
The effect of GSK’157 in comparison with compounds (cmpd) **18–20**, **22–29** and **34** (not-active on PERK) on RIPK1 autophosphorylation (pS166) is reported in figures **(A–H)**. Immortalized MEFs were pretreated for 30 min with zVAD.fmk (50 μM), TAK1i (1 μM) and the indicated compounds and then stimulated for 2.5 h with h TNF (20 ng/mL). TTZ = hTNF + TAK1i + zVAD.fmk. The cell lysates were then immunoblotted as indicated.

Activation of PERK was induced by stimulation of the cells with Tunicamycin, as previsouly reported (Bull and Thiede, 2012). More precisely, the immortalised MEFs were pre-incubated for 30 min with the analogues **18–34** at different concentrations (0.04, 0.2, 1, and 5 µM) prior to 4 h stimulation with Tunicamycin (1 μg/mL). As shown in Figures 5A–J, 12 out of the 17 compounds did not inhibit PERK at the tested concentrations while GSK’157 inhibited the autophosphorylation of PERK in a concentration range of 200–1000 nM. Their activities were evaluated by comparing the intensity of the signal to unstimulated MEFs (considered as zero phosphorylation) and Tunicamycin stimulated cells (considered as 100% PERK phosphorylation). The 12 compounds (**18–20, 22–29** and **34**) that had lost their inhibitory capacity on PERK were then tested for their ability to inhibit RIPK1 as shown in Figures 6A–H. Activation and autophosphorylation of RIPK1 was induced by stimulating the cells with TNF in the presence of TAK1 inhibitor and of the pan-caspases inhibitor zVAD.fmk, a well established necroptotic trigger (Vanlangenakker et al., 2011). The MEFs were pre-incubated for 30 min with the different compounds (0.01 nM–1000 nM) and then stimulated with human TNF (20 ng/mL) for 2.5 h. Similar to the PERK assessment, IC_50_ values for RIPK1 inhibition were estimated as a range of concentrations, and are reported in Table 1. These experiments provide an estimation of the specificity of our analogues against PERK and RIPK1 in comparison to the benchmark GSK’157 already reported as a potent PERK and RIPK1 inhibitor.

Consequently, the compounds were evaluated for their potential protection of RIPK1-mediated necroptosis following human TNF stimulation. The corresponding IC_50_ are reported in Table 1. The library was screened in MEFs cells due to their sensibility to TNF-induced necroptosis as described in the literature (Vanlangenakker et al., 2011). The IC_50_ values were calculated based on the cell response measured over 24 h by Sytox Green positivity.

## 4 Discussion

The series of novel compounds were screened for their activity to selectively inhibit RIPK1 kinase-dependent necroptosis (Table 1) and their inhibitory effect on PERK and RIPK1 autophosporylation (Figure 5A–J and 6A-H). The novel analogues were compared with the benchmark GSK’157 which was confirmed as one of the most potent RIPK1 inhibitors currently available (IC_50_ = 0.42 nM). The compounds with a *para*-substituted-pyridinyl moiety (**18–29**) showed negligible PERK inhibition in contrast to GSK’157. Similar results were observed for the *para*-phenyl series (**30–34**), but to a lesser extent. The results confirmed the detrimental role of the *para*-substituent for PERK potency. In contrast, these compounds retained or even improved RIPK1 inhibition compared to GSK’157.

The IC_50_ value for necroptosis inhibition of the analogues substituted with halogen (F, Cl and Br) resulted in potent compounds. Compound **18** with the fluorine (IC_50_ = 13.5 nM) was less potent than the corresponding analogues **19** (IC_50_ = 2.5 nM) and **20** (IC_50_ = 3.2 nM), featuring a chlorine and bromide, respectively. The methyl analogue **21** was less potent (IC_50_ = 37 nM), whereas the corresponding trifluoromethyl (**22**, IC_50_ = 2.9 nM) is similar in potency to chlorine (**19**) and bromine (**20**). The introduction of methoxy (**26**), cyclopropyl (**24**) and *tert*-butyl (**25**) resulted in compounds that were equipotent to the methyl analogue **21**. Polar substitutions such as the *para*-hydroxy (**26**) and a shift of the ring nitrogen of the pyridine ring (**27**) were detrimental for necroptosis inhibition (IC_50_ > 2 µM). This result confirms the high-hydrophobic nature of the RIPK1-allosteric pocket. Similarly, the insertion of bulky substituents such as benzyl and phenyl (**28** and **29**) negatively affects the activity of the analogues.

Furthermore, when a halogen was introduced on the corresponding *para* position of phenyl series, the activity increased notably with IC_50_ values <0.5 nM. Compound **30** with fluorine is the most potent compound of the entire series (IC_50_ = 0.01 nM), with 40-fold higher potency than GSK’157. To the best of our knowledge, this is the most potent necroptosis inhibitor ever reported (Martens et al., 2020; Chen et al., 2022; Shi et al., 2022). However, compared to the corresponding pyridinyl analogues (**18**–**20**), the phenyl analogues **30**–**32** retained some PERK inhibition, albeit at concentration higher than 1 µM which still results in excellent selectivity. Surprisingly, the introduction of a *para-*methyl substituent on the phenyl ring (**33**) resulted in a complete loss of necroptosis inhibition. The corresponding trifluoromethyl analogue (**34**, IC_50_ = 1.1 nM) was again a potent necroptosis inhibitor. This striking difference in potency between the methyl and trifluoromethyl analogue was also seen in the pyridinyl series (**21** and **22**), although less prominent.

Compound **22** (UAMC-3861) was preferably selected for further characterisation. We chose an analogue that showed no PERK inhibition at the highest concentration tested (5 µM) combined with a single-digit nanomolar necroptosis inhibition. Compounds **19** and **20** also meet these criteria. However, different studies suggested that the trifluoromethyl moiety improves the interaction with the hydrophobic allosteric pocket of RIPK1 (Liu and Gray, 2006; Harris et al., 2013).

**FIGURE 7:**
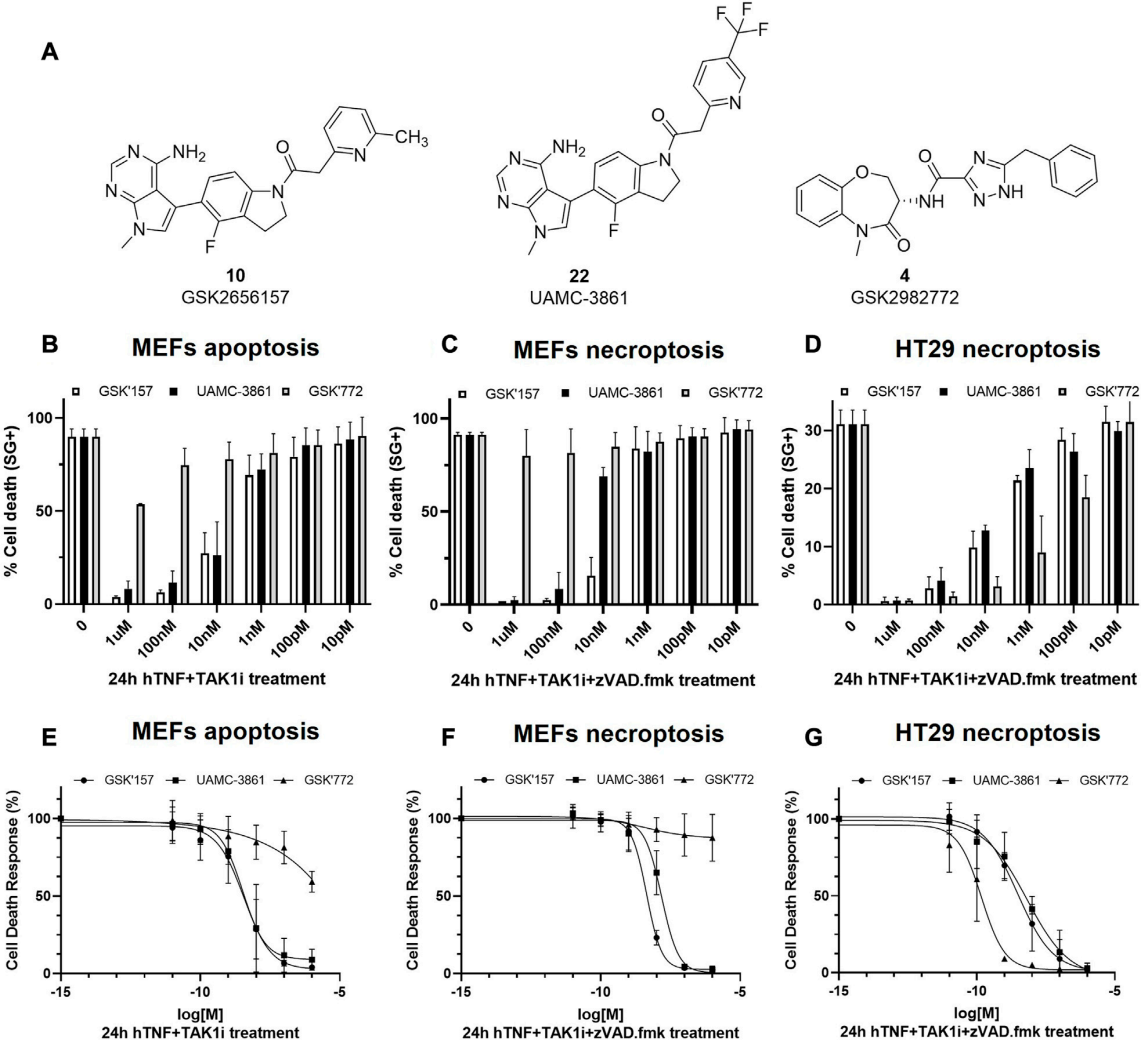
RIPK1-dependent cell death inhibition in mouse and human cells for GSK'157, UAMC-3861 and GSK'772. **(A)** Chemical structures of the compounds that were tested *in vitro* for RIPK1-dependent apoptosis and necroptosis. **(B**,**C)** MEFs and **(D)** HT-29 were pre-treated for 30 min with increasing concentrations of the indicated compounds. RIPK1 kinase-dependent apoptosis was induced by TAK1i + hTNF (100 pg/mL) and RIPK1 kinase-dependent necroptosis by zVAD.fmk + TAK1i + hTNF (100 pg/mL) and hTNF + zVAD.fmk (20 ng/mL). Cell death was measured over time by Sytox Green positivity (SG+) and the results are presented as mean ± SEM of three independent experiments. **(E–G)** MEFs and HT-29 were pre-treated for 30 min with increasing concentrations of the indicated compounds. In presence or absence of TAK1i (1 µM) and/or zVAD.

The selected compound **22** together with GSK’157 and the clinically most advanced RIPK1 inhibitor GSK’772, were tested for necroptosis and apoptosis inhibition in murine MEFs and human HT-29 cells (Figures 7A–G; Table 2). Moreover, the three compounds were tested for inhibition of hRIPK1 (Table 2).

**TABLE 2:** Overview of the IC_50_ values of compounds GSK'157, UAMC-3861 and GSK'772 in mouse and human cells.

	Apoptosis hTNF + TAK1i	Necroptosis hTNF + zVAD.fmk	Necroptosis hTNF + TAK1i + zVAD.fmk	Necroptosis hTNF + TAK1i + zVAD.fmk	hRIPK1 inhibition
	**MEFs**	**MEFs**	**MEFs**	**HT-29**	**-**
Name	IC_50_ (nM)^a^	IC_50_ (nM)^a^	IC_50_ (nM)^a^	IC_50_ (nM)^a^	IC_50_ (nM)^a^ ^,^ ^c^
**GSK’157**	3.7	0.7	4.4	3.1	53
**UAMC-3861**	3.3	1.06	15	6.5	139
**GSK’772**	n.a^b^	n.a^b^	n.a^b^	0.2	36

^a^ IC_50_ values are calculated from measurements at least in duplicate.

^b^ n. a. = not active, IC_50_ > 100 nM.

^c^ The IC_50_ values are determined based on 9 concentrations (0.0001 μM–1.0 μM) in presence of 10 μM ATP (Supporting Information).

It was previously reported that the binding of hTNF to TNFR1 can induce RIPK1 kinase-dependent apoptosis and necroptosis (Jin and El-Deiry, 2006; Vanlangenakker et al., 2011). In MEFs, TNF together with the pan caspases inhibitor zVAD.fmk induces RIPK1-dependent necroptosis while TNF together with TAK1 inhibitor induces RIPK1 kinase-dependent apoptosis. We also included the conditions selected for the Western blot experiments, where necroptosis was induced by the combination of hTNF, zVAD.fmk and TAK1 inhibitor.

In RIPK1 kinase-dependent necroptosis induced with hTNF + zVAD.fmk in mouse cells (MEFs) GSK’157 and UAMC-3861 showed similar potency. As expected, when necroptosis was induced with the stronger condition in MEFs cells (hTNF + TAKi + zVAD.fmk), the IC_50_ values decreased from 6-fold (GSK’157) to 14-fold (UAMC-3861). As previously reported by Harris P. A. *et al.*, GSK’772 is confirmed to be a species-specific inhibitor with no activity in mouse RIPK1-dependent necroptosis (Harris et al., 2017). To induce necroptosis in human colorectal adenocarcinoma cell line (HT-29) together with hTNF and the pan caspases inhibitor zVAD.fmk, TAK1 inhibitor is also needed. The high potency of the human necroptosis inhibitor GSK’772 (IC_50_ = 0.2 nM) is confirmed while GSK’157 (IC_50_ = 3.1 nM) and UAMC-3861 (IC_50_ = 6.5 nM) are less potent, but still in the same single-digit nanomolar range as observed in mouse cells. Only necroptosis conditions were tested in HT-29 since the cell line is not sensitive to RIPK1-dependent apoptosis as reported in the literature (Kong et al., 2018).

A similar trend in inhibitory potency for GSK772, GSK’157 and UAMC-3861 was also confirmed in the enzymatic assay against human RIPK1 (Table 2).

### 4.1 Docking study

**FIGURE 8:**
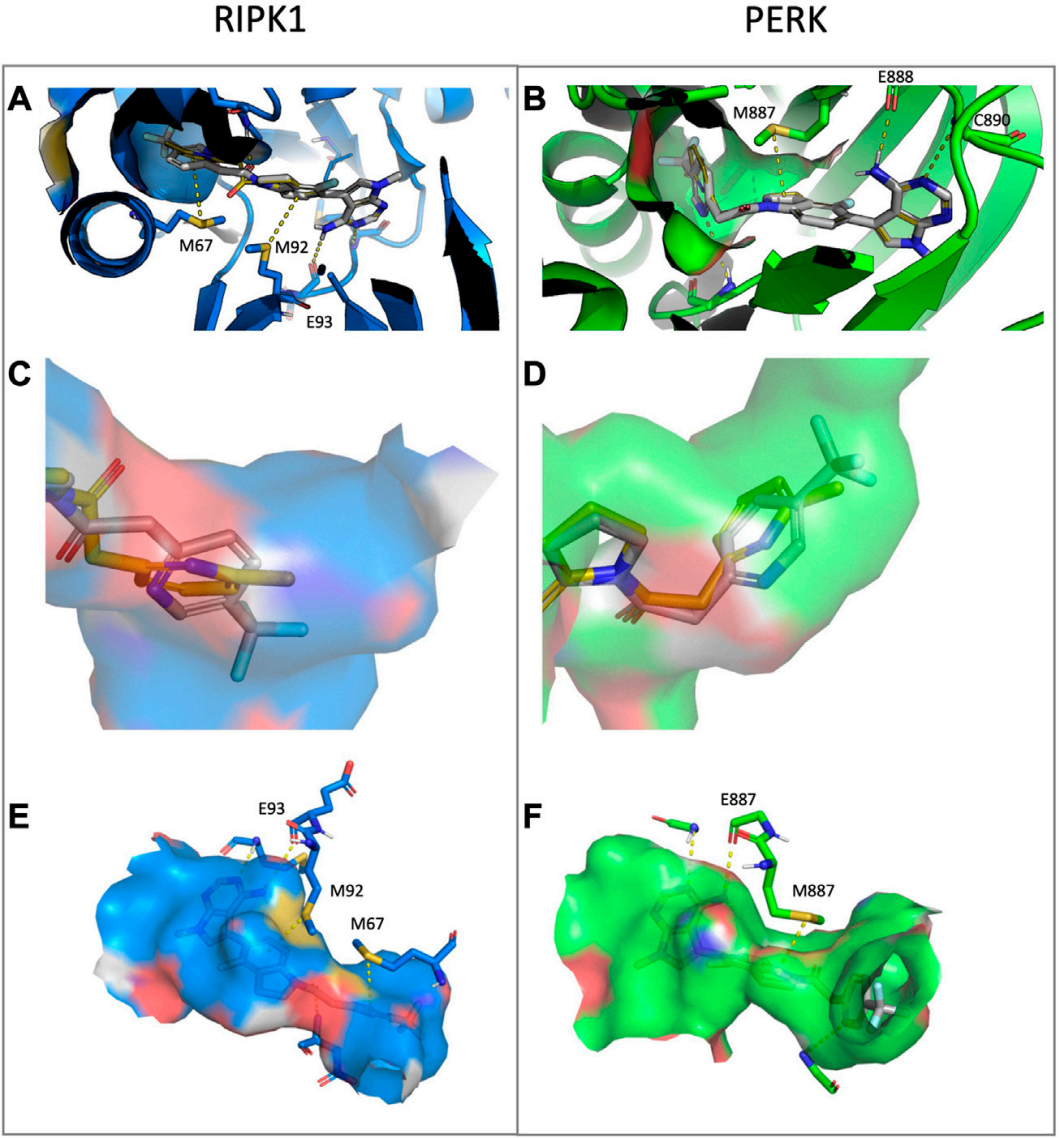
Docking of compound UAMC-3861 (grey sticks) and GSK’157 (yellow lines) in RIPK1 (blue) and PERK (green) **(A–B)**, representation of the allosteric pockets of RIPK1 and PERK with GSK’157 and UAMC-3861 **(C–D)** and surface representation of the binding pocket for RIPK1 (blue) and PERK (green) and the different interactions with UAMC-3861 **(E–F)**. **(A)** In RIPK1, UAMC-3861 is able to interacts with the gatekeeper Met92 and tend to form a methionine-aromatic interaction with 4-fluorindoline ring. **(B)** In PERK, the trifluoromethyl-pyridinyl substituent is surrounded by a less hydrophobic environment. The pyridinyl substituent forms a methionine-aromatic interaction with the gatekeeper Met887. **(C)** Zoom on the allosteric pocket of RIPK1 where the positions of the pyridinyl moieties of GSK’157 and UAMC-3861 are compared. **(D)** Zoom on the allosteric pocket of PERK where the different orientations of the pyridinyl rings are compared. **(E,F)** The trifluoromethyl-pyridinyl moiety occupied a different pocket in RIPK1 **(E)** and PERK **(F)**. While for the latter the binding site is slightly solvent-exposed, for RIPK1 the pocket is buried, featuring a more hydrophobic profile due to the presence of three Leu, two Val, and Met (not shown in detail).

UAMC-3861 was further studied for its mode of interaction with both RIPK1 and PERK in a computational study using molecular docking (Figures 8A–F). This was done in comparison with the binding mode of GSK’157. In RIPK1, UAMC-3861 is able to form an extra methionine interaction with the trifluoromethyl-pyridinyl substituent. Moreover, the amide group of UAMC-3861 interacted with the backbone amine of Asp156 while the gatekeeper Met92 and Met67 formed methionine-aromatic interactions with the 4-fluoroindoline ring, and the trifluoromethyl-pyridinyl substituent, respectively. In addition, the docking study also confirms the high hydrophobicity of the RIPK1 allosteric pocket, since the CF_3_ moiety is surrounded by Met66, Met67 (C-helix), Leu70, Val75 (loop following C-helix), Leu129, Val134 (catalytic loop), and Leu159 (after DLG) as seen in Figure 8A. In PERK (Figure 8B), a stabilisation of the pyridinyl substituent was detected through the interaction between the pyridinyl ring and the backbone amine of Phe162. In addition, a methionine-aromatic interaction between the gatekeeper Met887 and the pyridinyl substituent was observed. When we compared the different poses of the substituent, in RIPK1 the pyridinyl is situated between the C-helix, the DLG-motif, and the activation loop; for PERK it is between the C-helix and the beta-sheet domain. The different interactions for GSK’157 methyl-pyridinyl and the corresponding trifluoromethyl-pyridinyl of UAMC-3861 are depicted in Figure 8C (RIPK1) and 8D (PERK). In Figures 8E,F, the different nature of the two allosteric pockets is illustrated. The allosteric pocket of PERK (green) is partly solvent-exposed, differently from the more hydrophobic one of RIPK1 (blue), which is buried inside the protein. Superposition of the docking poses in RIPK1 onto PERK and *vice versa* shows that neither docking pose is compatible with the other protein. In particular, the UAMC-3861 docking pose is incompatible with PERK due to steric clashes with the DFG-motif which impede the pyridinyl substituent from binding to the analogous RIPK1 allosteric binding pocket. Instead, the substituent is forced into a different, more solvent-exposed pocket, which is more unfavourable for binding of hydrophobic moieties. Concludingly, this docking study confirmed that the *para*-substituted pyridine ring is favourable for binding to RIPK1 whereas it is detrimental for binding to PERK.

## 5 Conclusion

The role of TNF-mediated inflammation and cell death in different pathologies is widely recognised. Particularly, the TNF inflammatory cascade can induce inflammation and the release of cytokines through the activation of RIPK1-dependent apoptosis or necroptosis. The previously reported work in which GSK’157, a well-known PERK inhibitor, was revealed as a potent RIPK1 inhibitor opened the door to further optimisation of the chemical structure to enhance RIPK1 selectivity over PERK (Rojas-Rivera et al., 2017). Moreover, a kinome scan reported by Axten J.M. et al. For GSK’157 suggested a good selectivity profile of the compound, even though RIPK1 was not included in the panel. In this study we confirmed the hypothesis that the potency and selectivity for RIPK1 could be increased by derivatising the corresponding *para*-position of the GSK’157 pyridinyl ring (Axten et al., 2013; Chintha et al., 2019). In addition, based on the mode of interaction of GSK’157 this should result in a typical type II kinases inhibitor, a less explored class of RIPK1 inhibitors. We compared the potency of a *para*-substituted pyridinyl series (**18–29**) with the corresponding phenyl series (**30–34**). This demonstrates the importance of the pyridine ring to increase the selectivity for RIPK1 over PERK. Moreover, a smaller size of the *para*-substituent positively affected the potency for RIPK1. The hydrophobic nature of the RIPK1 allosteric pocket accommodating the *para*-substituted pyridine ring was confirmed, particularly considering the dramatic decrease of potency for the analogues having polar *para* substituents. To the best of our knowledge, the *para*-fluoro phenyl analogue (**30**) is the most potent necroptosis inhibitor described with an IC_50_ = 0.01 nM. However, since this compound retained some PERK inhibition at higher concentrations, we selected UAMC-3861 (**22**) for further evaluation in both murine and human cell lines. UAMC-3861 was confirmed as a highly potent compound with single digit nanomolar IC_50_ values for the inhibition of RIPK1-dependent necroptosis in both mouse and human cells as well as for the inhibition of RIPK1 dependent apoptosis in mouse cells. Compared with the clinical RIPK1 inhibitor GSK’772, UAMC-3861 is slightly less potent in human cells, but it has the advantage of potency in mouse cells in which GSK’772 is inactive. This suggests that UAMC-3861 will be an excellent tool compound to study RIPK1-dependent cell death in mouse models with the potential to translate to human cell lines.

## Data Availability

The original contributions presented in the study are included in the article/Supplementary Materials, further inquiries can be directed to the corresponding author.
