# Supplementary material for: From PERK to RIPK1: Design, synthesis and evaluation of novel potent and selective necroptosis inhibitors
Source: Front Chem. 2023 Apr 7;11:1160164. doi: 10.3389/fchem.2023.1160164 (PMC10119423; doi:10.3389/fchem.2023.1160164)
Supplement: Supplementary file 1 [file DataSheet1.pdf]

Supplementary Material

Contents

1. General procedure for the synthesis of compounds……………………………………..…S2-S15

2. Western blot figure of non selective compounds **21, 30-33**…………………………………...S15

3. HRMS spectra………………………………………………………………………….S16-S32

4. Dose-response curve for compounds **18-34**……………………..………………………..S33-S34

References…………………………………………………………………...……………S35

1 General procedure for the synthesis of compounds

Procedure for the synthesis of the common intermediate (4-fluoro-2,3-dihydro-1H-indol-5-yl)-7-methyl-7H-pyrrolo[2,3-d]pyrimidin-4-amine dihydrochloride (17) (Axten et al., 2013)**.**

*4-fluoro-2,3-dihydro-1H-indole (**12**).* A solution of 4-fluoroindole (1.423 g, 10.53 mmol) in acetic acid was cooled in an ice bath. Sodium cyanoborohydride (1.456 g, 23.17 mmol) was added portion-wise and the reaction was allowed to warm to room temperature and stir for 1h. The reaction mixture was cooled in an ice bath and neutralized with 50% wt/wt NaOH keeping the internal temperature <15 °C. Water was added (10 ml), and the mixture was extracted with ether (3x15 ml). The combined ether extracts were dried over Na_2SO_4, filtered, and concentrated. The residue was purified by silica flash column chromatography (EtOAc/heptane) to obtain the intermediate 4-fluoro-2,3-dihydro-1*H*-indole (1.094 g, 7.98 mmol, 76 % yield). ^{1}H NMR (400 MHz, DMSO-d_6), δ 2.93 (t, *J* = 8.61 Hz, 2H), 3.47 (dt, *J* = 1.91, 8.63 Hz, 2H), 6.28 (t, *J* = 8.64 Hz, 1H), 6.30 (d, *J* = 7.74 Hz, 1H), 6.90 (t, *J* = 7.97 Hz, 1H), 6.92 (d, *J* = 15.95 Hz, 1H); t_R 0.50 min. MS (ESI) *m/z* 138.1 $[M+H]^+$.

*tert-butyl 4-fluoroindoline-1-carboxylate (**13**).* A round-bottomed flask was charged with 4-fluoro-2,3-dihydro-1*H*-indole **12** (1.000 g, 7.310 mmol), di-*t*-butyldicarbonate (1.680 ml, 7.310 mmol), *N,N*-Di-iso-propylethylamine (2.550 ml, 14.630 mmol), 4-Dimethylaminopyridine (10.11 µl, 0.073 mmol) and DCM to give a yellow solution that was stirred for 2 days. The reaction mixture was diluted with 0.1 M HCl and extracted with DCM. The organic layer was dried over Na_2SO_4, filtered through a pad of silica gel, and concentrated to dryness to afford *tert*-butyl 4-fluoroindoline-1-carboxylate (1.490 g, 6.280 mmol, 86 % yield). ^{1}H NMR (400 MHz, DMSO-d_6), δ 1.50 (s, 9H), 3.07 (t, *J* = 8.40 Hz, 2H), 3.96 (t, *J* = 8.48 Hz, 2H), 6.76 (t, *J* = 8.52 Hz, 1H), 7.19 (q, *J* = 7.24 Hz, 1H), 7.48 (br. s, 1H); t_R 2.12 min. MS (ESI) *m/z* 182.2 $[M+H]^+$.

*tert-butyl 5-bromo-4-fluoroindoline-1-carboxylate (**14**).* *N*-bromosuccinimide (1.372 g, 7.71 mmol) was added to a stirring solution of 1,1-dimethylethyl 4-fluoro-2,3-dihydro-1*H*-indole-1-carboxylate **13** (1.463 g, 6.17 mmol) in DCM. The reaction was stirred at room temperature for 1h. Then, the reaction mixture was diluted with water (10 ml) and saturated aqueous Na_2CO_3 (10 ml). The mixture was extracted with DCM (3 x 10 ml), and the organic layer dried over Na_2SO_4, filtered through pad of silica gel, and concentrated to dryness to obtain *tert*-butyl 5-bromo-4-fluoroindoline-1-carboxylate (1.85 g, 6.18 mmol, 94 % yield). ^{1}H NMR (400 MHz, DMSO-d_6) δ 1.49 (s, 9H), 3.11 (t, *J* = 8.62 Hz, 2H), 3.97 (t, *J* = 8.73 Hz, 2H), 7.37 - 7.48 (m, 2H); t_R 2.27 min. MS (ESI) *m/z* 218.1 $[M\text{-}Boc+2H]^+$.

*1,1-dimethylethyl 4-fluoro-5-(4,4,5,5-tetramethyl-1,3,2-dioxaborolan-2-yl)-2,3-dihydro-1H-indole-1-carboxylate (**15**).* A round bottom flask was charged with 1,1-dimethylethyl 5-bromo-4-fluoro-2,3-dihydro-1*H*-indole-1-carboxylate **14** (1.850 g, 6.180 mmol), bis(pinacolato)diboron (2.350 g, 9.270 mmol), potassium acetate (1.820 g, 18.54 mmol) and 1,4-dioxane to give a yellow suspension. The mixture was stirred and purged with argon for 2 minutes. Then 1,1'Bis(diphenylphosphino)ferrocenepalladium(II)dichloride-DCM adduct (0.058 g, 0.071 mmol) was added to the reaction mixture, which was stirred at 80°C for 16 h. The cooled reaction mixture was diluted with water (5 ml) and saturated aqueous $NaHCO_3$ (5 ml), then extracted with EtOAc (3 x 15ml). The combined organic extracts were washed with water (15 ml), dried over Na_2SO_4, filtered through pad of silica gel and concentrated in vacuo. The residue was purified by silica flash column chromatography (EtOAc/Heptane) to give 1,1-dimethylethyl 4-fluoro-5-(4,4,5,5-tetramethyl-1,3,2-dioxaborolan-2-yl)-2,3-dihydro-1*H*-indole-1-carboxylate (1.482 g, 4.08 mmol, 66% yield). ^{1}H NMR (400 MHz, DMSO-d_6) δ 1.17 (s, 12H), 1.27 (s, 12H), 1.50 (s, 9H), 3.04 (t, J = 8.71 Hz, 2H), 3.97 (t, J = 8.74 Hz, 2H), 7.29 - 7.54 (m, 2H); t_R 2.35 min. MS (ESI) *m/z* 182.1 [M/2], 308.1 $[M\text{-}Boc+2H]^+$.

*1,1-dimethylethyl 5-(4-amino-7-methyl-7H-pyrrolo[2,3-d]pyrimidin-5-yl)-4-fluoro-2,3-dihydro-1H-indole-1-carboxylate (**16**).* A flask was charged with 5-bromo-7-methyl-7*H*-pyrrolo[2,3-*d*]pyrimidin-4-amine **38** (0.838 g, 3.690 mmol), *tert*-butyl 4-fluoro-5-(4,4,5,5-tetramethyl-1,3,2-dioxaborolan-2-yl)indoline-1-carboxylate **15** (1.470 g, 4.060 mmol), K_3PO_4 tribasic (1.560 g, 7.380 mmol), 1,4-dioxane and water to give a yellow suspension at room temperature under argon. The reaction was stirred and purged with argon for 5 minutes. Then, tri(*tert*-butylphosphonium)tetrafluoroborate (0.054 g, 0.185 mmol) and tris(dibenzylideneacetone)dipalladium(0) (0.085 g, 0.092 mmol) were added to the reaction mixture, which was heated to 90°C for 2 h. After cooling to room temperature, the precipitated solids were collected by filtration and washed with water (10 ml) and then with ether (15 ml). The solids were dissolved in DCM/MeOH 9:1. The solution was filtered through pad of Celite and concentrated to give 1,1-dimethylethyl 5-(4-amino-7-methyl-7H-pyrrolo[2,3-*d*]pyrimidin-5-yl)-4-fluoro-2,3-dihydro-1*H*-indole-1-carboxylate (0.970 g, 2.530 mmol, 69 % yield). ^{1}H NMR (400 MHz, DMSO-d_6), δ 1.51 (s, 9H), 3.10 – 3.18 (m, 2H), 3.73 (s, 3H), 4.01 (t, J = 8.6 Hz, 2H), 6.02 (br. s, 2H), 7.17 (t, J = 8.0 Hz, 1H), 7.23 (s, 1H), 7.57 (br. s, 1H), 8.13 (s, 1H); t_R 1.74 min. MS (ESI) *m/z* 384.3 $[M+H]^+$.

(4-fluoro-2,3-dihydro-1H-indol-5-yl)-7-methyl-7H-pyrrolo[2,3-d]pyrimidin-4-amine dihydrochloride (**17**). *Tert*-butyl 5-(4-amino-7-methyl-7*H*-pyrrolo[2,3-*d*]pyrimidin-5-yl)-4-fluoroindoline-1-

carboxylate **16** (0.940 g, 2.452 mmol) was suspended in 1,4-dioxane and HCl 4N in 1,4-dioxane (13.54 ml, 54.20 mmol) was added at room temperature. A compact brown solid quickly formed in the solution. The mixture was then stirred at room temperature overnight. Then the reaction was filtered and the collected solid was washed with ether (10 ml) and dried to afford (4-fluoro-2,3-dihydro-1*H*-indol-5-yl)-7-methyl-7*H*-pyrrolo[2,3-*d*]pyrimidin-4-amine dihydrochloride (0.924 g, 2.59 mmol, 94% yield). ^{1}H NMR (400 MHz, DMSO- d_6), δ 3.16 (t, *J* = 8.07 Hz, 2H), 3.70 (t, *J* = 8.10 Hz, 2H), 3.84 (s, 3H), 6.93 (d, *J* = 7.34 Hz, 1H), 7.21 (t, *J* = 7.64 Hz, 1H), 7.61 (s, 1H), 8.51 (s, 1H); t_R 1.09 min. MS (ESI) *m/z* 284.2 $[M+H]^+$.

Procedure for the synthesis of the coupling reagent 5- bromo-7-methyl-7H-pyrrolo[2,3-d]pyrimidin-4-amine (38) (Axten et al., 2012)**.**

*4-chloro-7-methyl-7H-pyrrolo[2,3-d]pyrimidine (**36**)* (Lumb J., Trevor et al., 2004)**.** In a round-bottom flask 4-chloro-7*H*-pyrrolo[2,3-*d*]pyrimidine (2.200 g, 14.33 mmol) was dissolved in DMF (30 ml). After cooling down to 0°C, iodomethane (1.784 ml, 28.70 mmol) was added dropwise, and the reaction mixture was allowed to stir at room temperature for 2 h. Then, the reaction was quenched with water (20 mL) and extracted with EtOAc (3 x 30 ml). The combined organic extracts were dried using anhydrous Na_2SO_4, and concentrated *in vacuo*. The residue was purified by silica flash column chromatography (EtOAc/heptane) to obtain 4-chloro-7-methyl-7*H*-pyrrolo[2,3-*d*]pyrimidine (1.980 g, 11.81 mmol, 82 % yield). ^{1}H NMR (400 MHz, DMSO-d_6) δ 3.85 (s, 4H), 6.64 (d, *J* = 3.5 Hz, 1H), 7.74 (d, *J* = 3.5 Hz, 1H), 8.64 (s, 1H); t_R 1.22 min. MS (ESI) *m/z* 168.1 $[M+H]^+$.

*4-chloro-7-methyl-7H-pyrrolo[2,3-d]pyrimidine (**37**).* To a stirring solution of 4-chloro-7-methyl-7*H*-pyrrolo[2,3-*d*]pyrimidine (1.817 g, 10.84 mmol) in DCM (16 mL) *N*-bromosuccinimide (2.412 g, 13.55 mmol) was added portion wisely at 0°C for 1 h and the reaction was stirred for 4 h at room temperature. The solvent was evaporated, and the solid was washed with water (3 x 15 ml) and dried to afford 5-bromo-4-chloro-7-methyl-7*H*-pyrrolo[2,3-*d*]pyrimidine (2.409 g, 9.77 mmol, 90 % yield). ^{1}H NMR (400 MHz, DMSO d_6) δ 3.82 (s, 4H), 7.99 (s, 1H), 8.67 (s, 1H).); t_R 1.60 min. MS (ESI) *m/z* 248.0, 249.9 $[M+H]^+$.

*5- bromo-7-methyl-7H-pyrrolo[2,3-d]pyrimidin-4-amine (**38**).* A suspension of 5-bromo-4-chloro-7-methyl-7*H*-pyrrolo[2,3-*d*]-pyrimidine (1.000 g, 4.060 mmol) in 30% aqueous ammonia (5 mL) was stirred for 2 days at 100 °C in a sealed vessel. The mixture was allowed to cool to room temperature

and filtered. The collected solid was washed with Et_2O (2 x 10 ml) and dried to afford 5-bromo-7-methyl-7*H*-pyrrolo[2,3-*d*]pyrimidin-4-amine (0.820 g, 3.610 mmol, 89 % yield) as a white solid. ^1H NMR (400 MHz, DMSO-d_6) δ 3.84 (s, 3H), 6.72 (br. s, 2H), 7.38 (s, 1H), 8.11 (s, 1H); t_R 0.27 min. MS (ESI) *m/z* 229.0, 230.1 $[M+H]^+$.

General procedure A for the synthesis of the corresponding pyridyl acetic acid (41 and 42) (Biscoe and Buchwald, 2009)**.**

A stirring solution of the corresponding 5-substituted-2-chloropyridine (1 eq) and 1,1-dimethylethyl acetate (2 eq) in toluene (3 ml) was purged with argon gas for 10 min, and then *t*-BuXPhos palladium(II) phenethylamine chloride (0.1 eq) was added. The reaction was cooled down to 0°C in an ice bath and LHMDS 1M in toluene (3 eq), was added dropwise over 1h. After the addition, the reaction was stirred at room temperature overnight. The reaction was quenched with saturated aqueous NH_4Cl (2 ml), extracted with EtOAc (3 x 10 ml), and the combined organic layers were washed with brine, dried over anhydrous Na_2SO_4 filtered and concentrated *in vacuo*. The residual oil was purified by silica flash column chromatography (EtOAc/Heptane) to yield the intermediate **39** and **40.**

Trifluoroaceticacid (10 eq) was added dropwise to a stirring solution of the corresponding acetate and triethylsilane (2.5 eq) in DCM at room temperature. After addition, the mixture was stirred at room temperature overnight. The reaction was concentrated *in vacuo* and diethyl ether was added (20 ml) to the residual oil, which caused an exothermic precipitation of the product. The mixture was allowed to cool down and the solid was isolated by filtration, washed with diethyl ether (3 x 5 ml) and dried to give the corresponding TFA salts **41** and **42.**

*5-(tert-butyl)-2-(carboxymethyl)pyridin-1-ium 2,2,2-trifluoroacetate (**41**).* General procedure A with *tert*-butyl 2-(5-(*tert*-butyl)pyridin-2-yl)acetate **39** (0.074 g, 0.297 mmol) to obtain 5-(*tert*-butyl)-2-(carboxymethyl)pyridin-1-ium 2,2,2-trifluoroacetate (0.042 g, 0.137 mmol, 46 %). ^1H NMR (400 MHz, DMSO-d_6) δ 1.33 (s, 9H), 2.62 (s, 2H), 7.69 (d, *J* = 8.37 Hz, 1H), 8.33 (q, *J* = 3.54 Hz, 1H), 8.65 (d, *J* = 2.09 Hz, 1H); t_R 0.43 min. MS (ESI) *m/z* 194.2 $[M+H]^+$.

*5-benzyl-2-(carboxymethyl)pyridin-1-ium 2,2,2-trifluoroacetate (**42**).* General procedure A with *tert*-butyl 2-(5-benzylpyridin-2-yl)acetate **40** (0.203 g, 0.716 mmol) to obtain 5-benzyl-2-(carboxymethyl)pyridin-1-ium 2,2,2-trifluoroacetate (0.172 g, 0.504 mmol, 70 % yield). ^1H NMR (400

MHz, DMSO-d_6) δ 3.95 (s, 2H), 4.07 (s, 2H), 7.20 - 7.35 (m, 5H), 7.65 (d, *J* = 8.12 Hz, 1H), 8.06 (dd, *J* = 2.08, 8.12 Hz, 1H), 8.69 (d, *J* = 1.78 Hz, 1H); t_R 1.15 min. MS (ESI) *m/z* 228.2 $[M+H]^+$.

General procedure B for the corresponding pyridyl acetic acid (44–46) (Spiekermann, 2019)**.**

To a mixture of the corresponding methyl-2-substituted pyridinyl acetate (1 eq) in THF (1.5 mL), water (1.5 mL) and MeOH (3 mL), lithium hydrate (3 eq) was added. The reaction mixture was stirred at 20 °C for 2 h. On completion, the reaction was concentrated *in vacuo* and the residue was acidified by 1.0 N HCl to pH = 3.0, washed with water (50 mL) and extracted with ethyl acetate (3 x 50 mL). The organic layer was dried over anhydrous Na_2SO_4, filtered and concentrated *in vacuo* to give the intermediates **44-46.**

*2-(5-cyclopropylpyridin-2-yl)acetic acid (**44**)* (Molander and Gormisky, 2008)**.** A stirring solution of cyclopropyltrifluoro-4-borane, potassium salt (0.354 g, 2.391 mmol), CataXium (0.023 g, 0.065 mmol), palladium(II)acetate (9.76 mg, 0.043 mmol) and cesium carbonate (2.124 g, 6.52 mmol) was flushed with argon. Then, methyl 2-(5-bromopyridin-2-yl)acetate (0.500 g, 2.173 mmol) was added and then suspended in 10 ml of a 9:1 mixture of toluene/water and heated to 100 °C for 24 h. Cooled to room temperature, and diluted with water (5 mL), the reaction mixture was extracted with DCM (3 × 5 mL). The organic layer was dried over anhydrous Na_2SO_4 filtered and concentrated *in vacuo*. The crude product was purified by silica gel column chromatography (Hep/EtOAc) to obtain methyl 2-(5-cyclopropylpyridin-2-yl)acetate **43** (0.185 g, 0.967 mmol, 45 % yield).

General procedure B with methyl 2-(5-cyclopropylpyridin-2-yl)acetate **43** (0.175 g, 0.915 mmol) to obtain 2-(5-cyclopropylpyridin-2-yl)acetic acid (0.162 g, 0.824 mmol, 90 % yield). ^{1}H NMR (400 MHz, DMSO-d_6) δ 0.89 (dt, *J* = 4.6, 7.0 Hz, 2H), 1.11 (dd, *J* = 3.7, 6.8 Hz, 2H), 2.14 (tt, *J* = 5.0, 8.3 Hz, 1H), 4.10 (s, 2H), 7.76 (d, *J* = 8.3 Hz, 1H), 8.05 (dd, *J* = 2.2, 8.2 Hz, 1H), 8.65 (d, *J* = 2.3 Hz, 1H); t_R 0.26 min. MS (ESI) *m/z* 178.1 $[M+H]^+$.

*2-(5-bromopyridin-2-yl)acetic acid (**45**).* General procedure B with methyl 2-(5-bromopyridin-2-yl)acetate (0.300 g, 1.304 mmol) to obtain 2-(5-bromopyridin-2-yl)acetic acid (0.232 g, 1.074 mmol, 82 % yield). ^{1}H NMR (400 MHz, DMSO-d_6) δ 3.74 (s, 2H), 7.35 (d, *J* = 8.3 Hz, 1H), 8.01 (dd, *J* = 2.5, 8.3 Hz, 1H), 8.61 (d, *J* = 2.6 Hz, 1H), 12.52 (s, 1H); t_R 1.12 min. MS (ESI) *m/z* 218.1, 219.1 $[M+H]^+$.

*2-(pyridin-4-yl)acetic acid (**46**)*. General procedure B with methyl 2-(pyridin-4-yl)acetate (0.500 g, 3.310 mmol) to obtain 2-(pyridin-4-yl)acetic acid (0.140 g, 1.021 mmol, 31 % yield). ^{1}H NMR (400 MHz, D_2O) δ 3.79 (d, *J* = 2.4 Hz, 2H), 7.81 (d, *J* = 6.2 Hz, 2H), 8.59 (d, *J* = 7.3 Hz, 2H); t_R 0.29 min. MS (ESI) *m/z* 138.0 $[M+H]^+$.

2-(5-phenylpyridin-2-yl)acetic acid (47) (Deng et al., 2011)**.**

A mixture of phenylboronic acid (0.423 g, 3.470 mmol), 2-(5-bromopyridin-2-yl)acetic acid (0.500 g, 2.314 mmol), tetrakis(triphenylphosphine)palladium(0) (0.535 g, 0.463 mmol) and potassium carbonate (0.960 g, 6.940 mmol) was suspended in 10 ml of a 9:1 mixture of 1,4-dioxane/water and heated to 100 °C for 1 h. Then the palladium residues were filtered and the filtrate was diluted with 30 ml of methanol and purified by reversed phase flash chromatography (water/MeOH) to obtain 2-(5-phenylpyridin-2-yl)acetic acid **47** (0.430 g, 2.017 mmol, 87 % yield). ^{1}H NMR (400 MHz, DMSO-d_6) δ 3.39 (s, 2H), 7.34 – 7.41 (m, 2H), 7.47 (dd, *J* = 6.9, 8.4 Hz, 2H), 7.67 (d, *J* = 8.2 Hz, 2H), 7.87 (dd, *J* = 2.5, 8.1 Hz, 1H), 8.65 (d, *J* = 2.4 Hz, 1H); t_R 1.48 min. MS (ESI) *m/z* 214.1 $[M+H]^+$.

General procedure C for the synthesis of final compounds (18–34) (Axten et al., 2013)**.**

A solution of 5-(2,3-dihydro-1*H*-indol-5-yl)-7-methyl-7*H*-pyrrolo[2,3-*d*]pyrimidin-4-amine dihydrochloride (1 eq), the corresponding 3-pyridinylacetic acid or 3-arylacetic acid substituent (1.1 eq), HATU (1 eq), and DIPEA (5 eq) in DMF (3ml) was stirred at room temperature overnight. Water (2 ml) was poured into the mixture and stirred for 30 minutes. The resulting suspension was filtered and then the collected solid was washed with water (3 x 3ml) and then dried to afford the desired product **18–22, 25, 28, 31** and **32** or purified by reversed phase flash chromatography (water/ACN) to obtain compounds **23, 24, 26, 27, 29, 30, 33** and **34.**

1-(5-(4-amino-7-methyl-7H-pyrrolo[2,3-d]pyrimidin-5-yl)-4-fluoroindolin-1-yl)-2-(5-fluoropyridin-2-yl)ethan-1-one (18) General procedure C with 5-(2,3-dihydro-1*H*-indol-5-yl)-7-methyl-7*H*-pyrrolo[2,3-*d*]pyrimidin-4-amine dihydrochloride **17** (0.100 g, 0.280 mmol) and 2-(5-bromopyridin-2-yl)acetic acid (0.600 g, 0.281 mmol) to obtain 1-(5-(4-amino-7-methyl-7*H*-pyrrolo[2,3-*d*]pyrimidin-5-yl)-4-fluoroindolin-1-yl)-2-(5-fluoropyridin-2-yl)ethan-1-one (0.038 g, 0.090 mmol, 64 % yield). ^{1}H NMR (400 MHz, DMSO-d_6) δ 3.17 – 3.28 (m, 2H), 3.73 (s, 3H), 4.07 (s, 2H), 4.32 (t, *J* = 8.7 Hz, 2H), 6.05 (br.s, 2H), 7.12 – 7.34 (m, 2H), 7.38 – 7.57 (m, 1H), 7.72 (dt, *J* = 9.4, 18.3, Hz, 1H), 7.90 (d, *J* = 8.3 Hz, 1H), 8.14 (s, 1H), 8.50 (s, 1H). ^{13}C NMR (101 MHz, DMSO-d_6) δ 24.45, 31.24, 44.48, 49.06,

101.22, 107.50, 112.63 (d, *J* = 2.3 Hz), 117.50 (d, *J* = 14.7 Hz), 119.34 (d, *J* = 22.8 Hz), 124.04 (d, *J* = 20.6 Hz), 125.74 (d, *J* = 2.2 Hz), 126.19 (d, *J* = 4.4 Hz), 131.34 (d, *J* = 1.8 Hz), 137.26 (d, *J* = 23.3 Hz), 144.94 (d, *J* = 8.0 Hz), 150.60, 151.99, 152.34 (d, *J* = 3.6 Hz), 156.03 (d, *J* = 243.1 Hz), 157.42, 157.55, 168.88; HRMS (ESI) *m/z* $[M+H]^+$ calcd for $C_{22}H_{18}F_2N_6O$ 421.1583, found 421.1595.

1-(5-(4-amino-7-methyl-7H-pyrrolo[2,3-d]pyrimidin-5-yl)-4-fluoroindolin-1-yl)-2-(5-chloropyridin-2-yl)ethan-1-one ***(19)*** General procedure C with 5-(2,3-dihydro-1*H*-indol-5-yl)-7-methyl-7*H*-pyrrolo[2,3-*d*]pyrimidin-4-amine dihydrochloride **17** (0.100 g, 0.280 mmol) and 2-(5-chlorolpyridin-2-yl)acetic acid (0.050 g, 0.309 mmol) to obtain 1-(5-(4-amino-7-methyl-7*H*-pyrrolo[2,3-*d*]pyrimidin-5-yl)-4-fluoroindolin-1-yl)-2-(5-chloropyridin-2-yl)ethan-1-one (0.078 g, 0.179 mmol, 64 % yield). ^{1}H NMR (400 MHz, DMSO-d_6) δ 3.24 (t, *J* = 8.5 Hz, 2H), 3.73 (s, 3H), 4.08 (s, 2H), 4.32 (t, *J* = 8.4 Hz, 2H), 6.03 (br. s, 2H), 7.19 (t, *J* = 8.0 Hz, 1H), 7.26 (s, 1H), 7.44 (d, *J* = 8.4 Hz, 1H), 7.86 – 7.96 (m, 2H), 8.13 (s, 1H), 8.57 (d, *J* = 2.5 Hz, 1H); ^{13}C NMR (101 MHz, DMSO-d_6) δ 24.44, 31.24, 44.60, 49.05, 101.21, 107.45, 112.62 (d, *J* = 3.2 Hz), 117.48 (d, *J* = 9.7 Hz), 119.38 (d, *J* = 21.6 Hz), 122.26, 125.73, 126.33, 129.91, 131.36 (d, *J* = 2.4 Hz), 136.82, 144.87 (d, *J* = 9.3 Hz), 147.94, 150.58, 152.10, 154.81, 156.17 (d, *J* = 294.9 Hz),168.69; HRMS (ESI) *m/z* $[M+H]^+$ calcd for $C_{22}H_{18}ClFN_6O$ 437.1287, found 437.1270

1-(5-(4-amino-7-methyl-7H-pyrrolo[2,3-d]pyrimidin-5-yl)-4-fluoroindolin-1-yl)-2-(5-bromopyridin-2-yl)ethan-1-one ***(20)*** General procedure C with 5-(2,3-dihydro-1*H*-indol-5-yl)-7-methyl-7*H*-pyrrolo[2,3-*d*]pyrimidin-4-amine dihydrochloride **17** (0.050 g, 0.140 mmol) and 2-(5-fluoropyridin-2-yl)acetic acid **45** (0.020 g, 0.140 mmol) to obtain 1-(5-(4-amino-7-methyl-7*H*-pyrrolo[2,3-*d*]pyrimidin-5-yl)-4-fluoroindolin-1-yl)-2-(5-bromopyridin-2-yl)ethan-1-one (0.086 g, 0.179 mmol, 64 % yield). ^{1}H NMR (400 MHz, DMSO-d_6) δ 3.24 (t, *J* = 8.4 Hz, 2H), 3.74 (s, 3H), 4.06 (s, 2H), 4.32 (t, *J* = 8.4 Hz, 2H), 6.13 (br.s, 2H), 7.19 (t, *J* = 8.1 Hz, 1H), 7.28 (s, 1H), 7.38 (d, *J* = 8.3 Hz, 1H), 7.90 (d, *J* = 8.2 Hz, 1H), 8.04 (dd, *J* = 2.5, 8.3 Hz, 1H), 8.15 (s, 1H), 8.65 (d, *J* = 2.5 Hz, 1H).^{13}C NMR (101 MHz, DMSO-d6) δ 24.44, 31.47, 44.67, 49.06, 96.03, 100.85, 108.18, 112.72 (d, *J* = 2.9 Hz). 116.95 (d, *J* = 14.8 Hz), 118.98, 119.59 (d, *J* = 23.7 Hz), 126.56, 126.84, 131.32 (d, *J* = 2.5 Hz), 139.61, 145.10 (d, *J* = 9.5 Hz), 149.88, 150.13, 155.26 (d, *J* = 242.3 Hz), 156.05, 168.64; HRMS (ESI) *m/z* $[M+H]^+$ calcd for $C_{22}H_{18}BrFN_6O$ 481.0782, found 481.0778.

1-(5-(4-amino-7-methyl-7H-pyrrolo[2,3-d]pyrimidin-5-yl)-4-fluoroindolin-1-yl)-2-(5-methylpyridin-2-yl)ethan-1-one ***(21)*** General procedure C with 5-(2,3-dihydro-1*H*-indol-5-yl)-7-methyl-7*H*-

pyrrolo[2,3-*d*]pyrimidin-4-amine dihydrochloride **17** (0.1 g, 0.28 mmol) and 2-(5-Methylpyridin-2-yl)acetic acid (0.040 g, 0.281 mmol) to obtain 1-(5-(4-amino-7-methyl-7*H*-pyrrolo[2,3-*d*]pyrimidin-5-yl)-4-fluoroindolin-1-yl)-2-(5-methylpyridin-2-yl)ethan-1-one (0.085g, 0.204 mmol, 72.7%). ^{1}H-NMR (400 MHz, $CDCl_3$) δ 2.31 (3H, s), 3.25 (2H, t, *J* = 8.28 Hz), 3.82 (3H, s), 3.98 (2H, s), 4.32 (2H, t, *J* = 8.35 Hz), 5.21 (2H, s), 6.97 (1H, s), 7.22 (1H, t, *J* = 7.91 Hz), 7.29 (1H, d, *J* = 7.87 Hz), 7.48 (1H, d, *J* = 6.69 Hz), 8.08 (1H, d, *J* = 8.14 Hz), 8.31 (1H, s), 8.37 (1H, s); ^{13}C NMR (101 MHz, $CDCl_3$) δ 18.24, 24.64, 31.40, 45.80, 49.27, 101.77, 107.89, 113.32 (d, *J* = 3.74 Hz), 117.59 (d, *J* = 14.57 Hz), 118.74 (d, *J* = 22.94 Hz), 123.54, 125.39, 131.27, 131.83, 137.56, 144.79 (d, *J* = 7.55 Hz), 149.98, 150.80, 151.83, 151.87, 156.07 (d, *J* = 244.76 Hz), 156.95, 168.87; HRMS (ESI) *m/z* $[M+H]^+$ calcd for $C_{23}H_{21}FN_6O$ 417.1834, found 417.1819.

*1-(5-(4-amino-7-methyl-7H-pyrrolo[2,3-d]pyrimidin-5-yl)-4-fluoroindolin-1-yl)-2-(5-(trifluoromethyl)pyridin-2-yl)ethan-1-one (**22**)* General procedure C with 5-(2,3-dihydro-1*H*-indol-5-yl)-7-methyl-7*H*-pyrrolo[2,3-*d*]pyrimidin-4-amine dihydrochloride **17** (0.074 g, 0.210 mmol) and 2-(5-(trifluoromethyl)pyridin-2-yl)acetic acid (0.470 g, 0.229 mmol) to obtain 1-(5-(4-amino-7-methyl-7*H*-pyrrolo[2,3-*d*]pyrimidin-5-yl)-4-fluoroindolin-1-yl)-2-(5-(trifluoromethyl)pyridin-2-yl)ethan-1-one (0.041 g, 0.086 mmol, 41 %). ^{1}H-NMR (400 MHz, DMSO-d_6) δ 3.26 (t, *J* = 8.44 Hz, 2H), 3.73 (s, 3H), 4.22 (s, 2H), 4.34 (t, *J* = 8.44 Hz, 2H), 6.04 (s, 2H), 7.19 (t, *J* = 8.08 Hz, 1H), 7.26 (s, 1H), 7.64 (d, *J* = 8.24 Hz, 1H), 7.90 (d, *J* = 8.20 Hz, 1H), 8.14 (s, 1H), 8.21 (dd, *J* = 1.98, 8.22 Hz, 1H), 8.92 (q, *J* = 0.76 Hz, 1H).;^{13}C NMR (101 MHz, DMSO-d_6) δ 24.03, 30.82, 44.79, 48.63, 100.76, 107.02, 112.25 (d, *J* = 10.2 Hz), 117.19 (d, *J* = 14.2 Hz) 118.86, 118.98 (d, *J* = 24.4 Hz), 124.91, 125.11 (d, *J* = 40.2 Hz), 130.94, 133.92 (d, *J* = 5.0 Hz), 144.40 (d, *J* = 8.5 Hz), 145.78, 150.17, 151.61, 155.60 (d, *J* = 242.4 Hz), 157.15, 160.27, 161.18, 167.98; HRMS (ESI) *m/z* $[M+H]^+$ calcd for $C_{23}H_{18}F_4N_6O$ 471.1551 found 471.1534.

*1-(5-(4-amino-7-methyl-7H-pyrrolo[2,3-d]pyrimidin-5-yl)-4-fluoroindolin-1-yl)-2-(5-methoxypyridin-2-yl)ethan-1-one (**23**)* General procedure C with 5-(2,3-dihydro-1*H*-indol-5-yl)-7-methyl-7*H*-pyrrolo[2,3-*d*]pyrimidin-4-amine dihydrochloride l **17** (0.19 g, 0.53 mmol) and 2-(pyridin-4-yl)acetic acid (0.09 g, 0.58 mmol) to obtain 1-(5-(4-amino-7-methyl-7*H*-pyrrolo[2,3-*d*]pyrimidin-5-yl)-4-fluoroindolin-1-yl)-2-(5-methoxypyridin-2-yl)ethan-1-one (0.180 g, 0.416 mmol, 78 % yield). ^{1}H NMR (400 MHz, DMSO-d_6) δ 3.23 (t, *J* = 8.4 Hz, 2H), 3.74 (s, 3H), 3.82 (s, 3H), 3.98 (s, 2H), 4.31 (t, *J* = 8.5 Hz, 2H), 6.02 (s, 2H), 7.19 (t, *J* = 8.0 Hz, 1H), 7.26 (s, 1H), 7.31 (d, *J* = 8.6 Hz, 1H), 7.39 (dd, *J* = 3.0, 8.6 Hz, 1H), 7.92 (d, *J* = 8.2 Hz, 1H), 8.14 (s, 1H), 8.22 (d, *J* = 2.9 Hz, 1H); ^{13}C NMR

(101 MHz, DMSO- d_6) δ 24.45, 31.22, 44.55, 49.09, 56.01, 101.23, 107.48, 112.61 – 112.63 (m), 117.44 (d, *J* = 14.9 Hz), 119.29 (d, *J* = 23.0 Hz), 121.70, 124.99, 125.67, 131.32 (d, *J* = 2.6 Hz), 145.03 (d, *J* = 8.1 Hz), 147.72, 150.63, 152.11, 154.65, 156.02 (d, *J* = 243.0 Hz). 157.63, 169.30; HRMS (ESI) *m/z* $[M+H]^+$ calcd for $C_{23}H_{21}FN_6O_2$ 433.1783, found 433.1773.

*1-(5-(4-amino-7-methyl-7H-pyrrolo[2,3-d]pyrimidin-5-yl)-4-fluoroindolin-1-yl)-2-(5-cyclopropylpyridin-2-yl)ethan-1-one (**24**).* General procedure C with 5-(4-fluoroindolin-5-yl)-7-methyl-7*H*-pyrrolo[2,3-*d*]pyrimidin-4-amine dihydrochloride **17** (0.100 g, 0.281 mmol) and 2-(5-cyclopropylpyridin-2-yl)acetic acid **44** (0.060 g, 0.337 mmol), to obtain 1-(5-(4-amino-7-methyl-7*H*-pyrrolo[2,3-*d*]pyrimidin-5-yl)-4-fluoroindolin-1-yl)-2-(5-cyclopropylpyridin-2-yl)ethan-1-one (0.028 g, 0.063 mmol, 23 % yield). ^{1}H NMR (400 MHz, DMSO-d_6) δ 0.64 – 0.77 (m, 2H), 0.93 – 1.04 (m, 2H), 1.93 (ddd, *J* = 5.2, 8.7, 13.5 Hz, 1H), 3.22 (t, *J* = 8.4 Hz, 2H), 3.73 (s, 3H), 3.98 (s, 2H), 4.31 (t, *J* = 8.4 Hz, 2H), 6.04 (s, 2H), 7.12 – 7.30 (m, 3H), 7.39 (dd, *J* = 2.5, 8.1 Hz, 1H), 7.90 (d, *J* = 8.2 Hz, 1H), 8.13 (s, 1H), 8.33 (d, *J* = 2.4 Hz, 1H); ^{13}C NMR (101 MHz, DMSO-d_6) δ 9.64, 12.83, 24.44, 31.23, 45.07, 49.13, 101.22, 107.51, 112.49 – 112.78 (m), 116.24, 117.43 (d, *J* = 15.0 Hz), 124.20, 125.71 (t, *J* = 2.1 Hz), 127.40 (d, *J* = 7.4 Hz), 131.31 (t, *J* = 2.7 Hz), 133.17, 137.70, 147.56, 150.60, 152.03, 152.77, 157.57, 169.15; HRMS (ESI) *m/z* $[M+H]^+$ calcd for $C_{25}H_{23}FN_6O$ 443.1990, found 443.1982.

*1-(5-(4-amino-7-methyl-7H-pyrrolo[2,3-d]pyrimidin-5-yl)-4-fluoroindolin-1-yl)-2-(5-(tert-butyl)pyridin-2-yl)ethan-1-one (**25**)* General procedure C with 5-(2,3-dihydro-1*H*-indol-5-yl)-7-methyl-7*H*-pyrrolo[2,3-*d*]pyrimidin-4-amine dihydrochloride **17** (0.040 g, 0.112 mmol) and 2-(5-(*tert*-butyl)pyridin-2-yl)acetic acid **41** (0.038 g, 0.124 mmol) to obtain 1-(5-(4-amino-7-methyl-7*H*-pyrrolo[2,3-*d*]pyrimidin-5-yl)-4-fluoroindolin-1-yl)-2-(5-(*tert*-butyl)pyridin-2-yl)ethan-1-one (0.012 g, 0.025 mmol, 23 % yield). ^{1}H NMR (400 MHz, $CDCl_3$) δ 1.33 (s, 9H), 3.27 (t, *J* = 8.42 Hz, 2H), 3.83 (s, 3H), 3.99 (s, 2H), 4.36 (t, *J* = 8.48 Hz, 2H), 5.15 (br.s, 2H), 6.97 (s, 1H), 7.23 (t, *J* = 7.99 Hz, 1H), 7.33 (d, *J* = 8.20 Hz, 1H), 7.68 (dd, *J* = 2.44, 8.16 Hz, 1H), 8.09 (d, *J* = 8.28 Hz, 1H), 8.32 (s, 1H), 8.59 (d, *J* = 2.08 Hz, 1H); ^{13}C NMR (101 MHz, $CDCl_3$) δ 24.64, 31.10, 31.56, 33.58, 45.64, 49.33, 101.48, 108.62, 113.47 (d, J=2.99 Hz), 117.00 (d, *J* = 13.74 Hz), 118.92 (d, *J* = 23.08 Hz), 123.42, 125.90 (d, *J* = 1.94 Hz), 131.17, 134.21, 144.67, 145.06, 147.07, 149.65, 150.24, 151.68, 154.80, 155.89, 168.91; HRMS (ESI) *m/z* $[M+H]^+$ calcd for $C_{26}H_{27}FN_6O$ 459.2303, found 459.2306.

*1-(5-(4-amino-7-methyl-7H-pyrrolo[2,3-d]pyrimidin-5-yl)-4-fluoroindolin-1-yl)-2-(5-hydroxypyridin-2-yl)ethan-1-one (**26**)* To a solution of **23** (0.100 g, 0.231 mmol) in anhydrous DCM (5 ml) was added borontribromide 17 % DCM solution (0.694 ml, 0.694 mmol) dropwise at 0 °C under nitrogen atmosphere. The reaction was stirred for 3 days at 0°C. The cooled mixture was diluted with water and saturated aqueous $NaHCO_3$, (10 ml) extracted with EtOAc (3 x 15 ml) and the combined organic extracts were dried over Na_2SO_4. The residue was purified by silica flash column chromatography (10% MeOH in DCM) to obtain 1-(5-(4-amino-7-methyl-7*H*-pyrrolo[2,3-*d*]pyrimidin-5-yl)-4-fluoroindolin-1-yl)-2-(5-hydroxypyridin-2-yl)ethan-1-one (0.035 g, 0.084 mmol, 36 % yield) ^{1}H NMR (400 MHz, DMSO-d_6) δ 3.22 (t, *J* = 8.5 Hz, 2H), 3.74 (s, 3H), 3.91 (s, 2H), 4.30 (t, *J* = 8.5 Hz, 2H), 6.02 (s, 2H), 7.09 – 7.22 (m, 3H), 7.26 (s, 1H), 7.92 (d, *J* = 8.2 Hz, 1H), 8.05 (d, *J* = 2.7 Hz, 1H), 8.14 (s, 1H), 9.79 (s, 1H); ^{13}C NMR (101 MHz, DMSO-d_6) δ 24.44, 31.22, 44.64, 49.07, , 101.23, 107.50, 112.62 (d, *J* = 8.1 Hz), 117.41 (d, *J* = 14.7 Hz), 119.29 (d, *J* = 23.1 Hz), 123.26, 124.94, 125.68, 131.32 (d, *J* = 2.8 Hz), 137.49, 145.06 (d, *J* = 8.0 Hz), 146.08, 150.63, 152.10, 152.79, 157.23, 157.63, 169.45,; HRMS (ESI) *m/z* $[M+H]^+$ calcd for $C_{22}H_{19}FN_6O_2$ 419.1626, found 419.1627.

*1-(5-(4-amino-7-methyl-7H-pyrrolo[2,3-d]pyrimidin-5-yl)-4-fluoroindolin-1-yl)-2-(pyridin-4-yl)ethan-1-one (**27**)* General procedure C with 5-(2,3-dihydro-1*H*-indol-5-yl)-7-methyl-7*H*-pyrrolo[2,3-*d*]pyrimidin-4-amine dihydrochloride **17** (0.100 g, 0.280 mmol) and 2-(pyridin-4-yl)acetic acid **46** (0.040 g, 0.280 mmol) to obtain 1-(5-(4-amino-7-methyl-7*H*-pyrrolo[2,3-*d*]pyrimidin-5-yl)-4-fluoroindolin-1-yl)-2-(pyridin-4-yl)ethan-1-one (0.078 g, 0.194 mmol, 69 % yield). ^{1}H NMR (400 MHz, DMSO-d_6) δ 3.18 – 3.30 (m, 2H), 3.73 (s, 3H), 3.95 (s, 2H), 4.19 – 4.38 (m, 2H), 6.03 (s, 2H), 7.12 – 7.38 (m, 4H), 7.91 (d, *J* = 8.1 Hz, 1H), 8.14 (s, 1H), 8.47 – 8.58 (m, 2H); ^{13}C NMR (101 MHz, DMSO d_6) δ 24.47, 31.23, 41.61, 48.95, 101.21, 107.41, 112.62 (d, *J* = 3.5 Hz) 117.58 (d, *J* = 15.1 Hz), 119.33 (d, *J* = 22.9 Hz), 125.73, 131.37 (d, *J* = 3.5 Hz), 144.43, 144.89 (d, *J* = 8.2 Hz), 149.87, 150.62, 152.11, 154.81, 156.02 (d, *J* = 243.1 Hz), 168.62; HRMS (ESI) *m/z* $[M+H]^+$ calcd for $C_{22}H_{19}FN_6O$ 403.1677, found 403.1658.

*1-(5-(4-amino-7-methyl-7H-pyrrolo[2,3-d]pyrimidin-5-yl)-4-fluoroindolin-1-yl)-2-(5-benzylpyridin-2-yl)ethan-1-one (**28**)* General procedure C with 5-(2,3-dihydro-1*H*-indol-5-yl)-7-methyl-7*H*-pyrrolo[2,3-*d*]pyrimidin-4-amine dihydrochloride **17** (0.107 g, 0.300 mmol) and 2-(5-benzylpyridin-2-yl)acetic acid **42** (0.075 g, 0.221 mmol) to obtain 1-(5-(4-amino-7-methyl-7*H*-pyrrolo[2,3-*d*]pyrimidin-5-yl)-4-fluoroindolin-1-yl)-2-(5-benzylpyridin-2-yl)ethan-1-one (0.098 g, 0.200 mmol, 67 %). ^{1}H NMR (400 MHz, $CDCl_3$) δ 3.26 (t, *J* = 8.36 Hz, 2H), 3.86 (s, 3H), 3.96 (s, 2H), 3.99 (s, 2H),

4.34 (t, *J* = 8.46 Hz, 2H), 6.09 (br.s, 2H), 7.04 (s, 1H), 7.16 – 7.23 (m, 4H), 7.27 – 7.32 (m, 2H),7.48 (dd, *J* = 2.16, 7.99 Hz, 1H), 8.07 (d, *J* = 8.22 Hz, 1H), 8.26 (s, 1H), 8.42 (d, *J* = 1.47 Hz, 1H); ^{13}C NMR (101 MHz, $CDCl_3$) δ 24.61, 31.73, 38.82, 45.66, 49.32, 101.19, 109.42, 113.56 (d, *J* = 3.23 Hz), 116.36 (d, *J* = 12.91 Hz), 119.07 (d, *J* = 24.29 Hz), 123.97, 126.48, 126.66, 128.82, 128.98, 131.08, 135.33, 137.47, 139.72, 145.38 (d, *J* = 8.20 Hz), 147.30, 149.54, 149.74, 152.49, 154.79, 157.16, 168.83; HRMS (ESI) *m/z* $[M+H]^+$ calcd for $C_{29}H_{25}FN_6O$ 493.2147, found 493.2154.

*1-(5-(4-amino-7-methyl-7H-pyrrolo[2,3-d]pyrimidin-5-yl)-4-fluoroindolin-1-yl)-2-(5-phenylpyridin-2-yl)ethan-1-one (**29**).* General procedure C with 5-(4-fluoroindolin-5-yl)-7-methyl-7*H*-pyrrolo[2,3-*d*]pyrimidin-4-amine dihydrochloride **17** (0.100 g, 0.281 mmol) and 2-(5-phenylpyridin-2-yl)acetic acid **47** (0.072 g, 0.337 mmol) to obtain 1-(5-(4-amino-7-methyl-7*H*-pyrrolo[2,3-*d*]pyrimidin-5-yl)-4-fluoroindolin-1-yl)-2-(5-phenylpyridin-2-yl)ethan-1-one (0.055 g, 0.115 mmol, 41 % yield). ^{1}H NMR (400 MHz, DMSO-d_6) δ 3.25 (t, *J* = 8.4 Hz, 2H), 3.73 (s, 3H), 4.11 (s, 2H), 4.36 (t, *J* = 8.4 Hz, 2H), 6.04 (br. s, 2H), 7.19 (t, *J* = 8.0 Hz, 1H), 7.26 (s, 1H), 7.38 – 7.56 (m, 4H), 7.69 – 7.77 (m, 2H), 7.93 (d, *J* = 8.2 Hz, 1H), 8.07 (dd, *J* = 2.5, 8.1 Hz, 1H), 8.13 (s, 1H), 8.82 (d, *J* = 2.1 Hz, 1H); ^{13}C NMR (101 MHz, DMSO-d_6) δ 24.02, 30.79, 44.69, 48.73, 100.78, 107.03, 112.21 (d, *J* = 3.0 Hz), 117.08 (d, *J* = 15.0 Hz), 118.92 (d, *J* = 23.1 Hz), 124.36, 125.25, 126.81, 128.07, 129.17, 130.91 (d, *J* = 2.0 Hz), 133.77, 134.66, 136.99, 144.53 (d, *J* = 8.3 Hz), 147.07, 150.18, 151.67, 154.38, 155.23 (d, *J* = 244.6 Hz), 157.19, 168.55; HRMS (ESI) *m/z* $[M+H]^+$ calcd for $C_{28}H_{23}FN_6O$ 479.1990, found 479.1976.

*1-(5-(4-amino-7-methyl-7H-pyrrolo[2,3-d]pyrimidin-5-yl)-4-fluoroindolin-1-yl)-2-(4-fluorophenyl)ethan-1-one (**30**)* General procedure C with 5-(2,3-dihydro-1*H*-indol-5-yl)-7-methyl-7*H*-pyrrolo[2,3-*d*]pyrimidin-4-amine dihydrochloride **17** (0.06 g, 0.17 mmol) and 2-(4-fluorophenyl)acetic acid (0.060 g, 0.190 mmol) to obtain 1-(5-(4-amino-7-methyl-7*H*-pyrrolo[2,3-*d*]pyrimidin-5-yl)-4-fluoroindolin-1-yl)-2-(4-fluorophenyl)ethan-1-one (0.012 g, 0.029 mmol, 17 % yield). ^{1}H NMR (400 MHz, DMSO-d_6) δ 3.24 (t, *J* = 7.8 Hz, 2H), 3.73 (s, 3H), 3.88 (s, 2H), 4.29 (t, *J* = 8.5 Hz, 2H), 6.02 (s, 2H), 7.12 – 7.22 (m, 3H), 7.26 (s, 1H), 7.33 (t, *J* = 7.2 Hz, 2H), 7.92 (d, *J* = 8.3 Hz, 1H), 8.13 (s, 1H); ^{13}C NMR (101 MHz, DMSO-d_6) δ 24.47, 31.22, 41.49, 48.89, 101.22, 107.46, 112.60 (d, *J* = 3.7 Hz), 115.43 (d, *J* = 21.2 Hz), 117.44 (d, *J* = 14.3 Hz), 119.23 (d, *J* = 23.1 Hz), 125.68, 129.59, 131.34 (d, *J* = 2.6 Hz), 131.58 (d, *J* = 3.1 Hz), 131.98 (d, *J* = 8.0 Hz), 145.05 (d, *J* = 8.0 Hz), 150.61, 152.10, 156.02

(d, *J* = 242.5 Hz), 157.62, 160.37, 162.78, 169.69; HRMS (ESI) *m/z* $[M+H]^+$ calcd for $C_{23}H_{19}F_2N_5O$ 420.1630, found 420.1642.

*1-(5-(4-amino-7-methyl-7H-pyrrolo[2,3-d]pyrimidin-5-yl)-4-fluoroindolin-1-yl)-2-(4-chlorophenyl)ethan-1-one (**31**)* General procedure C with 5-(2,3-dihydro-1*H*-indol-5-yl)-7-methyl-7*H*-pyrrolo[2,3-*d*]pyrimidin-4-amine dihydrochloride **17** (0.060 g, 0.170 mmol) and 2-(4-chlorophenyl)acetic acid (0.030 g, 0.190 mmol) to obtain 1-(5-(4-amino-7-methyl-7*H*-pyrrolo[2,3-*d*]pyrimidin-5-yl)-4-fluoroindolin-1-yl)-2-(4-chlorophenyl)ethan-1-one (0.025 g, 0.057 mmol, 34 % yield). ^{1}H NMR (400 MHz, DMSO-d_6) δ 3.09 – 3.25 (m, 2H), 3.72 (s, 3H), 3.89 (s, 2H), 4.17 – 4.40 (m, 2H), 6.01 (br.s, 2H), 7.09 – 7.49 (m, 6H), 7.84 – 7.97 (m, 1H), 8.13 (s, 1H); ^{13}C NMR (101 MHz, DMSO-d_6) δ 24.47, 31.23, 41.64, 48.90, 101.22, 107.43, 112.60 (d, *J* = 2.9 Hz), 117.47 (d, *J* = 15.0 Hz), 119.26 (d, *J* = 23.1 Hz), 125.68, 128.65, 131.35 (d, *J* = 2.6 Hz), 131.76, 132.05, 134.50, 145.01 (d, *J* = 8.1 Hz), 150.61, 152.11, 156.01 (d, *J* = 242.8 Hz), 157.62, 169.44; HRMS (ESI) *m/z* $[M+H]^+$ calcd for $C_{23}H_{19}ClFN_5O$ 436.1335, found 436.1335.

*1-(5-(4-amino-7-methyl-7H-pyrrolo[2,3-d]pyrimidin-5-yl)-4-fluoroindolin-1-yl)-2-(4-bromophenyl)ethan-1-one (**32**)* General procedure C with 5-(2,3-dihydro-1*H*-indol-5-yl)-7-methyl-7*H*-pyrrolo[2,3-*d*]pyrimidin-4-amine dihydrochloride **17** (0.060 g, 0.170 mmol) and 2-(4-bromophenyl)acetic acid (0.040 g, 0.190 mmol) to obtain 1-(5-(4-amino-7-methyl-7*H*-pyrrolo[2,3-*d*]pyrimidin-5-yl)-4-fluoroindolin-1-yl)-2-(4-bromophenyl)ethan-1-one (0.180 g, 0.375 mmol, 22 % yield). ^{1}H NMR (400 MHz, DMSO-d_6) δ 3.24 (t, *J* = 8.5 Hz, 2H), 3.73 (s, 3H), 3.88 (s, 2H), 4.28 (t, *J* = 8.2 Hz, 2H), 6.01 (br. s, 2H), 7.33 – 7.09 (m, 4H), 7.53 (d, *J* = 7.9 Hz, 2H), 7.91 (d, *J* = 8.3 Hz, 1H), 8.14 (s, 1H); ^{13}C NMR (101 MHz, DMSO-d_6) δ 24.47, 31.25, 41.71, 48.90, 101.20, 107.49, 112.00 – 112.96 (m), 117.51, 119.15, 119.36 (d, *J* = 3.6 Hz), 120.26, 125.56 – 125.85 (m), 131.35 (d, *J* = 2.0 Hz), 131.57, 132.43, 134.91, 144.46, 151.25 (d, *J* = 140.8 Hz), 153.65 (d, *J* = 232.3 Hz), 157.51, 169.37; HRMS (ESI) *m/z* $[M+H]^+$ calcd for $C_{23}H_{19}BrFN_5O$ 480.0830, found 480.0852.

*1-(5-(4-amino-7-methyl-7H-pyrrolo[2,3-d]pyrimidin-5-yl)-4-fluoroindolin-1-yl)-2-(p-tolyl)ethan-1-one (**33**)* General procedure C with 5-(2,3-dihydro-1*H*-indol-5-yl)-7-methyl-7*H*-pyrrolo[2,3-*d*]pyrimidin-4-amine dihydrochloride **17** (0.06 g, 0.17 mmol) and 2-(p-tolyl)acetic acid (0.030 g, 0.190 mmol) to obtain 1-(5-(4-amino-7-methyl-7*H*-pyrrolo[2,3-*d*]pyrimidin-5-yl)-4-fluoroindolin-1-yl)-2-(p-tolyl)ethan-1-one (0.022g, 0.053 mmol, 31 % yield). ^{1}H NMR (400 MHz, $CDCl_3$) δ 2.34 (s, 3H), 3.24 (t, *J* = 8.5 Hz, 2H), 3.79 (s, 2H), 3.86 (s, 3H), 4.18 (t, *J* = 8.5 Hz, 2H), 5.98 (br. s, 2H), 7.04 (s, 1H), 7.12 – 7.26 (m, 5H), 8.12 (d, *J* = 8.4 Hz, 1H), 8.28 (s, 1H); ^{13}C NMR (101 MHz, $CDCl_3$) δ 20.90,

24.36, 31.37, 43.02, 48.65, 100.93, 108.93, 113.19 (d, *J* = 3.7 Hz), 116.10 (d, *J* = 14.7 Hz), 118.34 (d, *J* = 21.1 Hz), 125.99 (d, *J* = 2.7 Hz), 126.00, 128.70, 128.81, 129.38, 130.31, 136.72, 130.85 (d, *J* = 2.1 Hz), 145.10 – 145.26 (m), 147.49, 149.46, 154.78, 169.47; HRMS (ESI) *m/z* $[M+H]^+$ calcd for $C_{24}H_{22}FN_5O$ 416.1881, found 416.1871.

*1-(5-(4-amino-7-methyl-7H-pyrrolo[2,3-d]pyrimidin-5-yl)-4-fluoroindolin-1-yl)-2-(4-(trifluoromethyl)phenyl)ethan-1-one (**34**)* General procedure C with 5-(2,3-dihydro-1*H*-indol-5-yl)-7-methyl-7*H*-pyrrolo[2,3-*d*]pyrimidin-4-amine dihydrochloride **17** (0.1 g, 0.28 mmol) and 22-(4-(trifluoromethyl)phenyl)acetic acid (0.06 g, 0.281 mmol) to obtain 1-(5-(4-amino-7-methyl-7*H*-pyrrolo[2,3-*d*]pyrimidin-5-yl)-4-fluoroindolin-1-yl)-2-(4-(trifluoromethyl)phenyl)ethan-1-one (0.077 g, 0.164 mmol, 58 % yield). ^{1}H NMR (400 MHz, DMSO-d_6) δ 3.25 (t, *J* = 8.4 Hz, 2H), 3.73 (s, 3H), 4.02 (s, 2H), 4.31 (t, *J* = 8.4 Hz, 2H), 6.04 (br. s, 2H), 7.19 (t, *J* = 8.0 Hz, 1H), 7.26 (s, 1H), 7.52 (d, *J* = 7.9 Hz, 2H), 7.71 (d, *J* = 7.9 Hz, 2H), 7.91 (d, *J* = 8.2 Hz, 1H), 8.14 (s, 1H); ^{13}C NMR (101 MHz, DMSO-d_6) δ 24.47, 31.25, 42.05, 48.91, 101.18, 107.50, 112.60 (d, *J* = 2.4 Hz), 117.47 (d, *J* = 14.9 Hz), 119.31 (d, *J* = 23.3 Hz), 125.49 (q, *J* = 3.5 Hz), 125.77, 126.24, 127.76 (d, *J* = 31.6 Hz), 131.12, 131.36 (d, *J* = 1.8 Hz), 140.45, 144.98 (d, *J* = 8.5 Hz), 150.55, 151.91, 156.02 (d, *J* = 242.5 Hz), 157.47, 169.17; HRMS (ESI) *m/z* $[M+H]^+$ calcd for $C_{24}H_{19}F_4N_5O$ 470.1598, found 470.1589.

2 Western blot figure of non selective compounds 21, 30-33

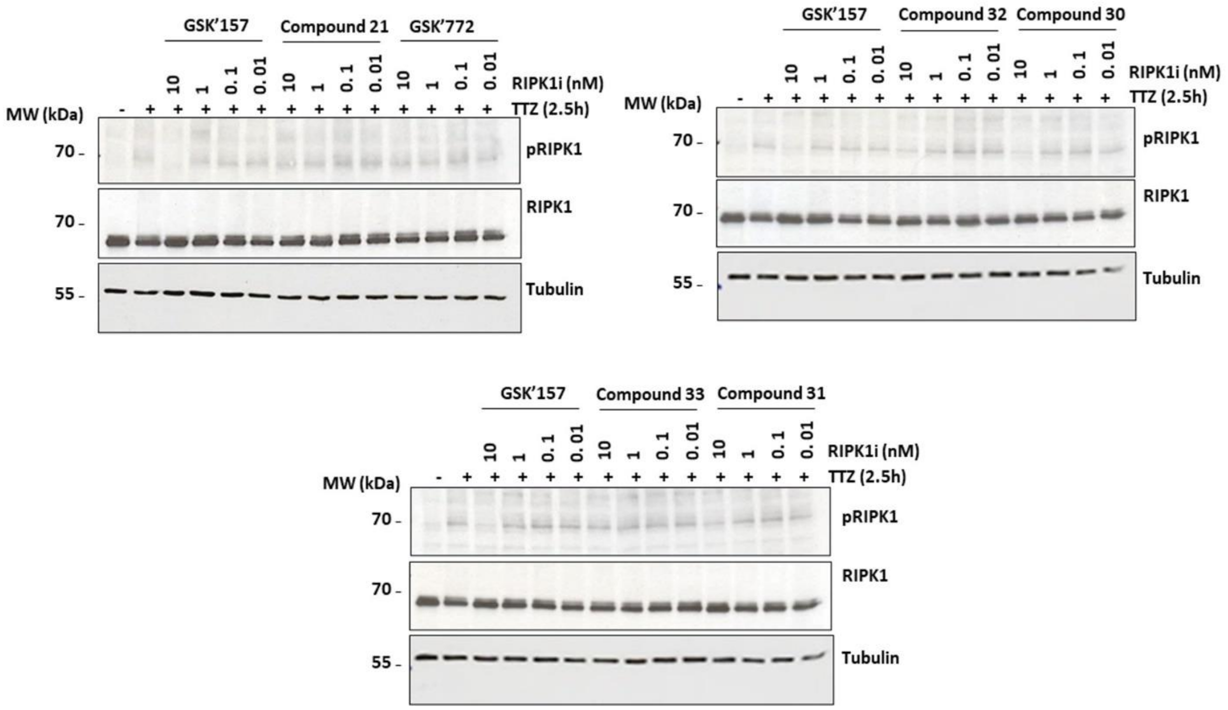

Figure S1. Western blot to compare GSK'157 with compounds 21 and 30– 33 active on PERK for the prevention of RIPK1 phosporilation. Immortalized MEFs were pretreated for 30 min with ZVAD-fmk (50 µM), TAK1-inh (1 µM) and the indicated compounds and then stimulated for 2.5 h with hTNF (20 ng/ml).

3 HRMS spectra

Compound 18

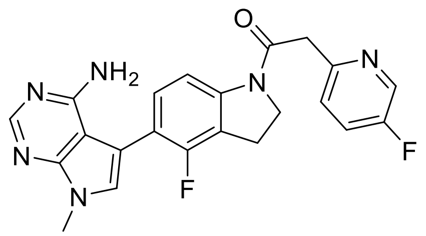

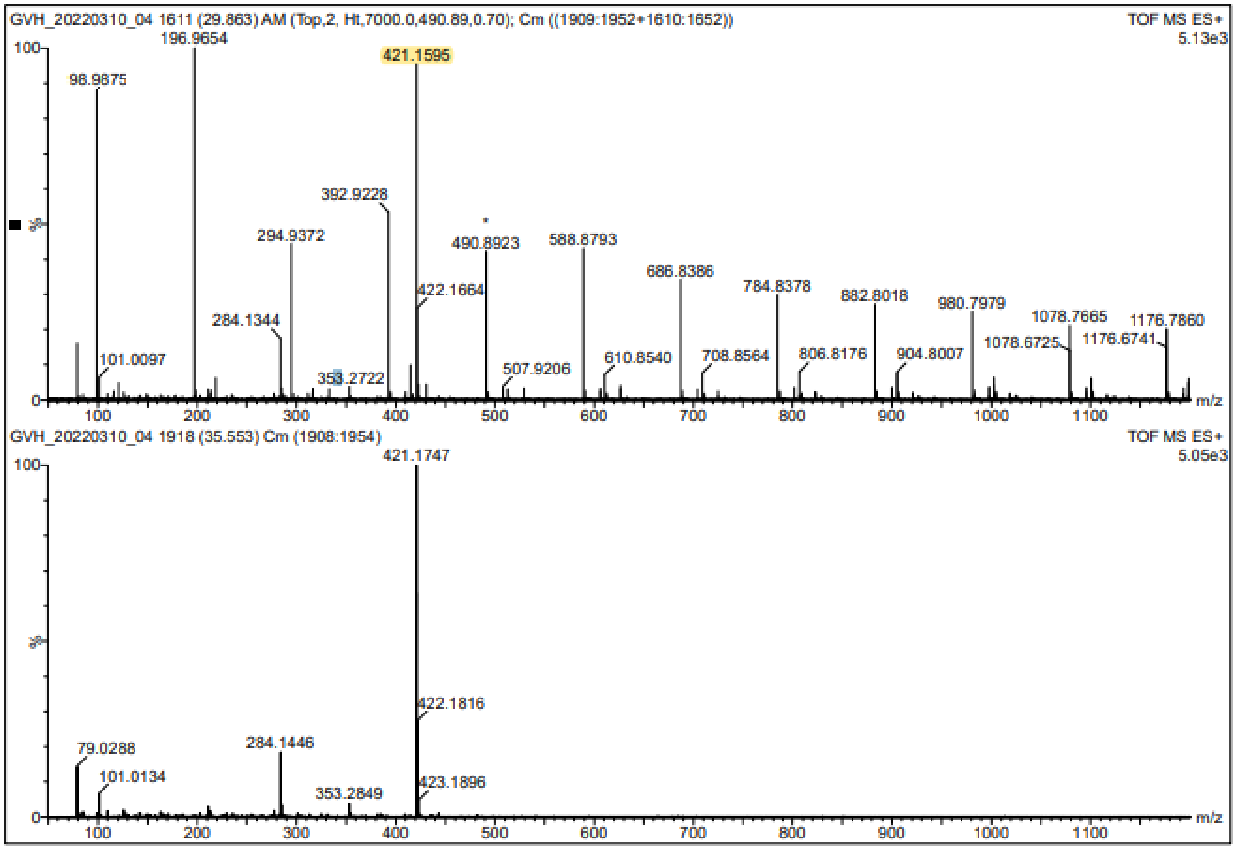

Compound 19

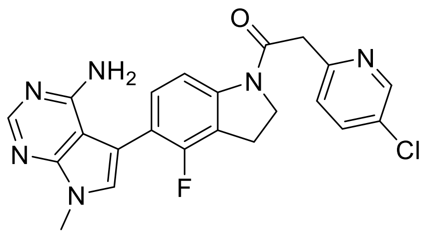

NH2
N
N
N
O
N
N
F
Cl

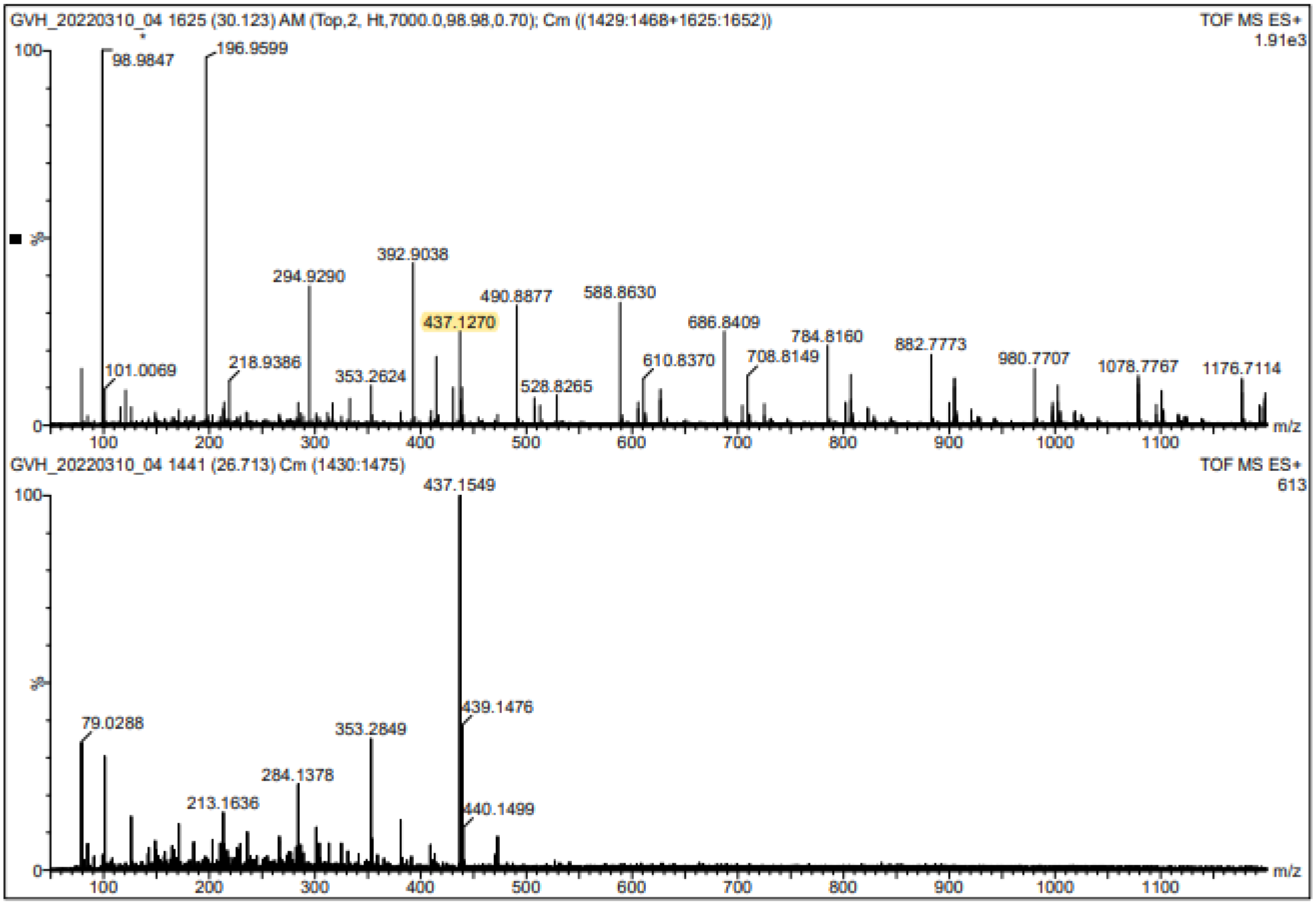

GVH_20220310_04 1625 (30.123) AM (Top,2, Ht,7000.0,98.98,0.70); Cm ((1429:1468+1625:1652))
TOF MS ES+
1.91e3
98.9847
196.9599
392.9038
294.9290
490.8877
588.8630
437.1270
686.8409
784.8160
882.7773
610.8370
708.8149
980.7707
1078.7767
1176.7114
101.0069
218.9386
353.2624
528.8265
m/z
GVH_20220310_04 1441 (26.713) Cm (1430:1475)
TOF MS ES+
613
437.1549
439.1476
79.0288
353.2849
284.1378
213.1636
440.1499

Compound 20

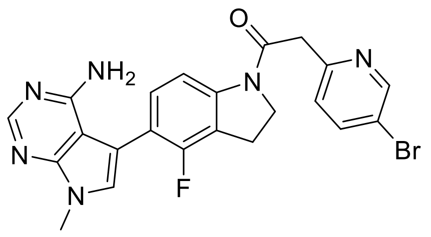
O
NH2
N
N
N
N
N
F
Br

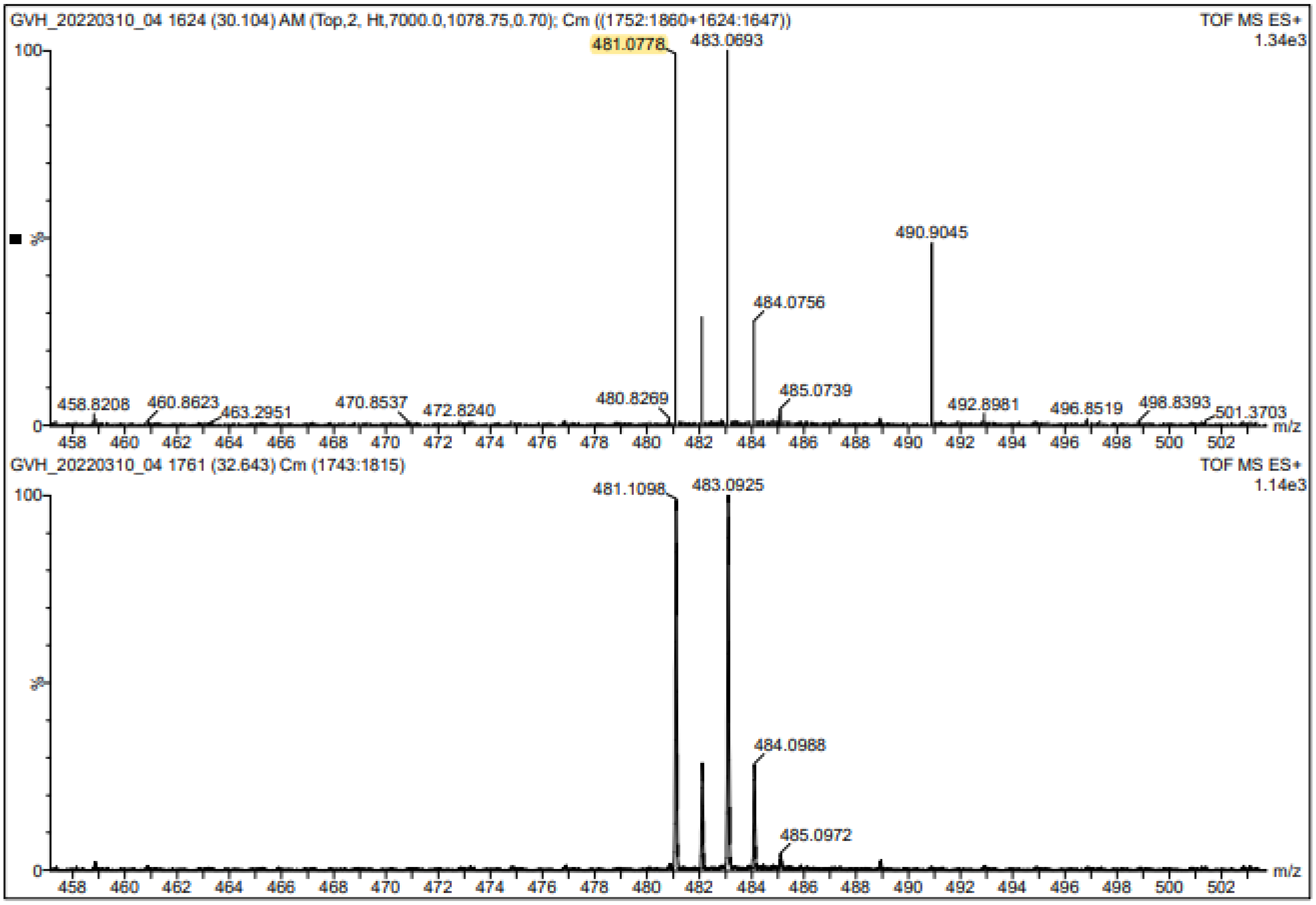
GVH_20220310_04 1624 (30.104) AM (Top,2, Ht,7000.0,1078.75,0.70); Cm ((1752:1860+1624:1647))
TOF MS ES+
1.34e3
481.0778
483.0693
490.9045
484.0756
485.0739
458.8208
460.8623
463.2951
470.8537
472.8240
480.8269
492.8981
496.8519
498.8393
501.3703
m/z
GVH_20220310_04 1761 (32.643) Cm (1743:1815)
TOF MS ES+
1.14e3
481.1098
483.0925
484.0988
485.0972
m/z

Compound 21

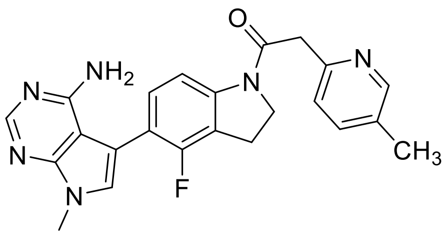
O
N
NH2
N
N
N
F
N
CH3

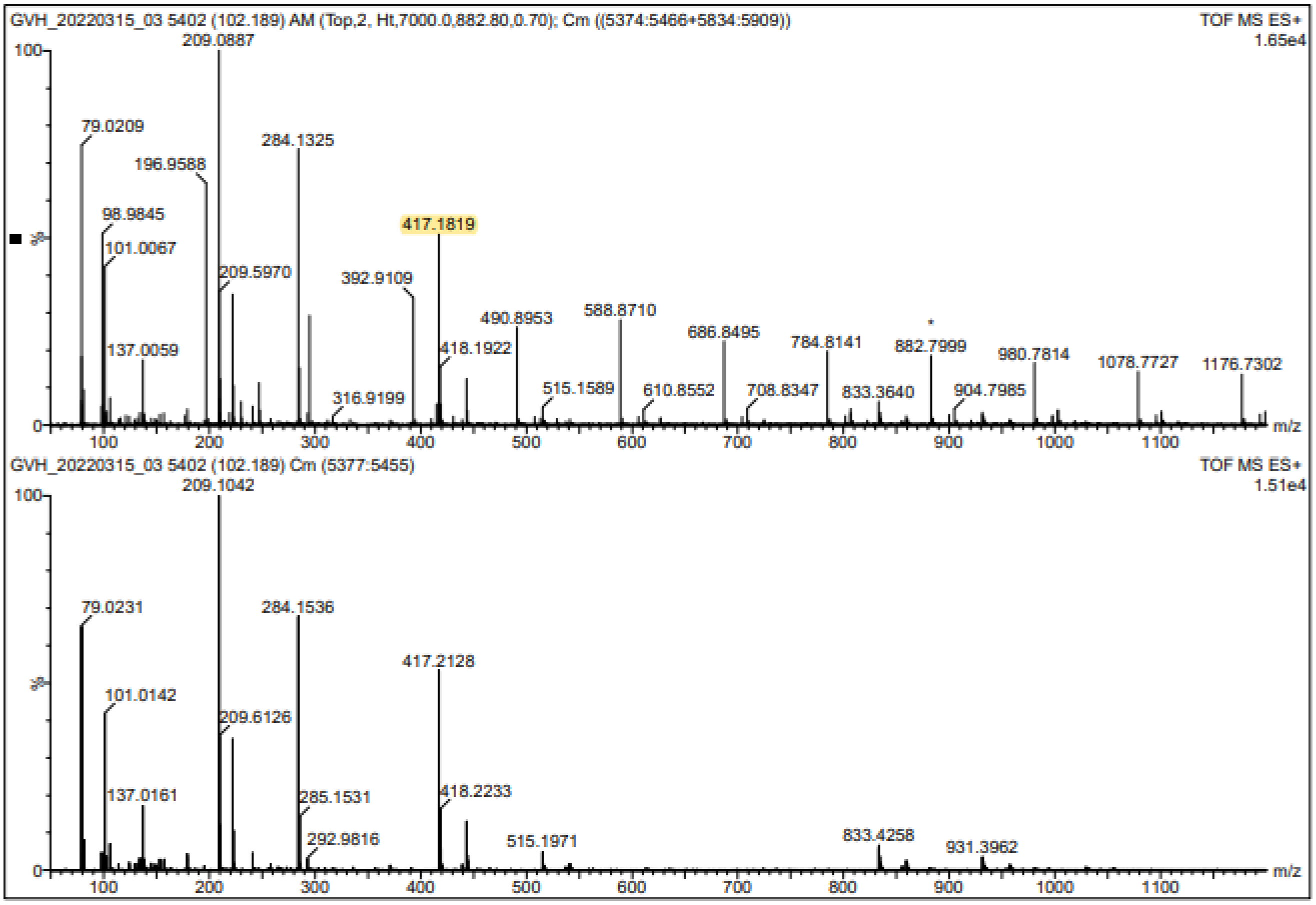
GVH_20220315_03 5402 (102.189) AM (Top,2, Ht,7000.0,882.80,0.70); Cm ((5374:5466+5834:5909))
TOF MS ES+
1.65e4
100
%
0
209.0887
79.0209
284.1325
196.9588
98.9845
101.0067
417.1819
209.5970
392.9109
490.8953
588.8710
686.8495
784.8141
882.7999
980.7814
1078.7727
1176.7302
137.0059
418.1922
515.1589
610.8552
708.8347
833.3640
904.7985
316.9199
m/z
100
200
300
400
500
600
700
800
900
1000
1100
GVH_20220315_03 5402 (102.189) Cm (5377:5455)
TOF MS ES+
1.51e4
209.1042
79.0231
284.1536
417.2128
101.0142
209.6126
137.0161
285.1531
418.2233
292.9816
515.1971
833.4258
931.3962

Compound 22 (UAMC-3861)

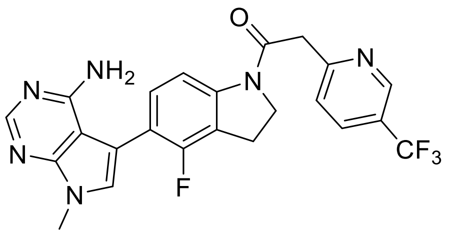

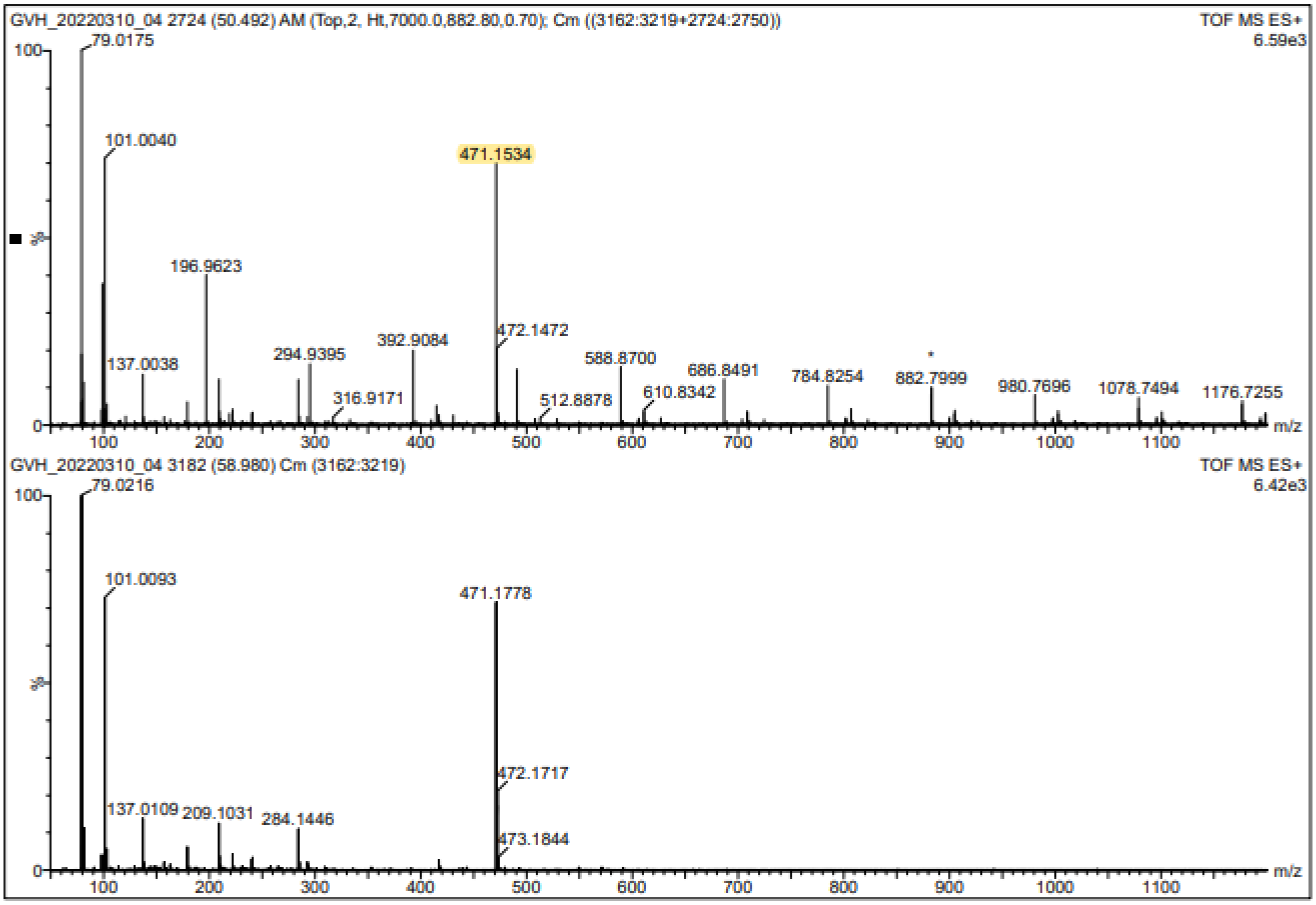

Compound 23

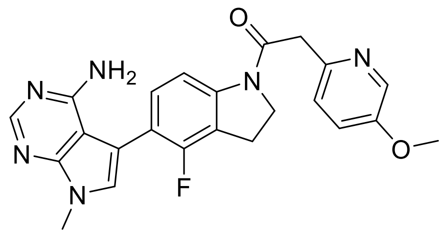

NH2
N
N
N
F
N
O
N
O

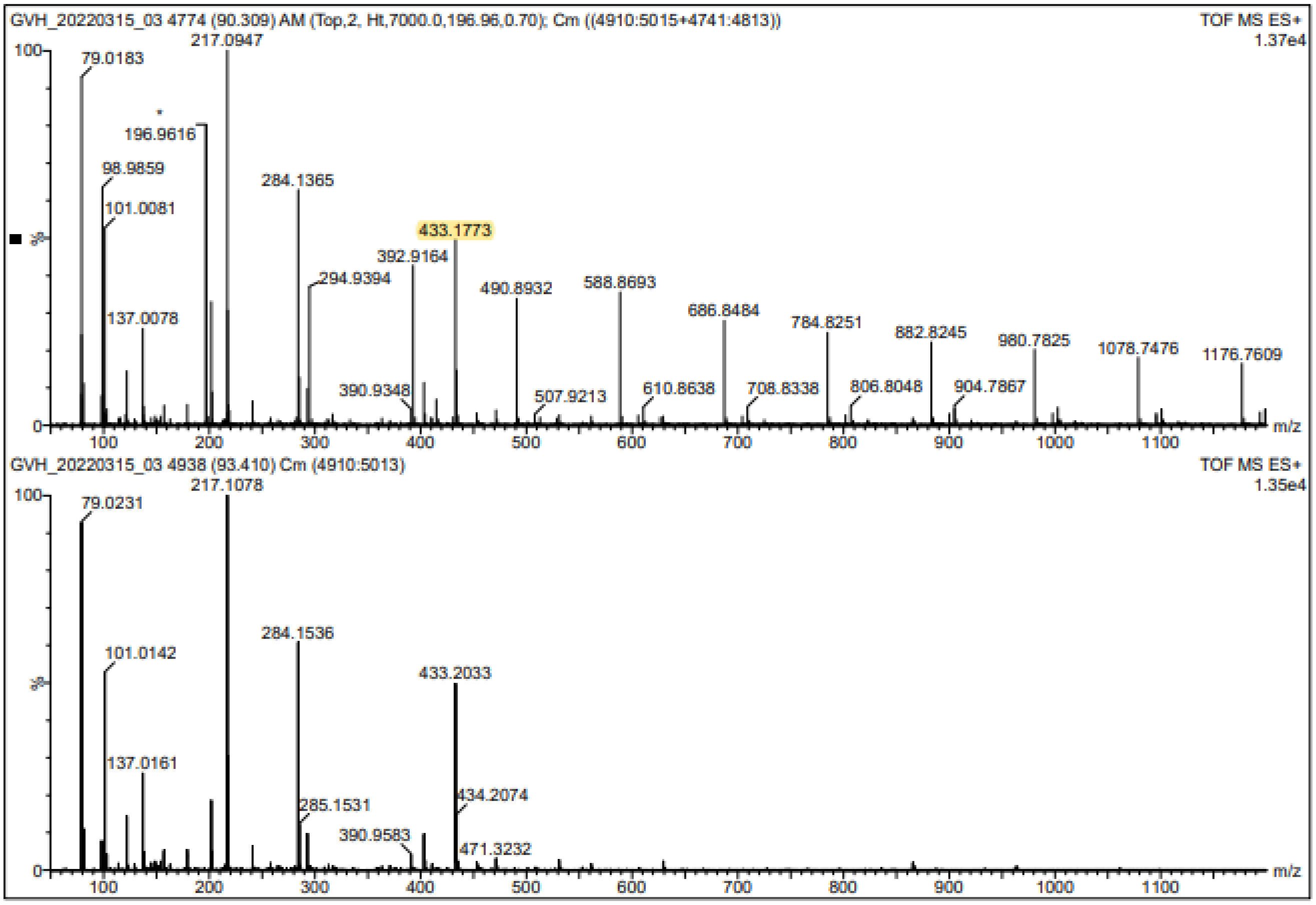

GVH_20220315_03 4774 (90.309) AM (Top,2, Ht,7000.0,196.96,0.70); Cm ((4910:5015+4741:4813))
TOF MS ES+
1.37e4
217.0947
79.0183
196.9616
98.9859
284.1365
101.0081
433.1773
392.9164
294.9394
490.8932
588.8693
137.0078
686.8484
784.8251
882.8245
980.7825
1078.7476
1176.7609
390.9348
507.9213
610.8638
708.8338
806.8048
904.7867
m/z
GVH_20220315_03 4938 (93.410) Cm (4910:5013)
TOF MS ES+
1.35e4
217.1078
79.0231
284.1536
101.0142
433.2033
137.0161
434.2074
285.1531
390.9583
471.3232

Compound 24

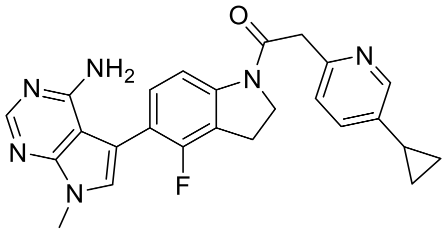
O
NH2
N
N
N
N
N
F

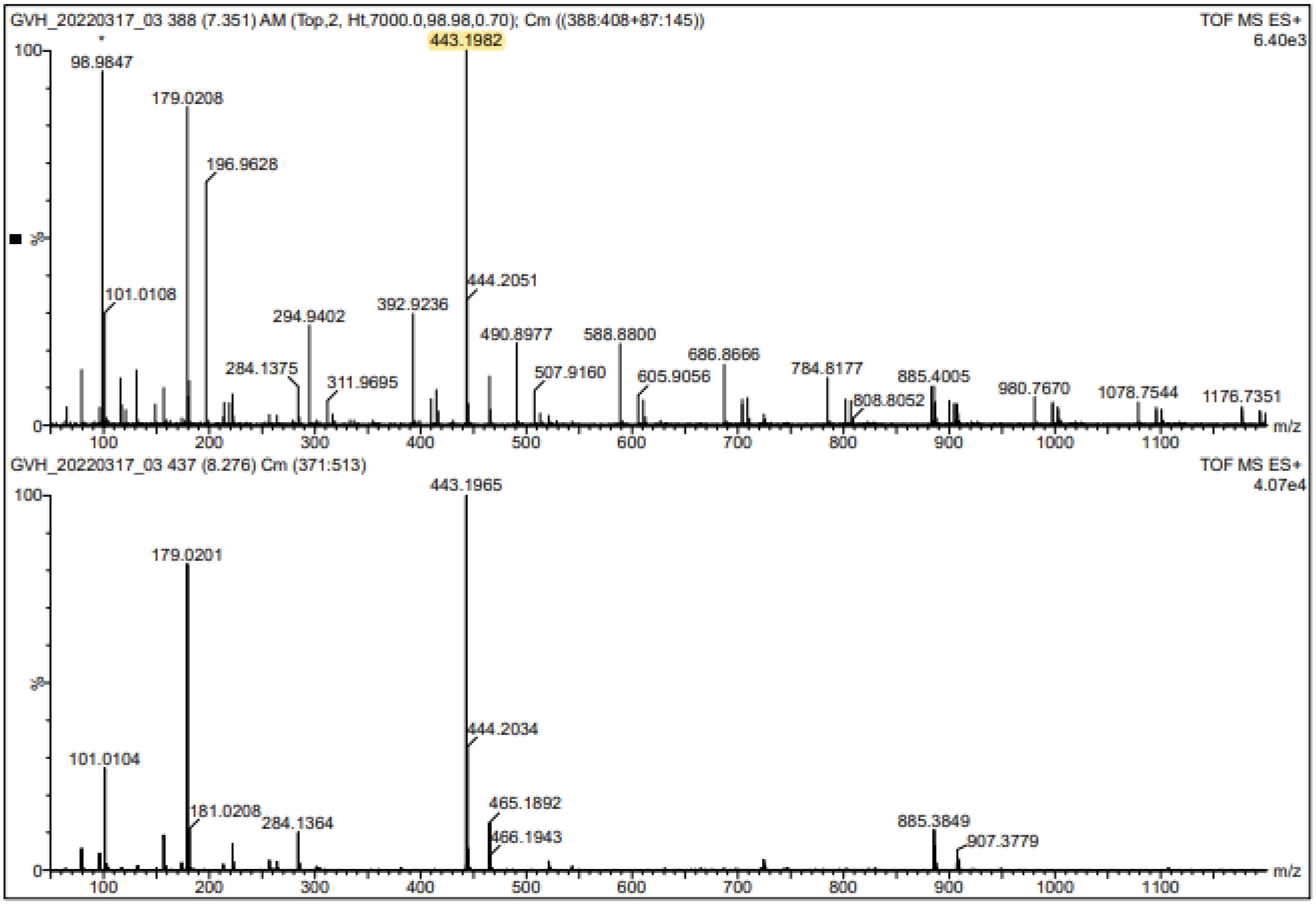
GVH_20220317_03 388 (7.351) AM (Top,2, Ht,7000.0,98.98,0.70); Cm ((388:408+87:145))
TOF MS ES+
6.40e3
443.1982
98.9847
179.0208
196.9628
101.0108
294.9402
392.9236
444.2051
490.8977
588.8800
284.1375
311.9695
507.9160
605.9056
686.8666
784.8177
808.8052
885.4005
980.7670
1078.7544
1176.7351
%
m/z
GVH_20220317_03 437 (8.276) Cm (371:513)
TOF MS ES+
4.07e4
443.1965
179.0201
444.2034
101.0104
465.1892
181.0208
284.1364
466.1943
885.3849
907.3779
%
m/z

Compound 25

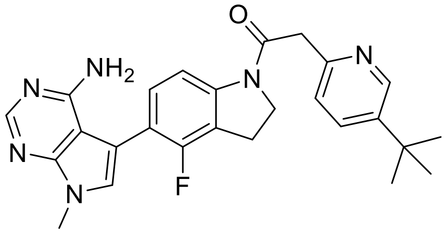

O
N
NH2
N
N
N
N
F

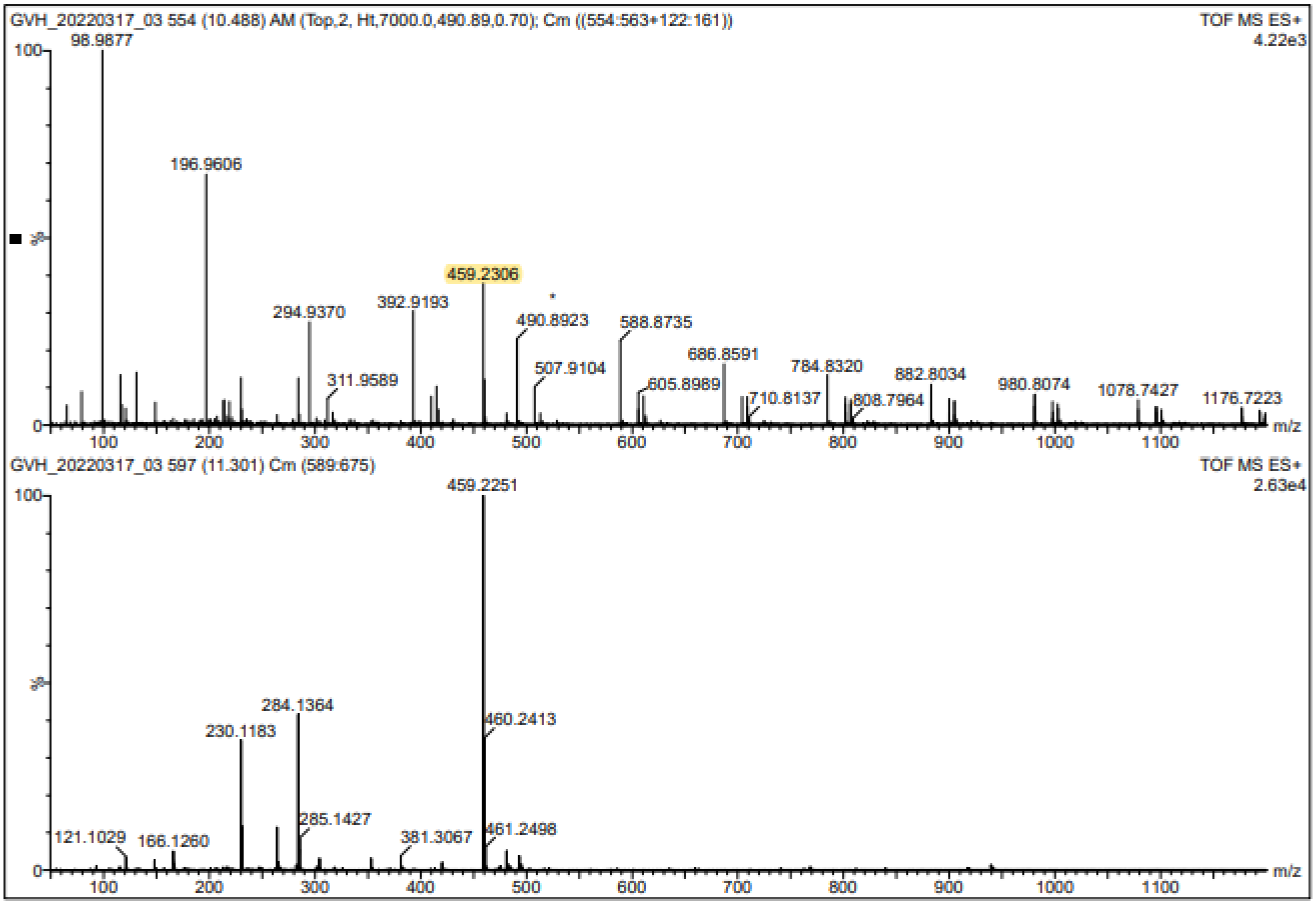

GVH_20220317_03 554 (10.488) AM (Top,2, Ht,7000.0,490.89,0.70); Cm ((554:563+122:161))
TOF MS ES+
4.22e3
98.9877
196.9606
459.2306
294.9370
392.9193
490.8923
588.8735
507.9104
686.8591
311.9589
605.8989
784.8320
882.8034
980.8074
1078.7427
1176.7223
710.8137
808.7964
%
m/z
GVH_20220317_03 597 (11.301) Cm (589:675)
TOF MS ES+
2.63e4
459.2251
284.1364
460.2413
230.1183
285.1427
461.2498
121.1029
166.1260
381.3067
%
m/z

Compound 26

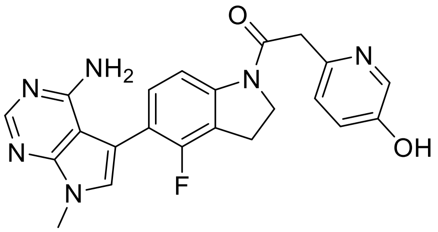

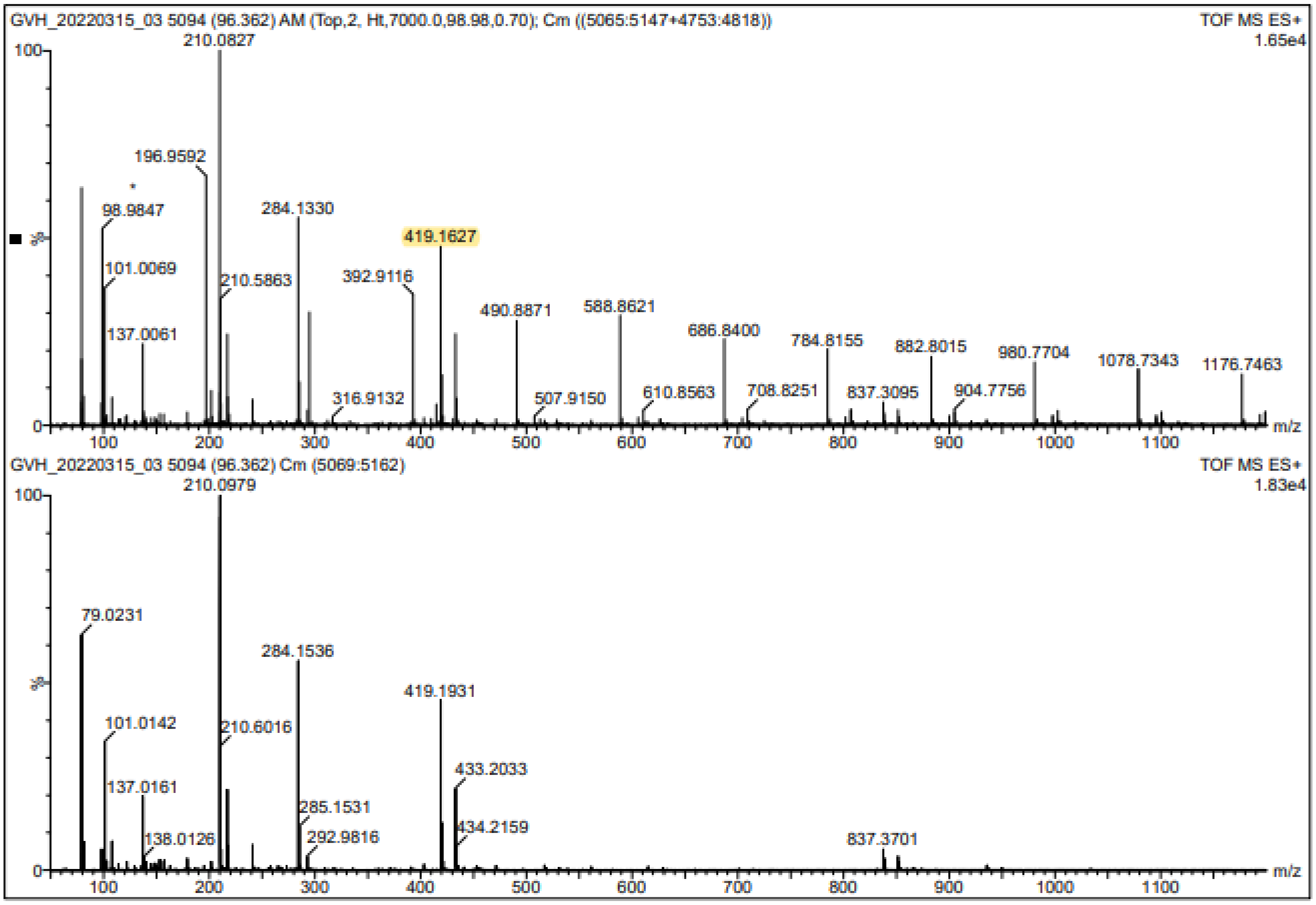
GVH_20220315_03 5094 (96.362) AM (Top,2, Ht,7000.0,98.98,0.70); Cm ((5065:5147+4753:4818))
TOF MS ES+
1.65e4
210.0827
196.9592
98.9847
284.1330
419.1627
101.0069
210.5863
392.9116
490.8871
588.8621
137.0061
686.8400
784.8155
882.8015
980.7704
1078.7343
1176.7463
316.9132
507.9150
610.8563
708.8251
837.3095
904.7756
m/z
GVH_20220315_03 5094 (96.362) Cm (5069:5162)
TOF MS ES+
1.83e4
210.0979
79.0231
284.1536
419.1931
101.0142
210.6016
433.2033
137.0161
285.1531
434.2159
138.0126
292.9816
837.3701
m/z

Compound 27

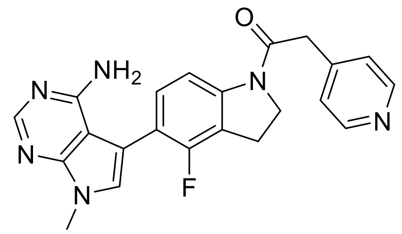

O
NH2
N
N
N
N
N
F

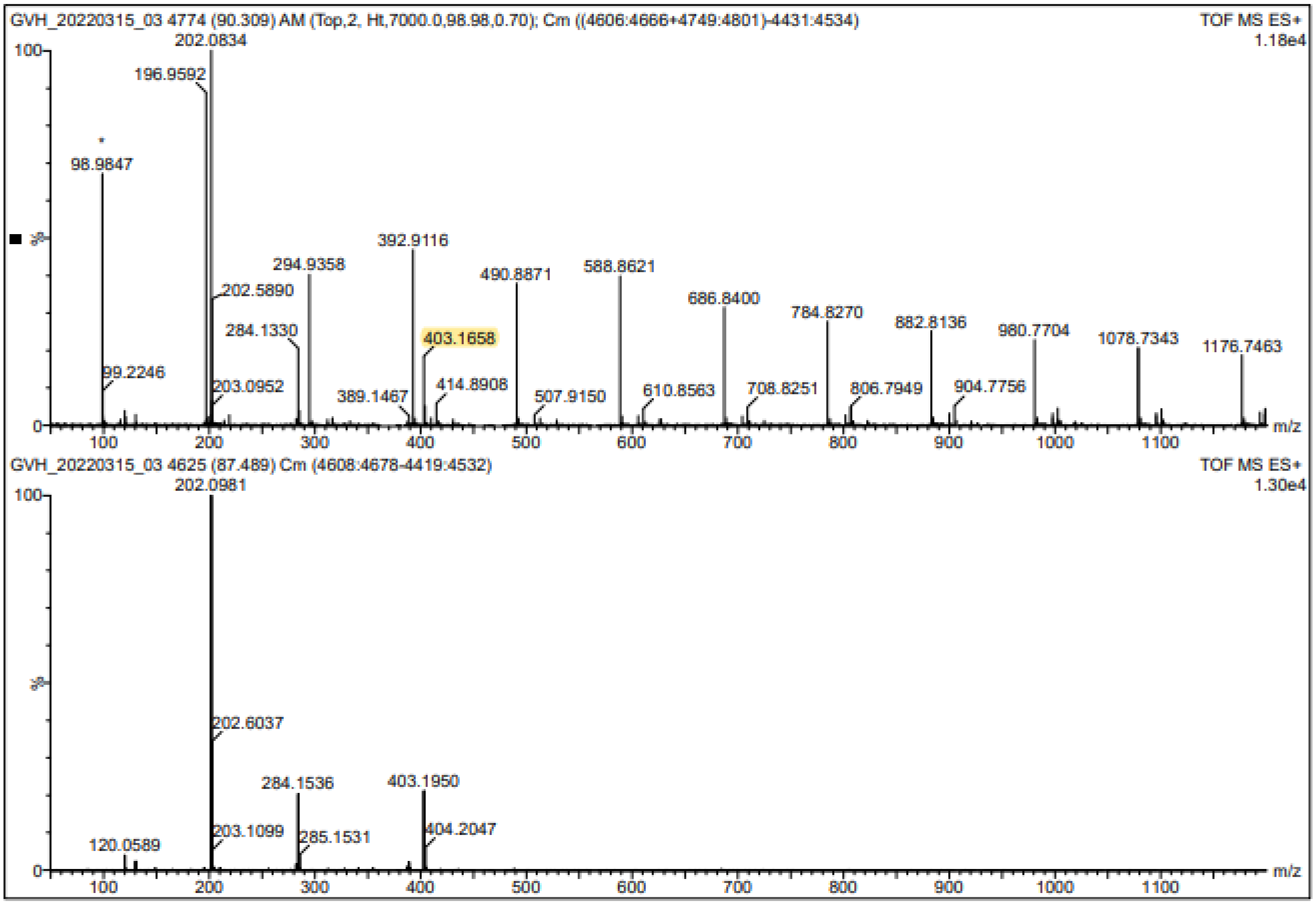

GVH_20220315_03 4774 (90.309) AM (Top,2, Ht,7000.0,98.98,0.70); Cm ((4606:4666+4749:4801)-4431:4534)
TOF MS ES+
1.18e4
202.0834
196.9592
98.9847
99.2246
202.5890
203.0952
284.1330
294.9358
392.9116
389.1467
403.1658
414.8908
490.8871
507.9150
588.8621
610.8563
686.8400
708.8251
784.8270
806.7949
882.8136
904.7756
980.7704
1078.7343
1176.7463
m/z
GVH_20220315_03 4625 (87.489) Cm (4608:4678-4419:4532)
TOF MS ES+
1.30e4
202.0981
202.6037
120.0589
203.1099
284.1536
285.1531
403.1950
404.2047
%
100
0
m/z

Compound 28

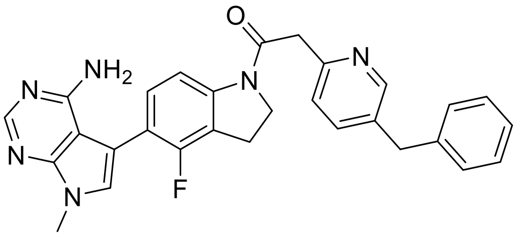

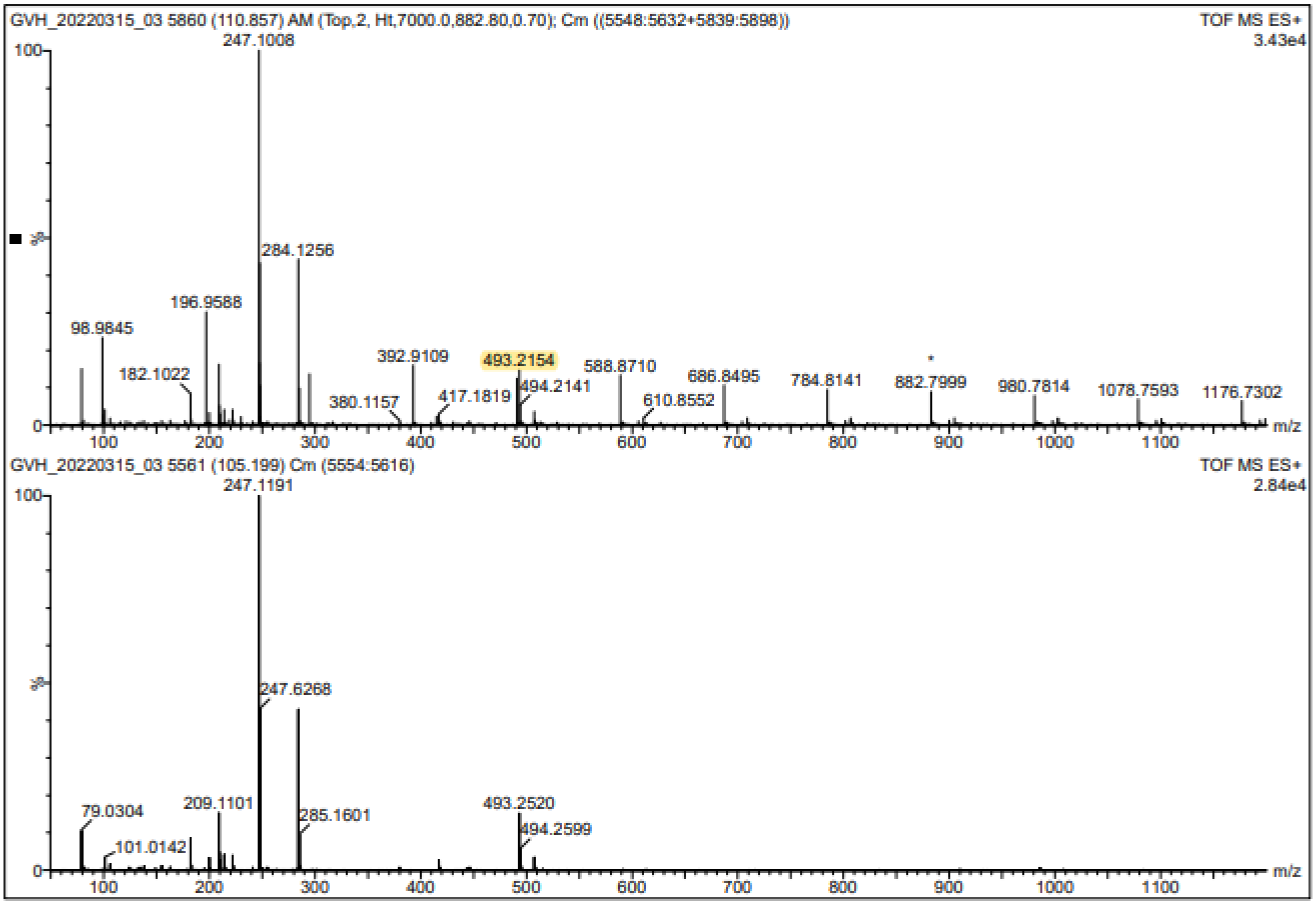
GVH_20220315_03 5860 (110.857) AM (Top,2, Ht,7000.0,882.80,0.70); Cm ((5548:5632+5839:5898))
TOF MS ES+
3.43e4
247.1008
284.1256
196.9588
98.9845
182.1022
392.9109
493.2154
588.8710
686.8495
784.8141
882.7999
980.7814
1078.7593
1176.7302
494.2141
417.1819
380.1157
610.8552
m/z
GVH_20220315_03 5561 (105.199) Cm (5554:5616)
TOF MS ES+
2.84e4
247.1191
247.6268
79.0304
209.1101
285.1601
493.2520
494.2599
101.0142

Compound 29

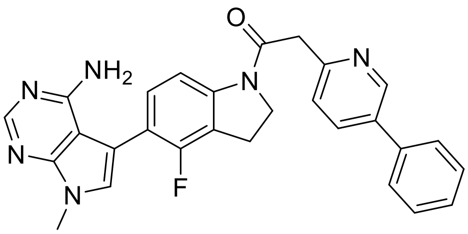

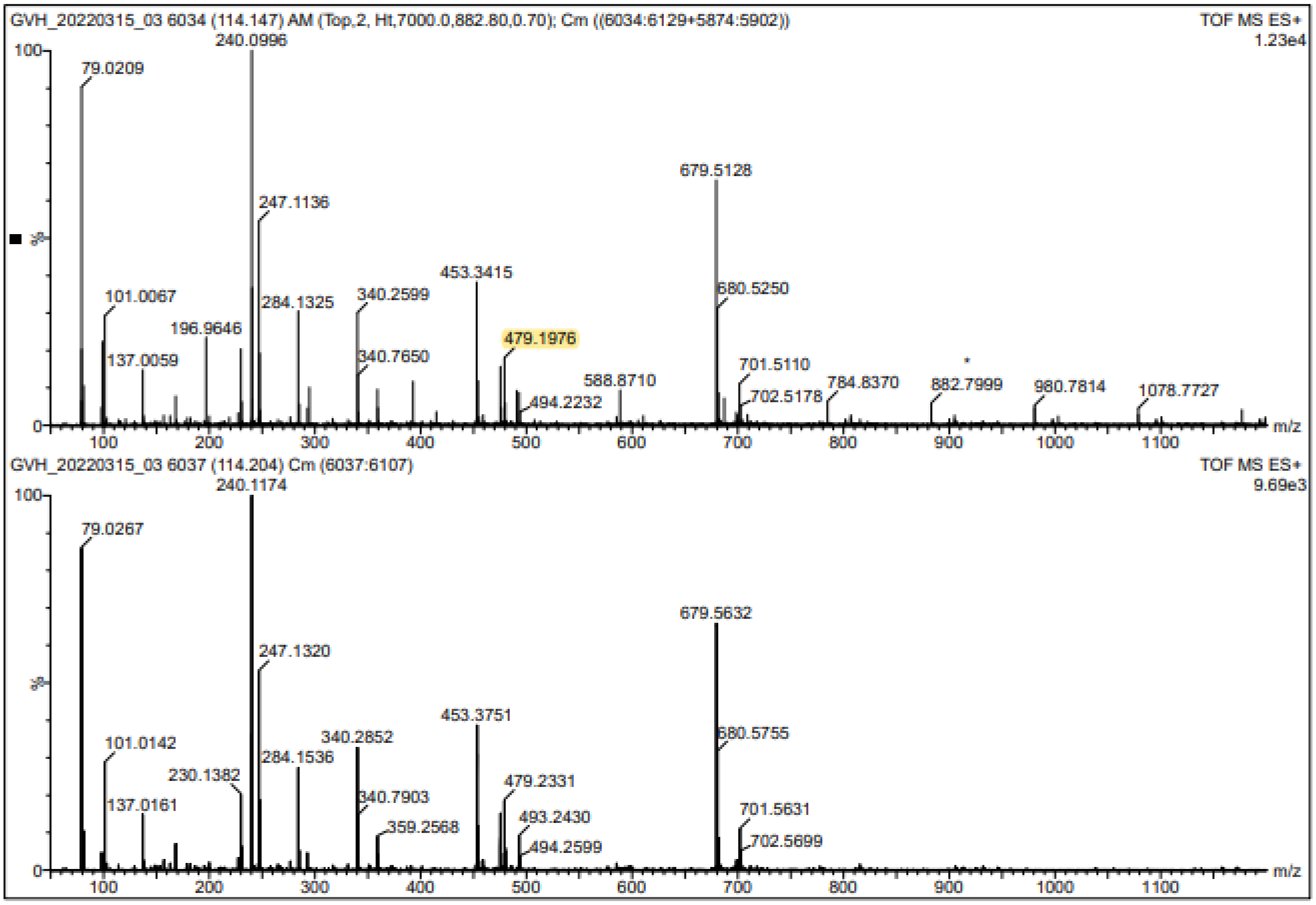

GVH_20220315_03 6034 (114.147) AM (Top,2, Ht,7000.0,882.80,0.70); Cm ((6034:6129+5874:5902))
TOF MS ES+
1.23e4
240.0996
79.0209
679.5128
247.1136
453.3415
680.5250
101.0067
284.1325
340.2599
196.9646
479.1976
137.0059
340.7650
701.5110
588.8710
784.8370
882.7999
980.7814
1078.7727
702.5178
494.2232
GVH_20220315_03 6037 (114.204) Cm (6037:6107)
TOF MS ES+
9.69e3
240.1174
79.0267
679.5632
247.1320
453.3751
680.5755
101.0142
340.2852
284.1536
230.1382
479.2331
137.0161
340.7903
701.5631
493.2430
359.2568
702.5699
494.2599
m/z

Compound 30

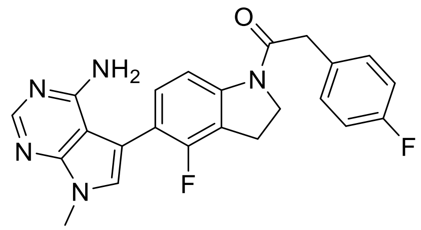
NH2
N
N
N
F
N
O
F

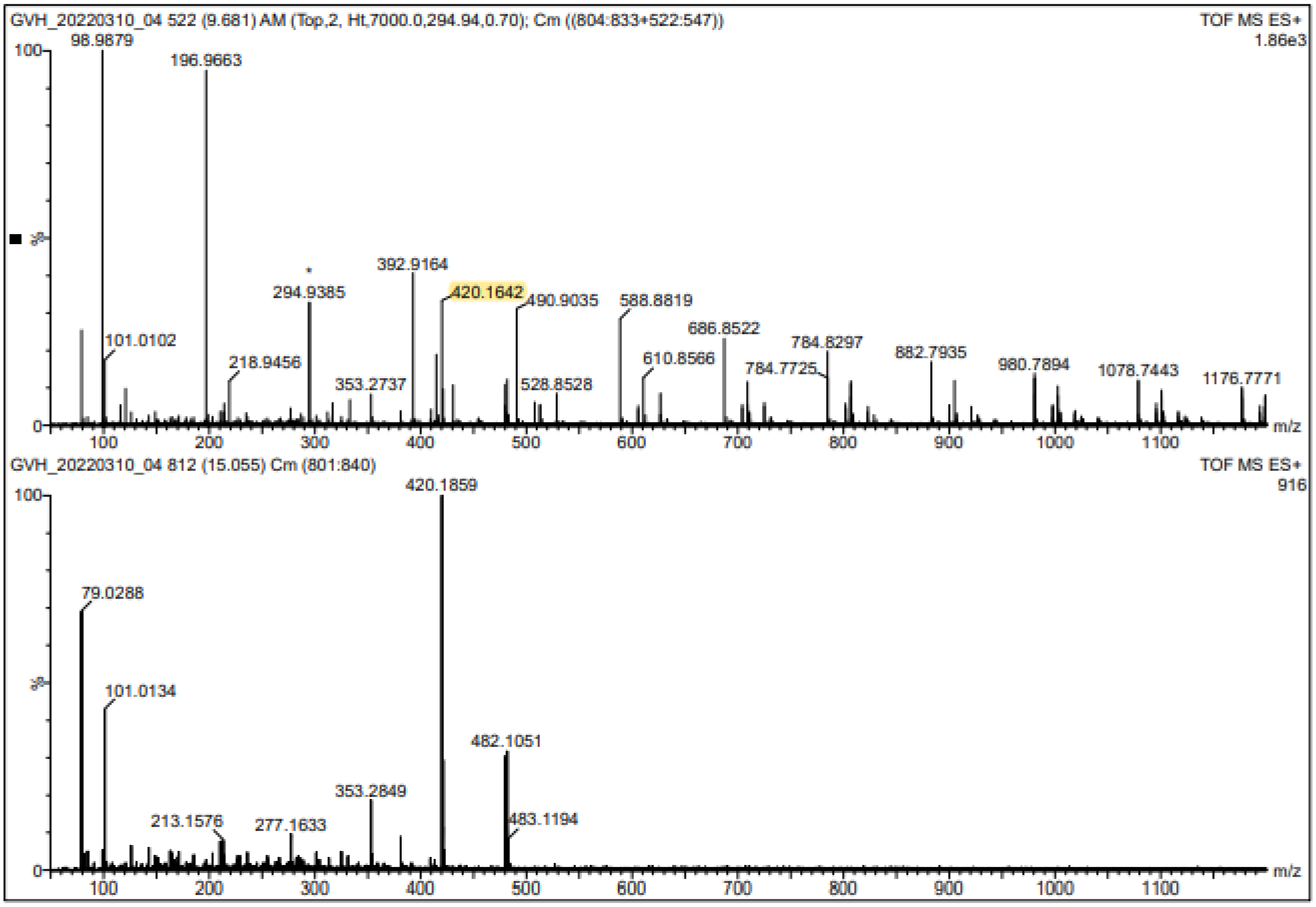
GVH_20220310_04 522 (9.681) AM (Top,2, Ht,7000.0,294.94,0.70); Cm ((804:833+522:547))
TOF MS ES+
1.86e3
98.9879
196.9663
392.9164
294.9385
420.1642
490.9035
588.8819
686.8522
784.8297
882.7935
980.7894
1078.7443
1176.7771
101.0102
218.9456
353.2737
528.8528
610.8566
784.7725
%
m/z
GVH_20220310_04 812 (15.055) Cm (801:840)
TOF MS ES+
916
420.1859
79.0288
101.0134
482.1051
353.2849
213.1576
277.1633
483.1194

Compound 31

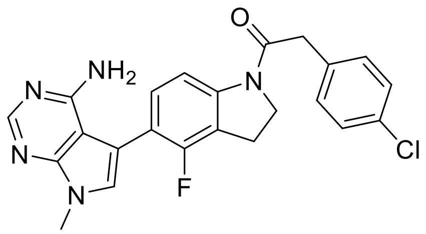

NH2
N
N
N
O
N
F
Cl

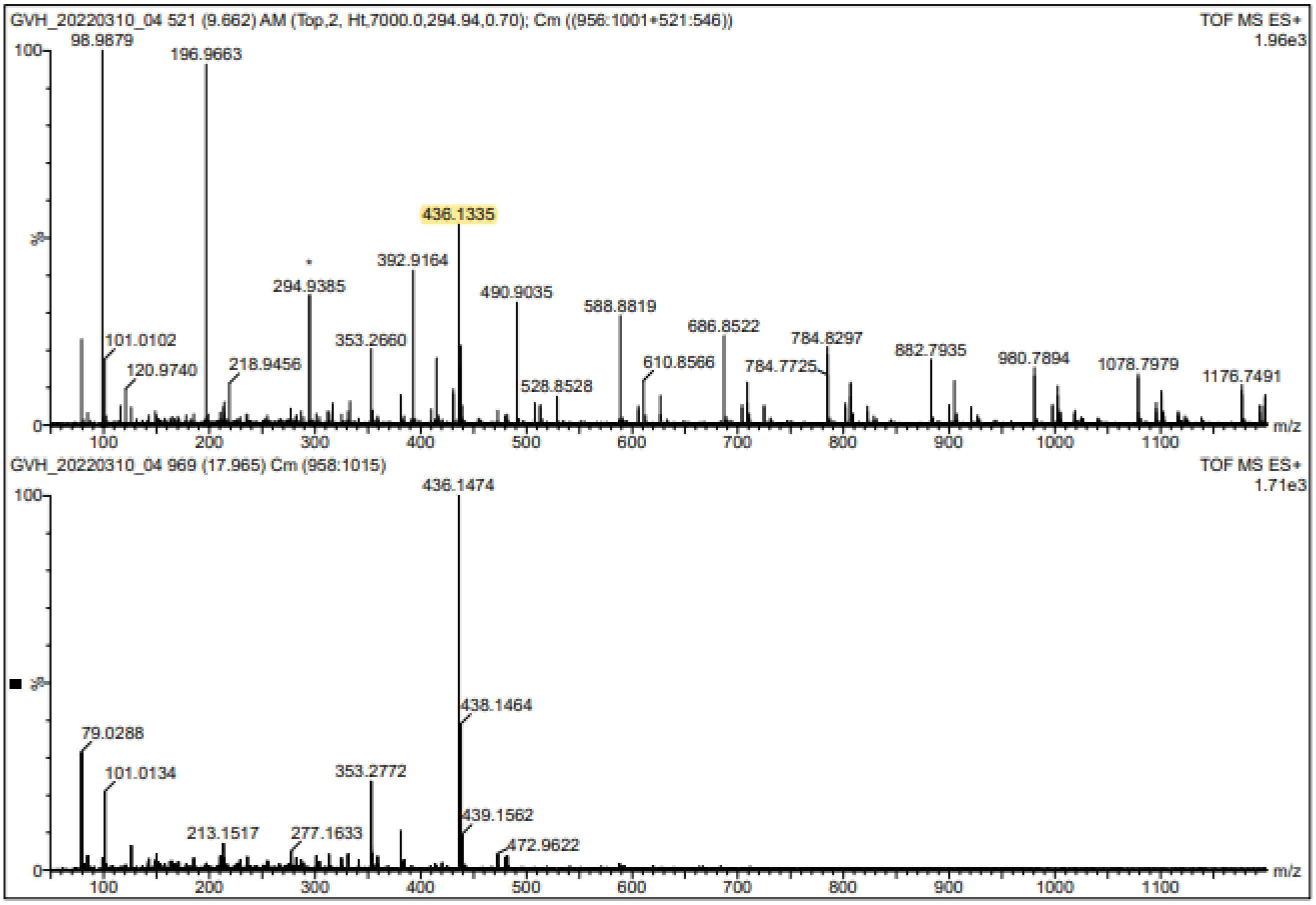

GVH_20220310_04 521 (9.662) AM (Top,2, Ht,7000.0,294.94,0.70); Cm ((956:1001+521:546))
TOF MS ES+
1.96e3
98.9879
196.9663
436.1335
392.9164
294.9385
490.9035
588.8819
686.8522
784.8297
882.7935
980.7894
1078.7979
1176.7491
101.0102
120.9740
218.9456
353.2660
610.8566
784.7725
528.8528
m/z
GVH_20220310_04 969 (17.965) Cm (958:1015)
TOF MS ES+
1.71e3
436.1474
438.1464
79.0288
101.0134
353.2772
213.1517
277.1633
439.1562
472.9622
m/z

Compound 32

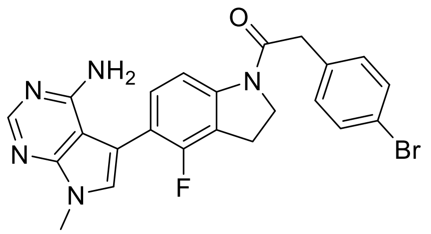
NH2
N
N
N
F
O
N
Br

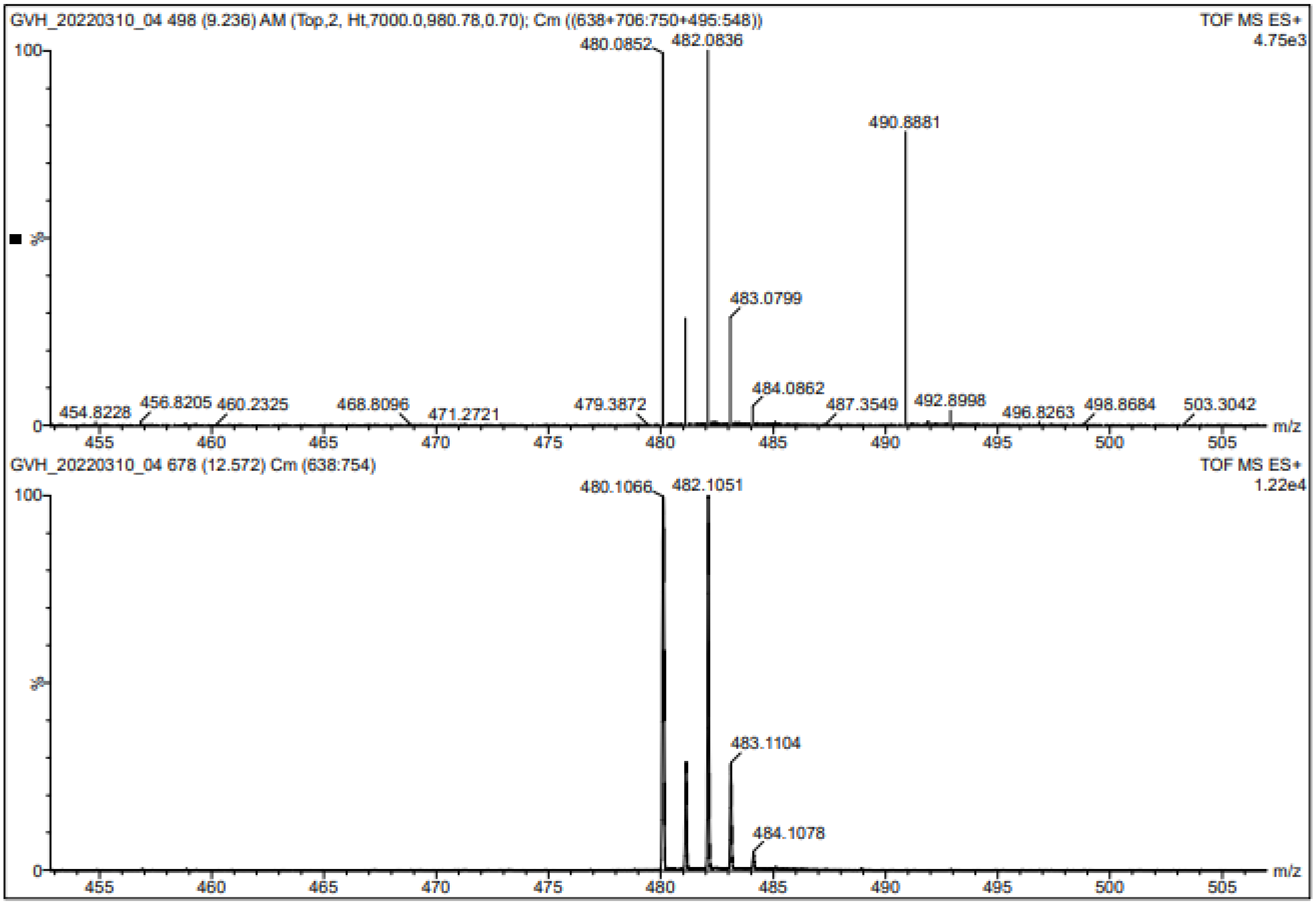
GVH_20220310_04 498 (9.236) AM (Top,2, Ht,7000.0,980.78,0.70); Cm ((638+706:750+495:548))
TOF MS ES+
4.75e3
480.0852
482.0836
490.8881
483.0799
484.0862
487.3549
492.8998
496.8263
498.8684
503.3042
454.8228
456.8205
460.2325
468.8096
471.2721
479.3872
m/z
GVH_20220310_04 678 (12.572) Cm (638:754)
TOF MS ES+
1.22e4
480.1066
482.1051
483.1104
484.1078
m/z

Compound 33

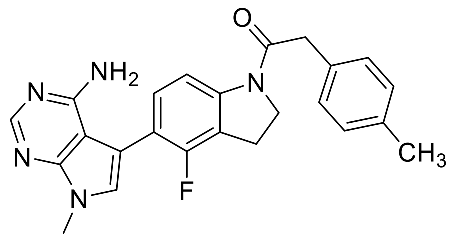

NH2
N
N
N
N
O
F
CH3

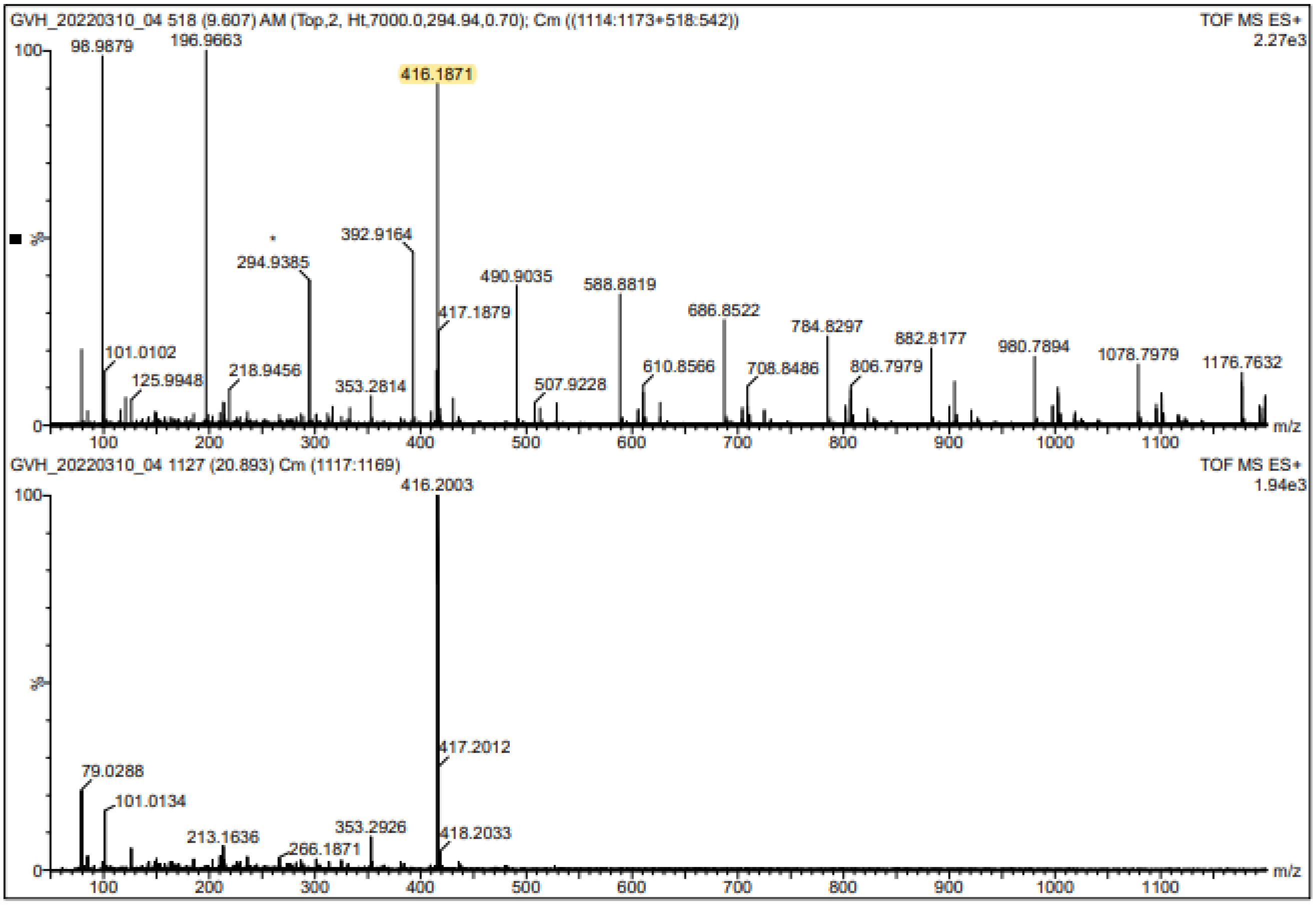

GVH_20220310_04 518 (9.607) AM (Top,2, Ht,7000.0,294.94,0.70); Cm ((1114:1173+518:542))
TOF MS ES+
2.27e3
98.9879
196.9663
416.1871
392.9164
294.9385
490.9035
588.8819
417.1879
686.8522
784.8297
882.8177
980.7894
1078.7979
1176.7632
101.0102
125.9948
218.9456
353.2814
507.9228
610.8566
708.8486
806.7979
GVH_20220310_04 1127 (20.893) Cm (1117:1169)
TOF MS ES+
1.94e3
416.2003
417.2012
79.0288
101.0134
213.1636
266.1871
353.2926
418.2033
%
m/z

Compound 34

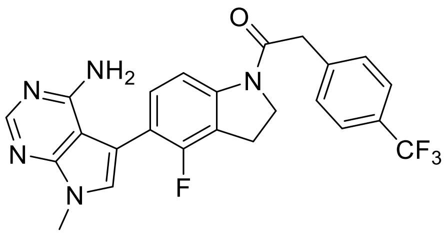

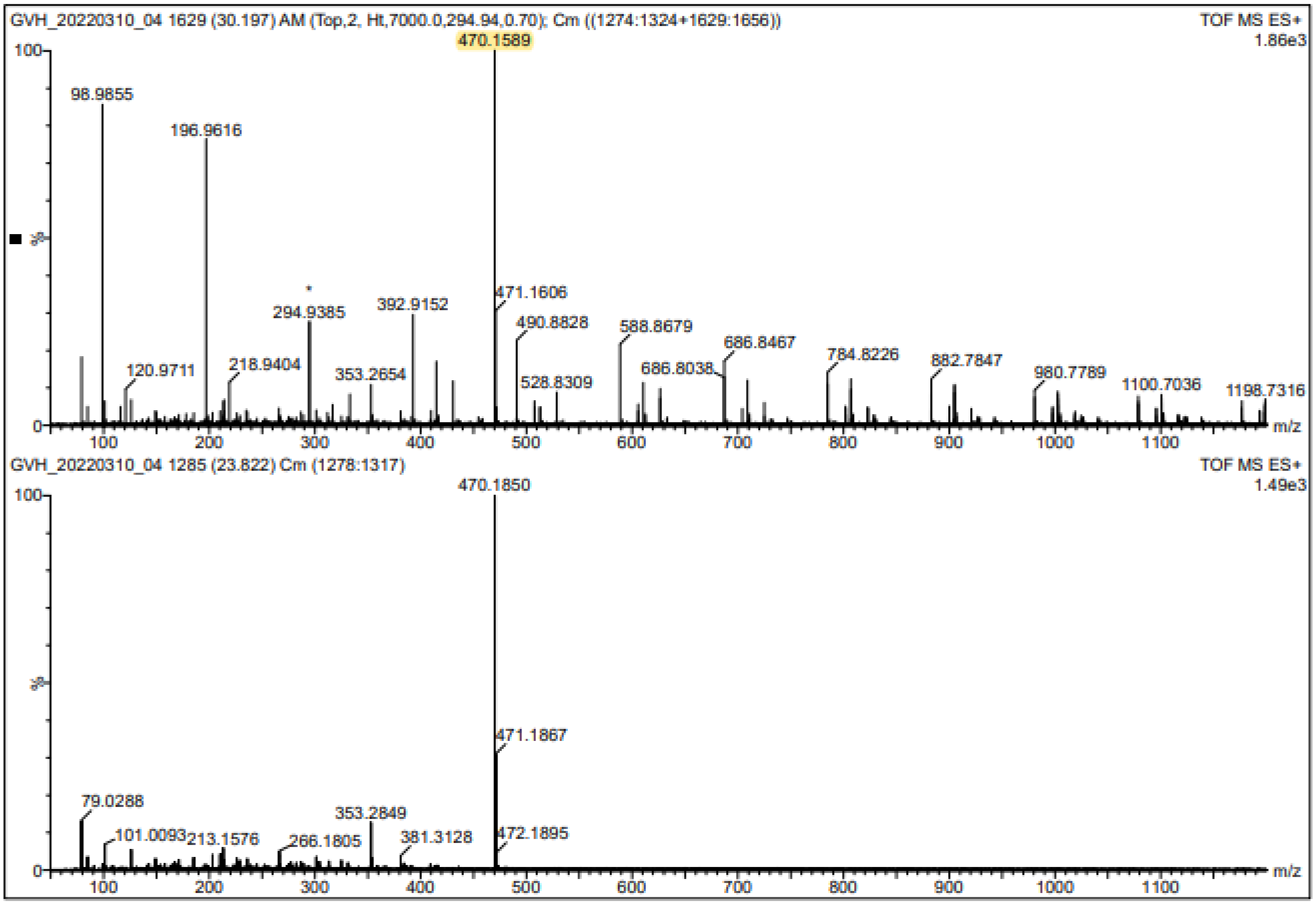
GVH_20220310_04 1629 (30.197) AM (Top,2, Ht,7000.0,294.94,0.70); Cm ((1274:1324+1629:1656))
TOF MS ES+
1.86e3
470.1589
98.9855
196.9616
294.9385
392.9152
471.1606
490.8828
588.8679
686.8467
784.8226
882.7847
980.7789
1100.7036
1198.7316
120.9711
218.9404
353.2654
528.8309
686.8038
m/z
GVH_20220310_04 1285 (23.822) Cm (1278:1317)
TOF MS ES+
1.49e3
470.1850
471.1867
79.0288
101.0093
213.1576
266.1805
353.2849
381.3128
472.1895

4 Dose-response curve for compounds 18-34

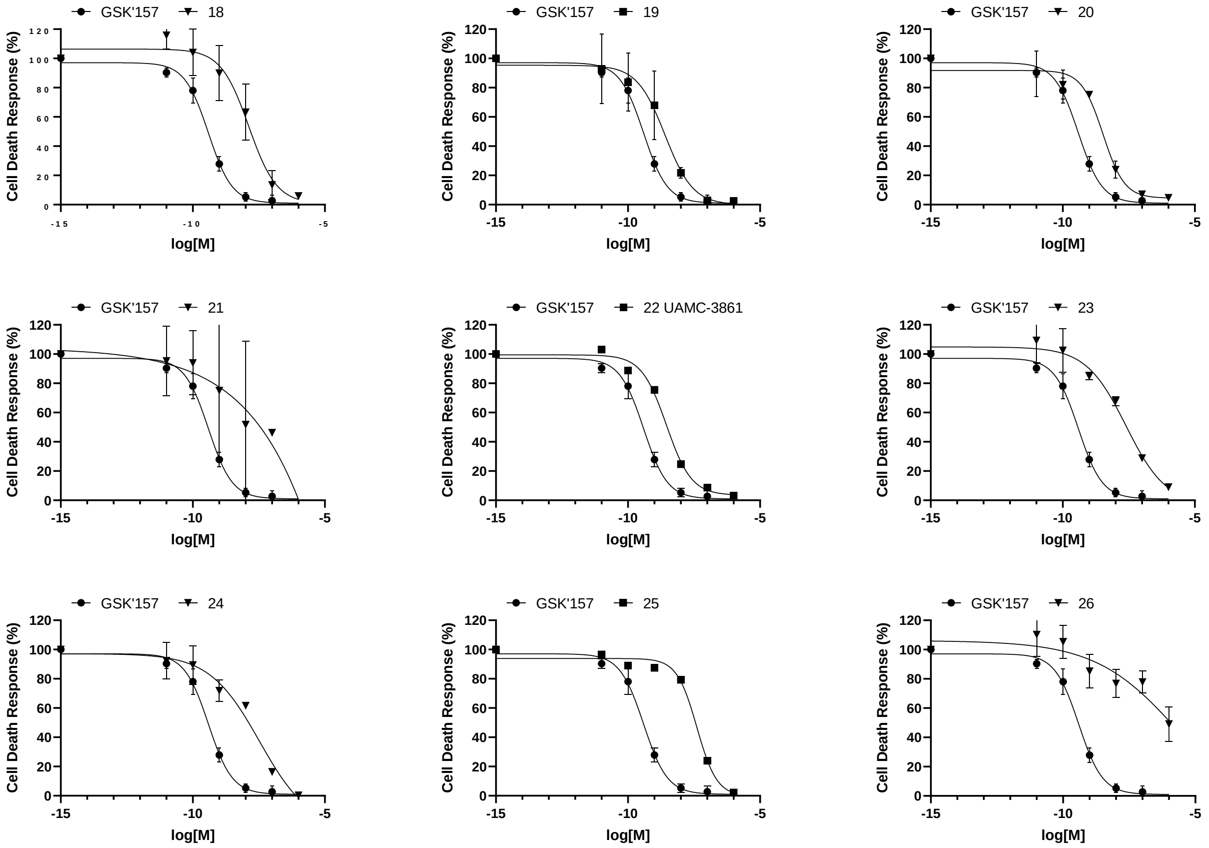

Figure S2. Represensation of the single curve for each compound in comparison with GSK'157. RIPK1 kinase-dependent necroptosis was induced in immortalized MEFs by 30 min pretreatment with pan-capspases inhibitor ZVAD-fmk (50 µM) and increasing concentrations of the indicated compounds then stimulated by hTNF (20 ng/ml). The TNF-mediated necroptosis was measured at 24 h post-stimulation by SytoxGreen positivity tested

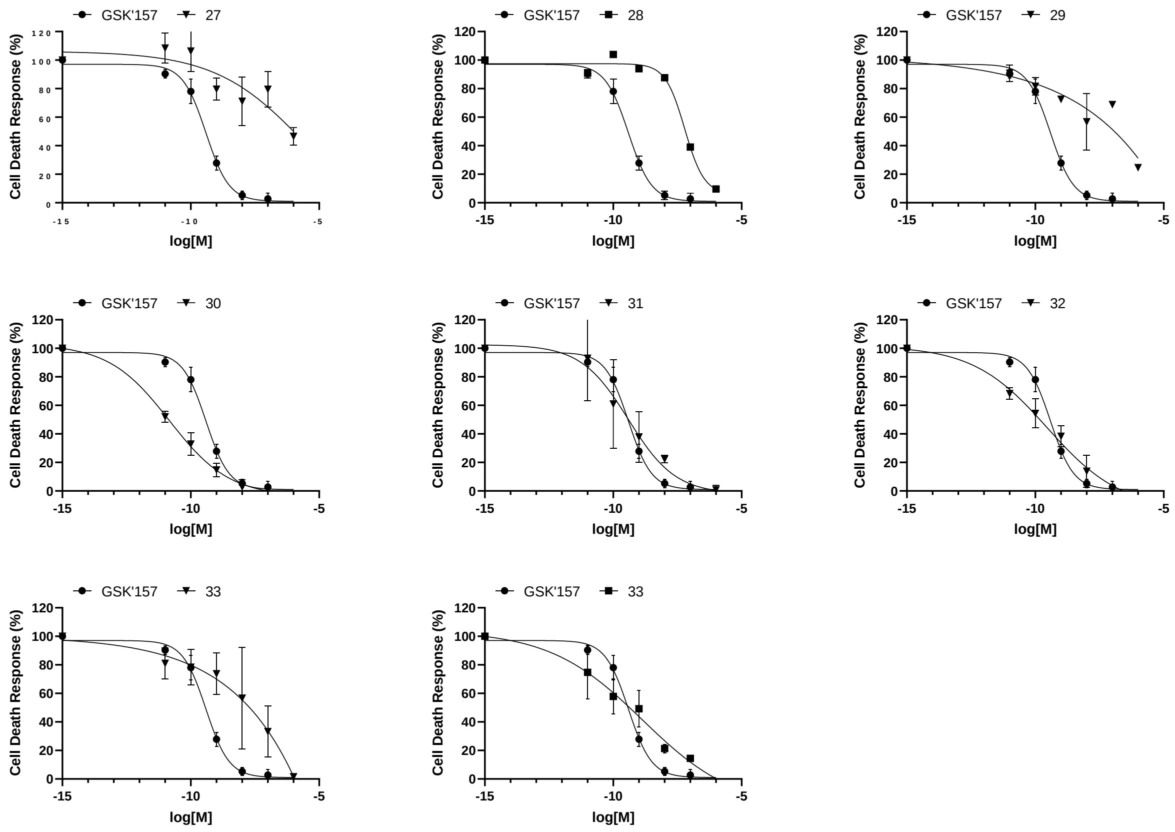

Figure S3. Represensation of the single curve for each compound in comparison with GSK'157. RIPK1 kinase-dependent necroptosis was induced in immortalized MEFs by 30 min pretreatment with pan-capspases inhibitor ZVAD-fmk (50 µM) and increasing concentrations of the indicated compounds then stimulated by hTNF (20 ng/ml). The TNF-mediated necroptosis was measured at 24 h post-stimulation by SytoxGreen positivity tested

References

Axten, J. M., Medina, J. R., Feng, Y., Shu, A., Romeril, S. P., Grant, S. W., et al. (2012). Discovery of 7-methyl-5-(1-{[3-(trifluoromethyl)phenyl]acetyl}-2,3-dihydro- 1H-indol-5-yl)-7H-pyrrolo[2,3-d]pyrimidin-4-amine (GSK2606414), a potent and selective first-in-class inhibitor of protein kinase R (PKR)-like endoplasmic reticulum kinase (PERK). *J. Med. Chem.* 55, 7193–7207. doi: 10.1021/jm300713s.

Axten, J. M., Romeril, S. P., Shu, A., Ralph, J., Medina, J. R., Feng, Y., et al. (2013). Discovery of GSK2656157: An optimized PERK inhibitor selected for preclinical development. *ACS Med. Chem. Lett.* 4, 964–968. doi: 10.1021/ml400228e.

Biscoe, M. R., and Buchwald, S. L. (2009). Selective monoarylation of acetate esters and aryl methyl ketones using aryl chlorides. *Org. Lett.* 11, 1773–1775. doi: 10.1021/ol900295u.

Deng, L., Diao, J., Chen, P., Pujari, V., Yao, Y., Cheng, G., et al. (2011). Inhibition of 1-deoxy-d-xylulose-5-phosphate reductoisomerase by lipophilic phosphonates: SAR, QSAR, and crystallographic studies. *J. Med. Chem.* 54, 4721–4734. doi: 10.1021/jm200363d.

Lumb J., Trevor et al., P. (2004). Pyrrolopyrimidine Derivates, WO2004056830A1.

Molander, G. A., and Gormisky, P. E. (2008). Cross-coupling of cyclopropyl- and cyclobutyltrifluoroborates with aryl and heteroaryl chlorides. *J. Org. Chem.* 73, 7481–7485. doi: 10.1021/jo801269m.

Spiekermann, P. et al. (2019). SHMT INHIBITORS AND USES THEREOF, WO2018106636. *J. Bacteriol.* 10, 1–8. Available at: https://doi.org/10.1016/.
